# The dynamics of cooperation in asymmetric public goods games

**DOI:** 10.1073/pnas.2525760123

**Published:** 2026-01-29

**Authors:** Xiaomin Wang, Christian Hilbe, Boyu Zhang

**Affiliations:** ^a^Laboratory of Mathematics and Complex Systems, Ministry of Education, School of Mathematical Sciences, Beijing Normal University, Beijing 100875, People’s Republic of China; ^b^School of Science, Beijing Forestry University, Beijing 100083, People’s Republic of China; ^c^Max Planck Research Group Dynamics of Social Behavior, Max Planck Institute for Evolutionary Biology, Plön 24306, Germany; ^d^Interdisciplinary Transformation University, Linz 4040, Austria

**Keywords:** evolutionary game theory, cooperation, public goods game, inequality, social dilemma

## Abstract

When people collaborate in groups, they routinely face collective action problems: for the group effort to succeed, individuals need to cooperate despite any incentives to defect. These problems can be modeled with public goods games. To facilitate such an analysis, many studies assume the game is symmetric. Group members have the same means to cooperate (equal endowments), and contributions of different group members are equally effective (equal productivities). Studies that allow for some inequality tend to focus on one kind of inequality only. In practice, however, people can be unequal in many ways. The effect of these inequalities may in turn depend on the specific public goods game considered. To explore these issues, we combine a large-sample experiment with extensive theoretical work. We systematically vary four aspects: the group members’ endowments, their productivities, group size, and whether the public goods game exhibits linear returns or returns given by a threshold function. By exploring all four aspects, we obtain a unique dataset to explore the effect of asymmetry on cooperation. Based on this dataset, we study whether there is an advantage of “aligned inequality”: whether groups achieve a better surplus if more productive individuals have a larger endowment. For public goods games with linear returns, we find such an advantage, thereby corroborating previous research. If returns follow a threshold function, however, aligned inequality results in inferior payoffs. These results show that the effect of inequality on cooperation depends on the kind of public goods game considered.

## Significance

People differ along many economic variables, including their resources and productivity. These differences affect how people cooperate. Here, we use game theory and economic experiments to explore how inequality shapes public good contributions. Results depend on the relationship between individual contributions and the returns of the public good. If the relationship is linear, inequality can promote the group’s total payoff, compared to a control without inequality. But if returns follow a nonlinear threshold function, inequality reduces total payoff. We relate these findings to the individuals’ contribution motives. For linear returns, individuals share the same motives, and contributions are mostly driven by reciprocity. Yet for nonlinear returns, inequality may lead individuals to form different expectations about which equilibrium is most fair.

Humans regularly need to solve collective action problems (1). These problems naturally arise when people collaborate in teams, or when communities need to govern public resources (2–6). In all these instances, individuals might be tempted to free ride on others’ contributions. Such free riders or defectors threaten the very success of the group. Not only do they fail to contribute themselves; they also serve as negative role models that might induce others to defect, too. To analyze the resulting dynamics in a controlled setting, researchers explore how people cooperate in stylized games, such as the repeated public goods game (7–24).

While there are many variants of the public goods game, the general rules are as follows. The game takes place among a group of individuals who interact for many rounds. Each round, all group members receive some fixed endowment, which might be interpreted as their recurring income. Then they decide individually how much of their endowment to contribute to a public good, and how much to keep for themselves. As a result of this interaction, they receive a payoff. Payoffs depend on the endowment the individuals keep for themselves, and on the reward derived from the public good (which is a function of the group members’ contributions). Usually, the public good’s reward function is chosen such that the game is a social dilemma (25, 26). That is, each group member prefers others to contribute more, while simultaneously being tempted to reduce their own contributions. As a result, individuals who contribute more can be seen as being more cooperative.

When exploring cooperation in public goods games, it is common to make some further simplifying assumptions. For example, the game is often taken to be symmetric (7–24). This means that group members are indistinguishable with respect to all their attributes. They receive the same endowments, their contributions are equally productive, and they obtain equal rewards from the public good. This symmetry assumption has important implications for whether or not people cooperate. First, a certain degree of symmetry is likely to favor the emergence of reciprocal cooperation. After all, previous work suggests that successful strategies in repeated games often respond in-kind to their coplayer’s previous behavior (27). In the case of the public goods game, this could mean that individuals contribute their whole endowment if others do so too (16). This kind of reciprocal cooperation, however, seems to require that individuals have comparable endowments. More broadly, symmetry makes it easier for individuals to have a joint understanding of what a fair outcome might be. Once individuals have different endowments or productivities, they might not only differ in whether or not they wish to contribute to the public good, but they might also hold different expectations regarding how much each group member ought to contribute in the given situation.

When theoretical (28–30) or experimental studies (31–40) allow for asymmetry, they often consider individuals who differ along a single dimension. These studies suggest, for example, that asymmetric endowments tend to reduce cooperation (in the following, we treat “asymmetric” and “unequal” as synonyms). Asymmetric productivities, on the other hand, tend to have a neutral or even positive effect. These previous studies provide important insights, yet they do not take into account possible interactions between different kinds of inequality. For a concrete example, consider public goods games in which reward increases proportionally with the total contributions of all individuals. Here, two recent studies find an advantage of aligned inequality (41, 42). These two studies suggest that groups achieve the highest surplus (the highest gain in group payoff, relative to the group’s initial endowment) when more productive individuals receive a larger endowment. However, they leave it open how robust (and beneficial) this positive interaction effect is. For example, once individuals are free to choose their interaction partners, aligned inequality may beget further inequality; after all, more productive individuals prefer interacting with each other (43). Similarly, any positive effects of aligned inequality might only apply to games with linear rewards. For other reward functions (say, a threshold function), unequal endowments and productivities might introduce additional cooperation patterns in which individuals contribute different amounts. Once individuals fail to coordinate on a mutually preferred contribution pattern, cooperation may break down.

To explore the possible dynamics in asymmetric games comprehensively, we consider a more general public good framework. We systematically vary the group members’ endowments, their productivities, the group’s size, and the public good’s reward function. With respect to the reward function, we consider two cases. On the one hand, we consider linear public goods games, where the reward of the public good grows in proportion to the group members’ contributions. This is the kind of reward function used in earlier work to identify an advantage of aligned inequality, refs. 41 and 42. On the other hand, as an example of a nonlinear reward function, we consider threshold public goods games. Here, collective contributions need to surpass a given threshold for all group members to receive a fixed reward (13). Such threshold games have become popular models for collective action problems with a tipping point, such as climate mitigation, where cooperative efforts must reach a minimum level to prevent catastrophic outcomes (4).

**Fig. 1.**
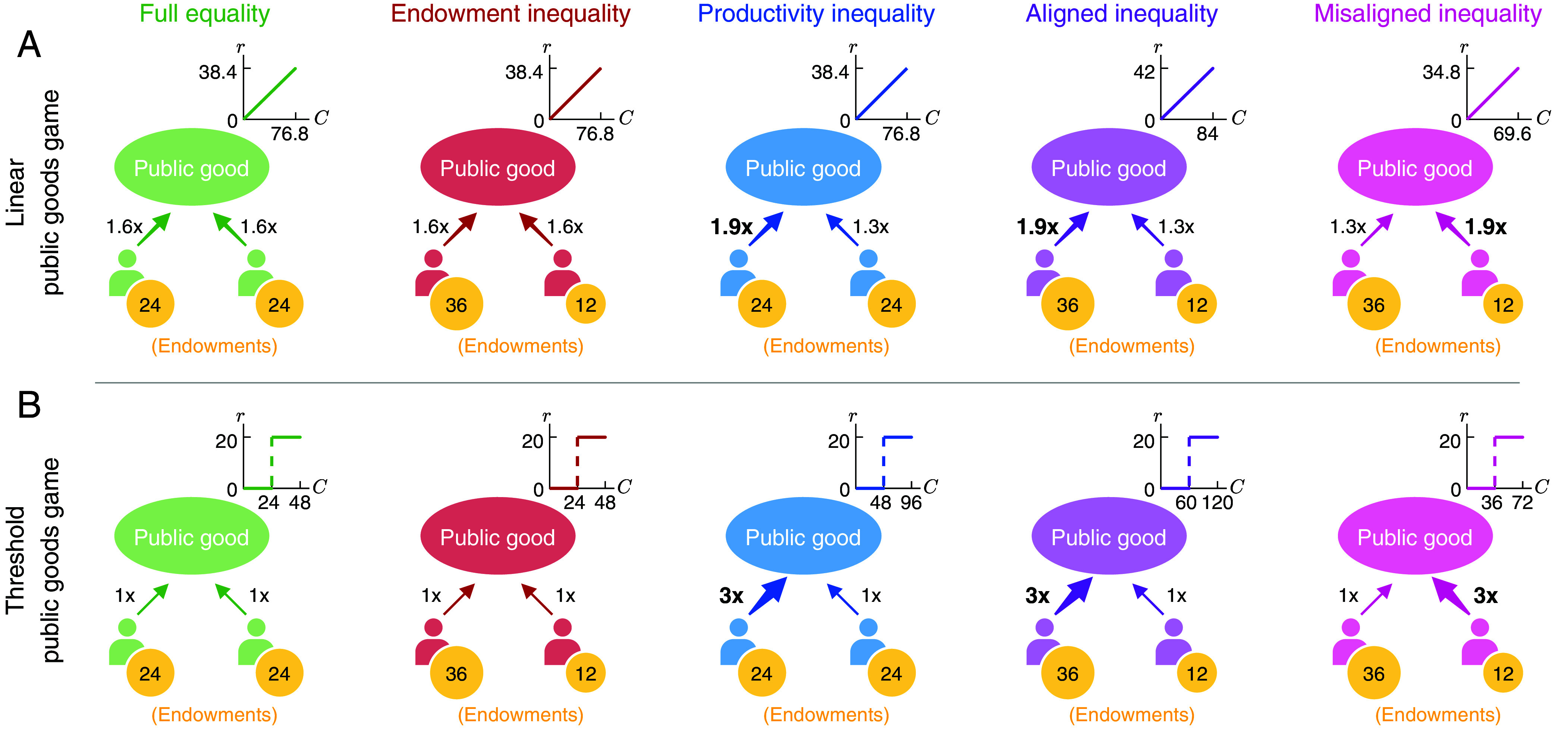
Studying the effect of inequality in two-player public goods games. For our first 10 treatments, we consider public goods games in groups of size two. Each individual receives a fixed endowment each round (indicated by yellow coins). Then they independently decide how much of their endowment to contribute to the public good. An individual’s effective contribution is their contribution multiplied by their individual productivity factor (indicated by arrows). The sum of these effective contributions is the group’s collective contribution (represented by *C*). We systematically vary the individuals’ endowments and their productivity factors. This gives rise to five scenarios. “Full equality” refers to the control scenario. Here, individuals are completely indistinguishable. In “endowment inequality,” individuals only differ in their endowments, whereas in “productivity inequality” they only differ in their productivity. In “aligned” and “misaligned inequality,” individuals differ in both dimensions, with the more productive individual receiving the larger (smaller) endowment, respectively. Moreover, we consider games with two different reward functions. (*A*) In the linear game, the reward increases in proportion to the collective contributions. (*B*) In the threshold game, there is a discontinuous jump in rewards once collective contributions surpass a certain threshold. Note that in each row, the shape of the reward function in the *Inset* remains the same. However, the scaling of the *x*-axis and the *y*-axis may change from one column to the next.

To gain a first intuitive understanding of the individuals’ incentives in these different scenarios, we first compare the games’ Nash equilibria (44). That is, we ask which game outcomes have the property that no single player would like to deviate unilaterally. Then, we explore the impact of different asymmetries with an experiment. For simplicity, we first consider minimal groups of size two, as illustrated in Fig. 1. For those groups, we complement our empirical work with simulations of a game theoretical learning model. In a last step, we then explore public good contributions in larger groups of size four.

Overall, our experimental setup comprises 16 treatments with more than 1,600 (in-person) participants (*Materials and Methods*). As a result, we have a unique dataset to explore the joint impact of different dimensions of inequality, group size, and the public good’s reward function. Based on this dataset, we first corroborate earlier results in the linear public goods game (41). Here, we find that aligned inequality indeed leads to the largest surplus, irrespective of the group’s size (using a subject pool that is rather different from the one in ref. 41). For the threshold public goods game, however, we demonstrate the opposite. Here, unequal endowments diminish total payoff, irrespective of whether endowments are aligned with the participants’ productivities. These results illustrate that the effect of inequality on cooperation depends on the type of the public good’s reward function. While inequality can be beneficial for one reward function, it discourages cooperation for another.

## Results

### Model.

To allow for a simple experimental implementation similar to Hauser et al. (41), we first consider games among two individuals (players). These players interact for many rounds. Every round, each player i∈{1,2} receives an integer-valued endowment $e_{i}$. Players then independently choose their (absolute) contribution to the public good, $c_{i}\in \left\{0,1,...,e_{i}\right\}$. Contributions are multiplied by an individual productivity factor $p_{i}$. We refer to the product $p_{i}\cdot c_{i}$ as the player’s effective contribution. The sum of these effective contributions is the group’s collective contribution, $C=p_{1}c_{1}+p_{2}\;c_{2}$. After having made their contributions, players receive a payoff $\pi_{i}$. This payoff consists of the share of the endowment that players kept for themselves, and of the reward $r_{i}$ derived from the public good,

[1] $$\begin{array}{c} \pi_{i}\left(c_{1},c_{2}\right)=\left(e_{i}-c_{i}\right)+r_{i}. \end{array}$$

The reward $r_{i}$ depends on the public good’s reward function. This function reflects how individual contributions generate public value. In the so-called linear game, rewards are proportional to collective contributions,

[2] $$\begin{array}{c} r_{i}=C/2, \end{array}$$

which may be interpreted as the two players evenly sharing the public good. In the threshold game, players receive some fixed reward *r*, but only if collective contributions reach a predefined threshold *θ*,

[3] $$\begin{array}{c} r_{i}=\left\{\begin{array}{cc} r & \text{if}\;C\geq \theta ,\\ 0 & \text{otherwise}. \end{array}\right. \end{array}$$

This function represents a binary scenario in which the collective effort can either succeed or fail.

In Fig. 1, we depict the two reward functions as insets. The very same figure also illustrates the five scenarios with which we systematically introduce different kinds of asymmetry. The full equality scenario serves as a control. Here, players receive the same endowment and they have the same productivity. In the scenarios with endowment inequality and with productivity inequality, respectively, players differ in one dimension but not in the other. Finally, in the “aligned” (“misaligned”) inequality treatments, players differ in both dimensions. Here, the more productive player receives a larger (smaller) share of the initial endowment. Overall, the two reward functions together with the five scenarios give rise to 10 two-player treatments.

To facilitate comparisons across scenarios, endowments are normalized such that $e_{1}+e_{2}=48$ throughout. As a result, when players get equal endowments, then $e_{1}=e_{2}=24$. When they get unequal endowments, we assume the endowment of the first player is three times the second’s, $e_{1}=36$, $e_{2}=12$. Moreover, all treatments in the linear game have the same average productivity factor, $(p_{1}+p_{2})/2=1.6$. Similarly, in all treatments of the threshold game, we choose a uniform threshold defined by $\theta =(p_{1}e_{1}+p_{2}e_{2})/2$. That is, the group is guaranteed to reach the threshold if both players contribute at least half of their endowment. In that case, we assume they receive the same reward, r=20. These parameters are chosen such that in all treatments there is a tension between collective and individual interests. Collectively, the group achieves the highest total payoff when players make strictly positive contributions. Yet individually, each player prefers to minimize their own contribution while hoping the coplayer contributes more.

### Equilibrium Analysis.

Even though both the linear and the threshold game follow a similar premise, they differ in their strategic nature. In any single round of the linear game, both players have an incentive to reduce their own contributions. Yet by jointly increasing their contributions, they get a higher collective payoff. This setup is similar to the prisoner’s dilemma (27), with the main difference being that we allow for more gradual forms of cooperation. In particular, the linear game is amenable to reciprocity. For example, players may adopt perfectly reciprocal strategies similar to the Tit-for-Tat strategy that won Axelrod’s tournament (27). That is, players could contribute the same fraction of their endowment as their coplayer contributed in the previous round. In this way, the two players would jointly incentivize each other to reach the social optimum, in which both players contribute their full endowment. In this optimum, the resulting payoffs according to Eqs. **1** and **2** are $\pi_{1}=\pi_{2}=\left(p_{1}e_{1}+p_{2}e_{2}\right)/2$. In particular, both players receive the same payoff, irrespective of any prior inequality in their endowments or productivities. These properties make full cooperation (both players contributing their full endowment) a natural focal point (45) (That is, among all possible outcomes, players may consider the full-cooperation outcome to be particularly salient or prominent).

In contrast, in the threshold public goods game, socially optimal outcomes are already feasible in the one-shot (nonrepeated) game. However, this optimum no longer requires players to contribute their full endowment. Instead, collective contributions merely need to meet the threshold *θ*. This can be achieved in many ways, differing in how much each player contributes toward the threshold. The resulting game is a coordination game with many equilibria, and the two players may hold different views on which equilibrium is most fair. For such coordination games, the mechanism of reciprocity is less relevant. In fact, by increasing their own contributions, players may motivate their coplayer to reduce theirs.

**Fig. 2.**
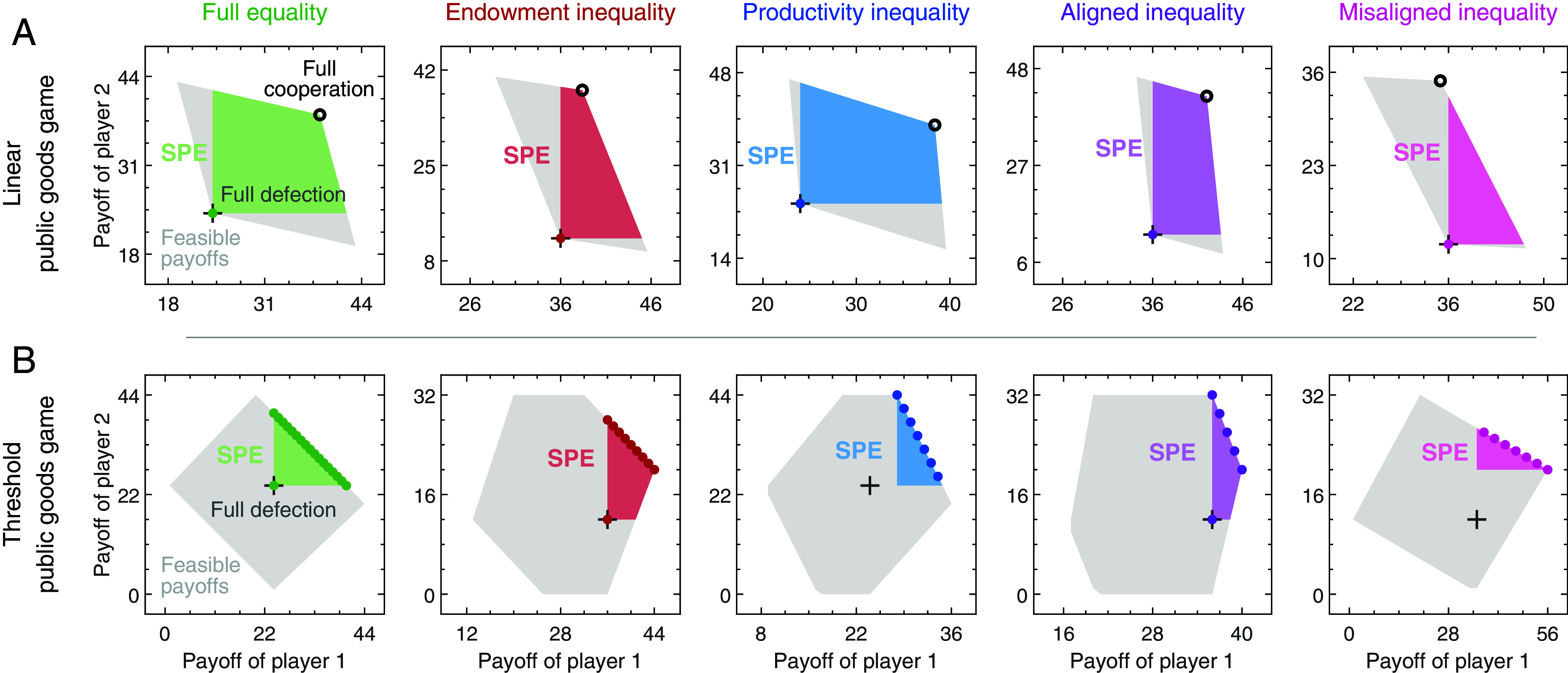
A depiction of the games’ equilibrium payoffs. We use the so-called Folk theorem to characterize the payoffs players may get in a subgame perfect equilibrium (46) of the (infinitely) repeated game. For all of 10 treatments, the respective set of equilibrium payoffs is depicted as a colored area. The gray area indicates which payoffs are feasible in principle, independent of whether or not they can be realized in an equilibrium. For comparison, we also illustrate the payoffs of the pure Nash equilibria of the one-shot game, depicted by colored dots. (*A*) In the linear game, “full cooperation” refers to the outcome where all players contribute their entire endowment in every round (indicated by a circle). This outcome can be realized as a subgame perfect equilibrium in all treatments except misaligned inequality. However, “full defection” (all players contributing nothing, indicated by a plus symbol) is also an equilibrium. (*B*) In the threshold game, socially optimal payoffs can already be achieved in the one-shot (nonrepeated) game. However, players might find it difficult to coordinate on any particular equilibrium, because there are many different ones that are all similarly plausible from the outset. Unequal endowments or productivities may further aggravate these difficulties. For better visibility, we use different *x* and *y* scales for the different panels.

In Fig. 2, we illustrate these considerations by representing the possible equilibrium payoffs across the 10 treatments. To this end, we consider all subgame perfect equilibria. These are all Nash equilibria of the (infinitely) repeated game with the additional property that deviations are never profitable, even if other players have already deviated before (47). In the figure, colored areas depict all payoffs that can possibly arise in such an equilibrium. Colored dots depict the (pure) equilibria of the one-shot game. The figure allows us to make three observations. First, in all treatments, there are infinitely many subgame perfect equilibria (every point within a colored area corresponds to an equilibrium). In particular, the repeated game allows for outcomes in which players make strictly positive contributions. However, in most cases it also allows for full defection, such that no one contributes anything. Second, in the linear game, repeated interactions are crucial for cooperation. Here, the one-shot game only has a single equilibrium in which no one contributes. In contrast, in the threshold game, positive contributions are already feasible if the game is played once. This observation suggests a more prominent role of reciprocal strategies in linear games.

Third, the two game types differ in their focal points. In the linear game, players may naturally attempt to jointly contribute their full endowment (indicated by black circles in Fig. 2*A*). This outcome can be realized as an equilibrium in all but one treatment (only under misaligned inequality, the high-endowment but low-productivity player 1 cannot be incentivized to contribute their entire endowment). Among the four remaining treatments, the one with aligned inequality generates the largest surplus when people fully cooperate (41). In the threshold game, there is a whole line of Pareto-efficient equilibria where collective contributions exactly match the threshold (Fig. 2*B*). Here, asymmetry can make players differ in which equilibrium they prefer. For example, under endowment inequality, players with a high endowment may prefer an equilibrium in which everyone makes the same absolute contribution. Low-endowment players, however, may rather prefer an equilibrium in which everyone gives the same proportion of their endowment. In this way, inequality might make it more difficult to coordinate on any one equilibrium.

To sum up, all treatments allow for many (subgame perfect) equilibria. By introducing inequality in endowments or productivities, we may not only change whether or not a given outcome can be sustained as an equilibrium; we may also affect which equilibrium appears most salient. To explore how humans choose among these equilibria (if at all) in practice, we conduct an economic experiment.

### Analysis of a Behavioral Experiment.

We have recruited 550 subjects to interact in one of the five treatments with a linear reward function (illustrated in Fig. 1*A*). In addition, we have recruited 554 subjects for the five treatments based on the threshold game (as in Fig. 1*B*). In each case, participants play the repeated game twice, in two different sessions. In one session they act as player 1, in the other as player 2 (with a new interaction partner). For the linear game, we follow the protocol of Hauser et al. (41): each session, participants randomly play 20 to 25 rounds to avoid end-game effects. However, only the first 20 rounds are included in the statistical analysis. For the threshold game, we use the protocol of Wang et al. (40): Since end-game effects are less relevant, each game lasts for a fixed length of 20 rounds. Participants were volunteers who were paid in proportion to their earned payoff in the game. All participants were informed beforehand about all relevant aspects of the game (including all players’ endowments and productivities, and the expected duration of the experiment), see *Materials and Methods*.

For our statistical analysis we consider each player pair in each session as an independent observation. Because the two game types have slightly different experimental procedures and key variables, we analyze them separately. In the following, we provide a qualitative summary of our results; for all statistical tests and further quantitative analyses, we refer the reader to *SI Appendix*.

#### Linear public goods game.

**Fig. 3.**
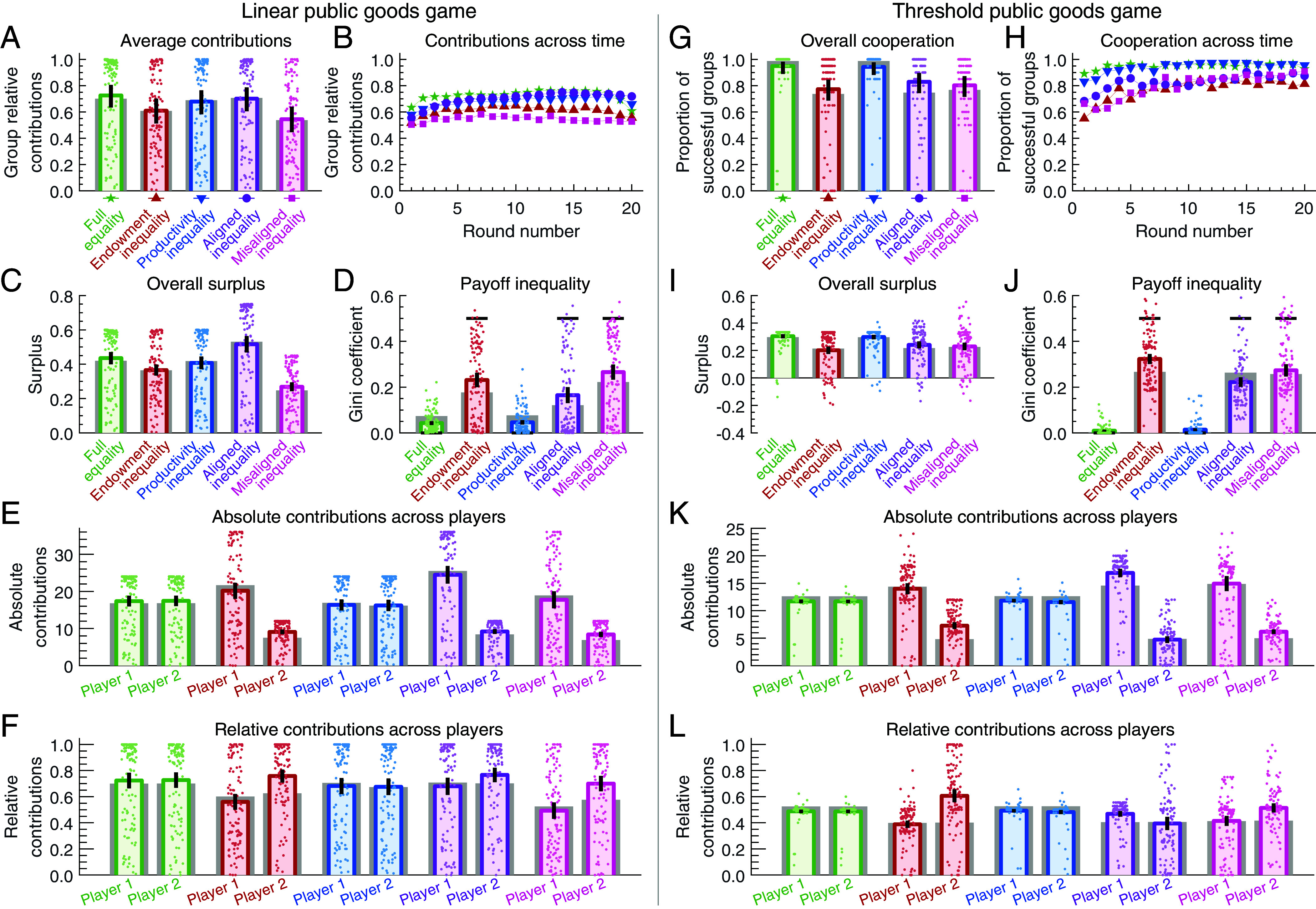
Experimental results for pairwise public goods games. We show results for linear games (*Left*) and threshold games (*Right*). (*A*) In the linear game, average contributions (relative to the players’ endowments) are largest under full equality, productivity inequality, and aligned inequality. (*B*) Contributions are relatively stable over time, except for a positive trend under productivity inequality and aligned inequality. (*C*) Aligned inequality generates the largest surplus. (*D*) In all treatments with unequal endowments, inequality after the game (colored bar) is smaller than the initial inequality (black line). (*E* and *F*) In treatments with unequal endowments, high-endowment players tend to make larger absolute contributions, but smaller relative contributions (relative to their endowment). (*G*) In the threshold games, a key quantity is the proportion of rounds in which the group reaches the threshold. This success rate is largest under full equality and productivity inequality. (*H*) The other three treatments show a particularly poor success rate in the early rounds. (*I* and *J*) As a result, these treatments generate the lowest surplus, and eventual payoffs are most unequal. (*K* and *L*) Contribution patterns are similar to the linear case. However, under aligned inequality, the high-endowment player gives more also in relative terms. Colored dots represent outcomes of individual groups, bars represent averages across all groups. Error bars indicate 95% CI. We compare empirical results (in color) with individual-based simulations (gray bars). The setup of the simulations is explained further below. Simulation parameters are s=1, and (β,γ)=(0,14) for the linear game and (β,γ)=(18,94) for the threshold game.

We begin with the five treatments with a linear reward function. As a first key variable, we compare the group relative contributions $\left(c_{1}+c_{2}\right)/\left(e_{1}+e_{2}\right)$ across treatments, see Fig. 3*A*. We find that three treatments yield the highest contribution levels: full equality, productivity inequality, and aligned inequality. Here, subjects contribute approximately 70% of their endowment. By contrast, contributions in the other two treatments are significantly lower, reaching a minimum of ∼55% for misaligned inequality. To further explore these differences, in Fig. 3*B*, we illustrate players’ contributions over time. Contributions are relatively stable in all treatments, except for a positive trend for productivity inequality and aligned inequality (we establish this positive trend by comparing the players’ contributions during the first five rounds with their contributions during the last five rounds, see *SI Appendix*).

As a next key variable, we consider the group’s overall surplus relative to the total endowments, $\left(\pi_{1}+\pi_{2}-e_{1}-e_{2}\right)/\left(e_{1}+e_{2}\right)$. The higher this variable, the higher are the players’ overall payoffs compared to their initial endowments. Since total endowments are identical across treatments, we henceforth refer to this variable as the group overall surplus. As shown in Fig. 3*C*, aligned inequality generates the largest surplus and misaligned inequality generates the lowest. These results confirm our theoretical prediction, and they reproduce the findings of Hauser et al. (41): If individuals differ in their productivities, they achieve the largest overall payoff if they also have different (but aligned) endowments. Intuitively, aligned inequality is advantageous for two reasons. First, it leads to substantial contributions (Fig. 3*A*); and second, these contributions are more effective (because more of the endowment is allocated to the more productive player).

In addition to the above results, we are also interested in the final inequality among group members. To this end, we compare the Gini coefficient before the game (in the players’ endowments) and after the game (in their payoffs), Fig. 3*D*. In all three treatments in which players have unequal endowments, the coefficient decreases. This reduction is most pronounced in the aligned treatment. To explore these results in more detail, we compare the players’ absolute (Fig. 3*E*) and relative contributions (relative to their endowment, Fig. 3*F*). In all treatments with unequal endowments, we observe the same pattern: players with larger endowment give more in absolute but less in relative terms, compared to their coplayer. The difference in relative contributions is least pronounced under aligned inequality. In this treatment, although the high-endowment players are in a stronger position, they often give their entire endowment. This observation is also reflected in the players’ contributions ($c_{1},c_{2}$) each round (*SI Appendix*, Fig. S2). In the aligned treatment, 43.5% of all rounds end with both players giving their full endowment ($e_{1},e_{2}$). This proportion is much smaller for endowment inequality and misaligned inequality, at 26.1% and 18.3%, respectively.

The cooperation patterns in the linear treatments are in line with what we would expect from reciprocity considerations. In *SI Appendix*, Fig. S4, we relate a player’s contribution in a given round to the coplayer’s contribution in the previous round. We observe a strongly positive correlation in all treatments, for both player 1 and player 2. In addition, in *SI Appendix*, we show that a model with fairness preferences (but without reciprocity) is unable to account for the players’ substantial contributions, see *SI Appendix*, Fig. S19*B*. These observations support the theoretical prediction that reciprocity is important for cooperation in linear public goods games.

#### Threshold public goods game.

In threshold games, perhaps the most important variable is the group success rate—the proportion of rounds in which the group reaches the threshold. According to Fig. 3*G*, there are significantly lower success rates in three treatments, for endowment inequality, aligned and misaligned inequality (i.e., in all three treatments in which participants have different endowments). Again, it is instructive to consider the dynamics over time, see Fig. 3*H*. We find that success rates in these three treatments are particularly low in the first few rounds of the game: here, many groups miscoordinate, by either collectively contributing too much or too little to reach the threshold (*SI Appendix*, Fig. S8). As a result, the three treatments also generate the lowest surplus (Fig. 3*I*). Moreover, almost by design these treatments result in more unequal payoffs (Fig. 3*J*). Overall, these results suggest a negative effect of unequal endowments, independent of whether they are aligned with the participants’ productivities.

With respect to the contributions of each player type, we observe a similar pattern as in the linear treatments. When endowments are unequal, high-endowment players tend to give more in absolute terms but less in relative terms (Fig. 3 *K* and *L*). Interestingly, the only exception (unpredicted by our theory) occurs under aligned inequality. Here the more productive and better-endowed player 1 gives more both in absolute and in relative terms. This explains the greater reduction in the Gini coefficient, compared to misaligned inequality (Fig. 3*J*), despite a comparable success rate in both cases. With respect to the players’ pairwise contributions, the most abundant contribution pair is ($e_{1}/2,e_{2}/2$) in all treatments (*SI Appendix*, Fig. S8). That is, participants typically contribute half their endowment, which is sufficient to match the threshold. Interestingly, this contribution pattern is somewhat inefficient when there is aligned inequality; here the contributions of the high-endowment player 1 would be much more effective (Fig. 1). As a result, in this treatment, we also find a substantial proportion of groups in which only player 1 contributes toward the threshold (which explains why this player also gives more in relative terms).

As expected, reciprocity is less relevant to explain participant behavior in threshold games. If we relate a player’s contribution to the coplayer’s previous contribution, we either find a weak or a negative relationship, see *SI Appendix*, Fig. S10.

#### Summary.

For the 10 two-player treatments, we find that groups with unequal endowments tend to achieve less cooperation. The only exception occurs when two conditions are met simultaneously: i) unequal endowments are aligned with the participants’ productivities, and ii) the public good’s reward function is (approximately) linear. Previous work that reported positive effects of inequality met both of these criteria (41, 42). Our work corroborates that aligned inequality is advantageous in linear games. However, it also suggests that these positive effects of aligned inequality do not necessarily carry over to other public goods games with different reward functions.

### Individual-Based Simulations of a Learning Model.

According to the above empirical analysis, human behavior in repeated public goods games is driven by at least two mechanisms. First, especially in linear games, a substantial proportion of decisions is explained by reciprocity (*SI Appendix*, Fig. S4). Participants take into account their coplayer’s previous behavior, and they react accordingly. Second, among many equally plausible equilibria, participants seem to prefer outcomes in which players contribute equally (in absolute or relative terms, see *SI Appendix*, Figs. S2 and S8). In the following, we propose an individual-based learning model that incorporates both aspects.

To take into account reciprocity, we let players adopt reactive strategies (48–52). That is, when deciding what to contribute next, players respond to their coplayer’s previous contribution. As in the experiment, players can make any integer contribution $c_{i}\in \left\{0,1,\ldots ,e_{i}\right\}$. Therefore, we generalize the learning model by Hauser et al. (41), which only allowed players to either give their full endowment or nothing at all. Allowing for all possible contributions is particularly important for threshold games. There, many of the interesting equilibria are only feasible if individuals may contribute arbitrary amounts between 0 and $e_{i}$.

To take into account the empirical observation that participants value equal contributions, we consider an appropriate utility function. We assume the utility of player *i* in a round with contributions ($c_{1},c_{2}$) is given by

[4] $$\begin{array}{c} u_{i}\left(c_{1},c_{2}\right)=\pi_{i}\left(c_{1},c_{2}\right)-\beta \frac{|c_{1}-c_{2}|}{\max\{e_{i}\}}-\gamma \left|\frac{c_{1}}{e_{1}}-\frac{c_{2}}{e_{2}}\right|. \end{array}$$

The first term on the right hand side represents *i*’s payoff, as described by Eq. **1**. The second term represents the player’s aversion against unequal absolute contributions. The larger the parameter β≥0, the more players aim to choose contributions such that $c_{1}\approx c_{2}$. Similarly, the third term represents aversion against unequal relative contributions. The parameter γ≥0 controls to which extent players prefer contributions with $c_{1}/e_{1}\approx c_{2}/e_{2}$. For β=γ=0, we recover the standard case without any preferences for equal contributions of any kind. Here, players only maximize their payoffs.

To model how individuals update their strategies over time, we follow earlier work on linear public goods games (41, 42). We assume individuals learn new strategies based on introspection dynamics (53, 54). Compared to the classical pairwise comparison process (55), introspection dynamics has the advantage of being applicable to arbitrary (asymmetric) games (56, 57). It assumes that at regular time intervals, players compare their current utility to the utility they could have obtained with a randomly chosen alternative strategy. The larger the utility of the alternative, the more likely it is adopted. This leads to a time series where the two players regularly adapt their strategies to improve their respective utilities. We explore this dynamics with simulations, run for all treatments, and for many different parameter values, β∈{0,1,…,30} and γ∈{0,1,…,100}.

We ask for which parameter combination we get the best fit to the empirical data. We use two approaches. First, we seek for optimal parameters (β,γ) for each of the two reward functions separately (for the linear game, and for the threshold game). In each case, we look for the parameter combination with the least squared error between the empirical surplus (Fig. 3 *C* and *I*) and the surplus estimated from simulations, across all five treatments. By allowing the optimal parameters (β,γ) to differ between the two reward functions, we account for the possibility that the different objectives (of equal absolute or equal relative contributions) may differ in how salient they are. For the linear game, we obtain the best fit for β=0, γ=14. These numbers suggest that we can already explain much of the empirical regularities by assuming that individuals care about relative contributions. In contrast, in the threshold game we observe that many (β,γ) pairs provide a similarly good fit between simulations and data. For all of them, both β>0 and γ>0, see also *SI Appendix*, Fig. S14. In Fig. 3, we show the respective simulation results with gray bars (based on the estimated optimal *β* and *γ* values for each reward function). In most cases, there is a surprisingly good visual agreement between these simulations and the experimental data (colored bars).

As our second approach, we also look for one pair (β,γ) that provides the best fit across all 10 treatments. Here, we obtain the best fit for β=4 and γ=20 (*SI Appendix*, Fig. S14*C*). However, the respective overall fit with the data is less convincing. For example, the respective simulations tend to overestimate the groups’ success rates in threshold games with unequal endowments (*SI Appendix*, Fig. S18*G*).

In sum, the individual-based simulations are able to recover many of the empirical patterns (as suggested by the fit between gray and colored bars in Fig. 3). They suggest a substantial role for reciprocity, and a preference for equal (relative) contributions. (For analogous simulations that ignore the role of reciprocity, see *SI Appendix*, Fig. S19*B* for the linear game and *SI Appendix*, Fig. S20*B* for the threshold game).

### Beyond Pairwise Games.

So far we considered games in minimal groups of size two. This reduced setup allowed for an intuitive analysis, and it made it straightforward to compare our results to the pairwise linear games studied by Hauser et al. (41). However, to get a broader perspective on cooperation in public goods games, it is important to also explore behavior in larger groups. After all, certain effects may only become apparent in groups of nontrivial size (e.g., one person completely free riding while others aim to maintain cooperation among each other).

To explore the effect of group size, we ran additional experiments for games among four players. To this end, we recruited another 312 participants to interact in a linear game, and 276 participants for the threshold game. To have sufficient coverage, we only considered three treatments for each reward function: full equality, aligned inequality, and misaligned inequality. Moreover, to be comparable to the two-player setup, we opted for a design in which the player types of the pairwise games are duplicated. For example in the two-player setup, aligned inequality meant that one player had a large endowment and high productivity, whereas the other player had a small endowment and low productivity. Now in the 4-player treatments, there are two players with an equally large endowment and equally high productivity, and two other players who both have the same small endowment and low productivity (*Materials and Methods*).

**Fig. 4.**
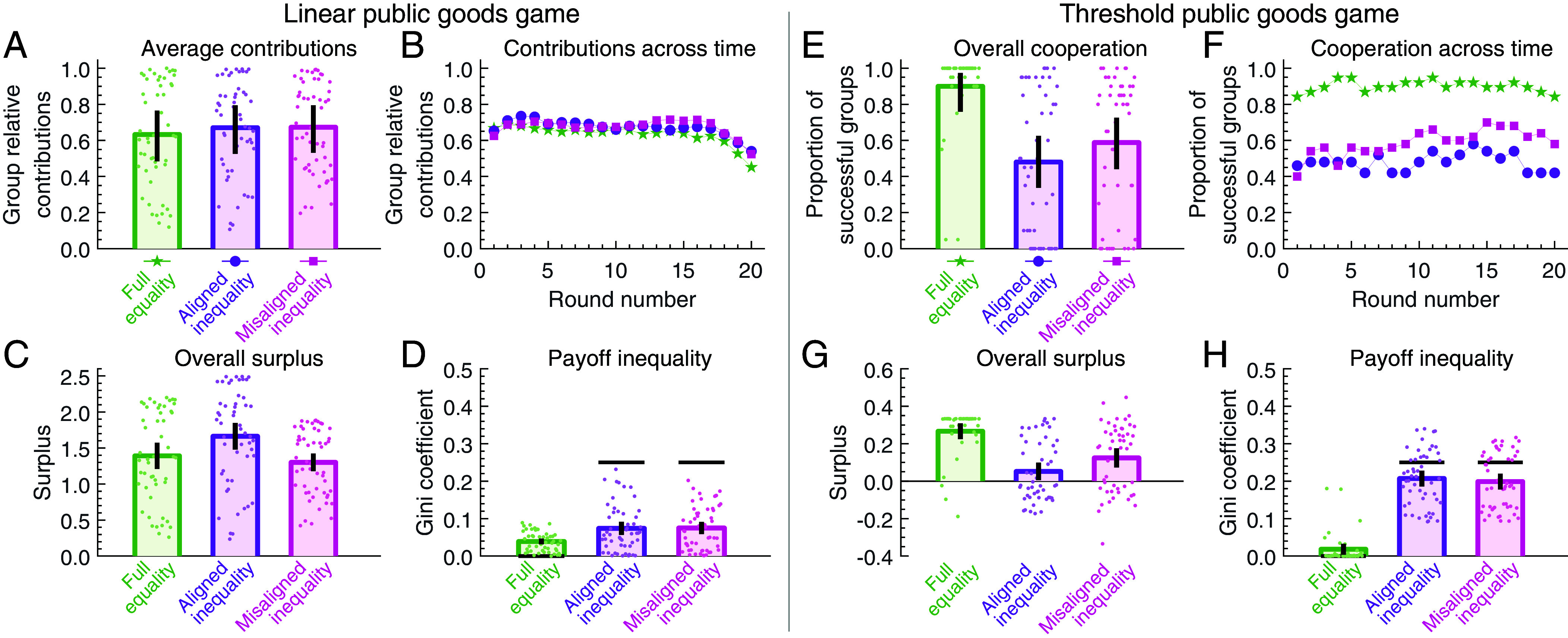
The effect of inequality in groups of size four. Same as Fig. 3 but for full equality, aligned inequality, and misaligned inequality treatments in the 4-player setting. (*A*) In the linear game, average contributions are similar across the three treatments. (*B*) Contributions remain stable over time, except for a decreasing trend under full equality. (*C*) Aligned inequality generates the highest surplus. (*D*) Both asymmetric treatments lead to a comparable reduction in inequality. (*E*) In the threshold games, the success rate is highest under full equality, and notably lower under aligned inequality and misaligned inequality. (*F*) Both asymmetric treatments show low success rates across all rounds. (*G* and *H*) Consequently, the two asymmetric treatments yield a similarly low surplus, and payoff inequality remains.

Experimental results are shown in Fig. 4. We observe interesting qualitative differences to the earlier two-player experiments (discussed in more detail in *SI Appendix*). For example, in the linear game, aligned and misaligned inequality now yield similar contributions (Fig. 4*A*). Moreover, in the threshold game, aligned and misaligned inequality have a more persistently negative effect on coordination (Fig. 4*F*). However, with respect to the overall surplus, we recover our previous results. In the linear game, aligned inequality again generates the largest surplus among all treatments (Fig. 4*C*). In contrast, in the threshold game, both aligned and misaligned inequality lead to a reduced surplus, compared to the control without any inequality (Fig. 4*G*). For linear games, these findings generalize previous results on the positive effects of aligned inequality (41) to games in larger groups. At the same time, they again demonstrate that the same kind of inequality yields inferior results in threshold games.

## Discussion

Most important collective action problems today involve some form of asymmetry. In many examples, such as teamwork in companies (58) or international efforts to mitigate climate change (59), actors differ in their ability or their incentives to contribute to a group effort. Such asymmetries can affect whether groups succeed or fail. To explore these issues, there is a growing literature on the effect of asymmetry on cooperation (28–42). Most studies, however, are either based on a single public good paradigm, or they assume there is only asymmetry in one dimension. Here, we provide a more comprehensive analysis of asymmetric public goods games. To this end, we compile and analyze a unique dataset, based on experiments that vary the participants’ endowments, their productivities, group size, and the public good’s reward function. These distinctions give rise to sixteen treatments. We analyze these treatments empirically, as well as with equilibrium methods and individual-based simulations.

We consider two types of reward functions. In the linear game, the rewards of collective action are proportional to the individuals’ contributions (as in refs. 41 and 42). In particular, the effect of one individual’s contribution is independent of the contributions of others. In the threshold game, on the other hand, there is only a substantial reward when contributions surpass a certain level (13). This game can describe scenarios with a tipping point (4). Here, the effect of one individual’s contributions critically depends on how much others contribute. We find that the effect of inequality differs substantially between the two game types. For the linear game we corroborate previous results (41, 42) that unequal endowments can be beneficial (Fig. 3*C*). Such a case can arise when individuals differ in how productive their contributions are, and when endowments and productivities are aligned (more productive individuals receive the larger endowment). In contrast, in threshold games, treatments with unequal endowments always result in a lower surplus (Fig. 3*I*).

These differences can be related to the players’ contribution motives. In the linear game, cooperation is driven by reciprocity (*SI Appendix*, Fig. S4) and by a preference for equal relative contributions. Both aspects help to support cooperation under aligned inequality. Here, even large-endowment players may find it worthwhile to contribute their entire endowment—provided the low-endowment player does the same. In contrast, in threshold games, reciprocity considerations are largely irrelevant (*SI Appendix*, Fig. S10). Instead, players coordinate on an equilibrium of the nonrepeated game. Endowment inequality can render this coordination process more difficult. For example, some participants might prefer all group members make the same absolute contribution. Others may find it more appropriate if contributions are proportional to endowments. These different views impede coordination, especially in the early phase of the game when participants have no prior experience yet about the behavior of their coplayer (Fig. 3*H*).

Importantly, we consider a type of threshold game where the participants’ collective contributions need to surpass a certain threshold. Alternatively, models of evolutionary game theory often consider alternative forms of threshold games. There, individuals can only decide whether to cooperate or defect, and it takes a sufficient number of cooperators for the group to reach the threshold (60–62). While we do not explore such games with binary action spaces, we believe they too can serve as valuable models to explore the effect of inequality (e.g., via asymmetric cooperation costs).

To explore how various sources of inequality shape cooperation in public goods games, we have neglected many additional complexities that are relevant in applications. For example, we have neglected uncertainty, assuming that the shape of the public good’s reward function is common knowledge. Similarly, we have also neglected possible asymmetries in how rewards are distributed among group members (63), or environmental stochasticity, kinship, and social bonds (64–66), which all can affect cooperative behavior. Nevertheless, by simultaneously varying multiple sources of inequality, group size, and the public good’s reward function, we provide valuable insights into the determinants of human behavior in public good experiments.

## Materials and Methods

### Model.

In the most general case, we consider repeated public goods games among *n* players with varying endowments or productivities. In each round, player *i* receives an endowment $e_{i}$ and decides which contribution $c_{i}$ to make toward the public good. Contributions are multiplied by an individual productivity factor $p_{i}>0$. The group’s collective contribution is $C=\sum p_{i}\;c_{i}$. This collective contribution determines the players’ reward $r_{i}$ from the public good, and hence their payoffs $\pi_{i}=e_{i}-c_{i}+r_{i}$. The exact value of $r_{i}$ depends on the public good’s reward function. In the linear game, players equally share the group’s collective contribution, such that $r_{i}=C/n$. This setup leads to a social dilemma when $1<p_{i}<n$. In the threshold game, each player receives a fixed positive reward $r_{i}=r$ if the group’s collective contribution meets or exceeds a predefined threshold *θ*. If the reward *r* is sufficiently large, positive contributions can already be sustained in the one-shot (nonrepeated) game. However, players may differ in their views on how much each should contribute.

We define payoffs of the repeated game as the player’s average payoff per round. For much of this paper we focus on two-player games with integer contributions. To explore how group size affects our results, however, we also consider groups of four players.

### Equilibrium Analysis.

In Fig. 2, we illustrate the sets of equilibrium payoffs in two-player games, both for the one-shot and the infinitely repeated setting. In the one-shot game, a payoff vector $\left(\pi_{1},\pi_{2}\right)$ corresponds to a Nash equilibrium if neither player can unilaterally enforce a higher payoff. For the repeated game, we apply the Folk theorem (46). This theorem characterizes the set of subgame perfect equilibrium payoffs when players are sufficiently patient. According to the Folk theorem, any payoff vector that is feasible and individually rational can be sustained in a subgame perfect equilibrium. A payoff vector ($\pi_{1},\ldots ,\pi_{n}$) is feasible if there exists a sequence of contributions such that the respective average payoffs converge to that payoff vector. The payoff vector is individually rational if each player receives at least as much as they can guarantee themselves, regardless of the other players’ contributions. Hence, many payoff vectors that are not Nash equilibria of the one-shot game can still be sustained by a subgame perfect equilibrium in the repeated setting. For instance, the payoff corresponding to full cooperation (all players contributing their entire endowment each round) may be sustained as an equilibrium outcome in the repeated linear game. We use the same approach to characterize equilibrium payoffs in the four-player game. The corresponding analysis is provided in *SI Appendix*.

### Behavioral Experiment.

For the games among two players, we conducted experiments for both the linear and the threshold game for five treatments: full equality, endowment inequality, productivity inequality, aligned inequality, and misaligned inequality (Fig. 1). Participants were students from Beijing Normal University who attended in person in the university’s computer lab. The experiment complied with all relevant ethical regulations and was approved by the Ethics Committee of the Medical Faculty of Kiel University (D 613/21). All participants gave their informed consent before participation. They were randomly assigned to one of the game types (linear game or threshold game), and subsequently to a treatment. Throughout the experiment, participants made their decisions independently and were not permitted to communicate. For the linear game, the number of participants in the five treatments were 114, 110, 110, 106, and 110, respectively. For the threshold game, the numbers were 110, 118, 112, 104, and 110. The data for the linear game and for two treatments of the threshold game (aligned inequality and misaligned inequality) were specifically collected for this study. For the other three treatments of the threshold game, we reanalyze behavioral data from Wang et al. (40).

In each treatment, all experimental parameters are common knowledge. Each treatment consists of two sessions. At the beginning of session 1, participants are randomly paired and assigned the roles of player 1 and player 2. Pairs remain fixed throughout the session. Each session lasts for multiple rounds. After each round, participants learn their coplayer’s contributions and the resulting payoffs. After session 1, participants are informed that they would take part in session 2 with a new coplayer, and that they will be assigned the opposite role from what they had in session 1. Apart from switching roles, the rules of session 2 are the same as in session 1. In the linear game, participants play 20 to 25 rounds per session to avoid end-game effects, but only the first 20 rounds are analyzed. In the threshold game, participants play 20 rounds per session. After the game, participants fill out a survey. The survey for the linear game focuses on preferred contribution patterns and conditional contributions, using the strategy method. The survey for the threshold game focuses on fairness perceptions and on expectations for the coplayer’s minimum contribution based on successful coordination. To encourage participants to take the experiment seriously, participants receive a fixed show-up fee plus a bonus proportional to their total payoffs. On average, they earned 64.07 Yuan (∼8.94 EUR) in the linear game, and 61.01 Yuan (∼8.52 EUR) in the threshold game.

To explore group size effects, we also conducted four-player versions of both games, using three treatments for each reward function: full equality, aligned inequality, and misaligned inequality. The four-player games mirror the two-player design, with each possible role being duplicated (i.e., there are two players of each original type). The overall structure matches the two-player setting. A total of 588 students participated in the four-player experiment. Average earnings were 53.24 Yuan (∼6.52 EUR) for the linear game and 56.96 Yuan (∼6.97 EUR) for the threshold game.

We analyze the data using two-tailed nonparametric tests, treating groups of interacting players as statistical units. For each outcome variable (such as the group overall surplus), we calculate the 20-round average value for each group of players. Then we make comparisons either across treatments or within the same treatment. For comparisons across treatments, we use the Mann–Whitney–Wilcoxon test. For within-treatment comparisons, we use the Wilcoxon signed-rank test. For further details on our procedures and statistical results, see *SI Appendix*.

### Individual-Based Simulations.

We analyze the repeated two-player game using a game theoretical learning model. We assume players adopt reactive strategies (48–52). They determine their contribution based on their coplayer’s previous contribution. A reactive strategy of player 1 is represented by a vector $R^{1}=\left(c^{1};c_{0}^{1},c_{1}^{1},...,c_{i}^{1},...,c_{e_{2}}^{1}\right)$. Here, $c^{1}$ indicates the player’s initial contribution in the first round. The other entries $c_{i}^{1}$ denote player 1’s contribution in response to the coplayer’s contribution *i* in the previous round. Each component is an integer from player 1’s action set $\left\{0,1,...,e_{1}\right\}$. Analogously, player 2’s strategy is represented by a vector $R^{2}$. Because strategies are deterministic, the vectors $R^{1}$ and $R^{2}$ uniquely determine the players’ contributions for an arbitrarily long sequence of rounds.

To allow for fairness preferences, we assume player *i* evaluates round-wise outcomes based on the utility function $u_{i}\left(c_{1},c_{2}\right)$ defined in Eq. **4**. When choosing their strategy, players aim to maximize their utility.

To model how asymmetric players update their strategies over time, we use introspection dynamics (53, 54). The specific update steps are as follows. In each time step, pairs of players engage in twenty rounds of the game (as in the experiment). Then, one of the two players is randomly chosen to reconsider their strategy. The selected player *i* randomly draws an alternative reactive strategy ${\tilde{R}}^{i}$. This player compares their realized 20-round utility $u_{i}$ with the 20-round utility ${\tilde{u}}_{i}$ the player could have obtained by playing the alternative strategy instead (keeping the coplayer’s strategy unchanged). The player then switches to the alternative strategy ${\tilde{R}}^{i}$ with probability $\varphi \left({\tilde{u}}_{i},u_{i}\right):=(1+\exp\left(-s\left({\tilde{u}}_{i}-u_{i}\right)\right){)}^{-1}.$ Here, the parameter s≥0 reflects the strength of selection. The larger *s*, the more players are biased to adopt alternative strategies with a large utility.

Introspection dynamics gives rise to a Markov process on the space of all strategy profiles $\left(R^{1},R^{2}\right)$. For any finite selection strength *s*, this process has a unique invariant distribution, which is independent of the players’ initial strategies. However, the large size of our strategy space renders an exact calculation of this distribution infeasible. Instead, we run individual-based simulations for $10^{7}$ time steps, recording the players’ contribution profiles. This allows us to compute all further quantities of interest.

To quantify the agreement between simulation results and experimental data, we define an objective function. We focus on the group overall surplus (GOS), a metric that is well defined for both game types (linear and threshold public goods games, as illustrated in Fig. 3 *C* and *I*). For each game type, we optimize a separate objective function $\Delta_{\mathrm{GOS}}$, given by

$$\begin{array}{c} \Delta_{\mathrm{GOS}}=\sqrt{\sum_{k\in K}{\left(GOS_{\exp}^{k}-GOS_{\mathrm{sim}}^{k}\right)}^{2}}. \end{array}$$

Here, $GOS_{\exp}^{k}$ denotes the average surplus observed in treatment *k* in the experiment, K={FE, EI, PI, AI, MI} denoting the five treatments, while $GOS_{\mathrm{sim}}^{k}$ is the corresponding average surplus from the simulations, computed across all rounds.

We varied the parameters and ran separate simulations for each game. Specifically, we tested values of selection strength s∈{1,10,100}, preference weights β∈{0,1,…,30} and γ∈{0,1,…,100}. Then we search for the optimal parameter set (s,β,γ) that minimizes $\Delta_{\mathrm{GOS}}$, for each of the two game types. Based on the optimal parameter set, we analyze further quantities to assess to which extent simulations align with the experimental findings (see gray bars in Fig. 3).

Instead of estimating the optimal values (s,β,γ) separately for each game type, we also explored whether a single set of parameters could jointly account for behavior across all 10 two-player treatments. As shown in *SI Appendix*, the respective best fit is less convincing, compared to the setup in which we estimate the optimal parameters separately for each reward function.

## Supplementary Material

Appendix 01 (PDF)

## Data Availability

Results were analyzed and visualized with Matlab R2020b and StataSE13. The experimental data and computer code can be found in Zenodo (https://doi.org/10.5281/zenodo.16918146, 67).
