# Supplementary material for: The dynamics of cooperation in asymmetric public goods games
Source: Proc Natl Acad Sci U S A. 2026 Jan 29;123(5):e2525760123. doi: 10.1073/pnas.2525760123 (PMC12867639; doi:10.1073/pnas.2525760123)
Supplement: Supplementary file 1 — Appendix 01 (PDF) [file pnas.2525760123.sapp.pdf]

# Supporting Information for

## The dynamics of cooperation in asymmetric public goods games

**Xiaomin Wang, Christian Hilbe and Boyu Zhang**

**Corresponding authors**
**Christian Hilbe, E-mail: christian.hilbe@it-u.at**
**Boyu Zhang, Email: zhangby@bnu.edu.cn**

### This PDF file includes:

Supporting text
Figs. S1 to S24
Tables S1 to S26
SI References

## Supporting Information Text

## Contents


**1 Asymmetric public goods games among two players** **3**
- A Description of the linear public goods game . . . 3
- B Description of the threshold public goods game . . . 3
- C Different sources of inequality . . . 3
- D Nash equilibrium analysis . . . 3

**2 Behavioral experiment of the two-player public goods games** **4**
- A Description of the experimental design . . . 4
- B Description of the experimental procedures . . . 5
- C Data analysis . . . 5
- D Experimental results of the linear game . . . 5
- E Experimental results of the threshold game . . . 8

**3 Individual-based simulations** **10**
- A Reactive strategies . . . 10
- B Utility function . . . 10
- C Introspection dynamics . . . 11
- D Simulation methods . . . 11
- E Parameters estimation . . . 11
- F Numerical results . . . 12

**4 Four-player public goods games** **13**
- A Model and Nash equilibrium analysis . . . 13
- B Behavioral experiment . . . 15
- C Experimental results of the linear game . . . 16
- D Experimental results of the threshold game . . . 18

**5 Further materials** **19**
- A Informed consent for behavioral experiment . . . 19
- B Instructions of the two-player linear public goods game . . . 20
- C Instructions of the two-player threshold public goods game . . . 23
- D Instructions for the four-player linear public goods game . . . 24
- E Instructions for the four-player threshold public goods game . . . 26

## 1. Asymmetric public goods games among two players

**A. Description of the linear public goods game.** To gain some first intuition on the possible effects of asymmetry, we first consider games with two players only. In the (one-shot) linear public goods game, each player is allocated an integer-valued endowment, denoted by $e_i$ for player $i$ (with $i = 1, 2$). Out of this endowment, the two players decide independently on the amount $c_i$ they wish to contribute to the public good. The absolute contribution $c_i$ is an element of the following sets,

$$\begin{aligned} S_1 &= \{0, 1, \ldots, e_1\}, \\ S_2 &= \{0, 1, \ldots, e_2\} \end{aligned} \qquad [1]$$

for player 1 and player 2, respectively. Each player's contribution $c_i$ is multiplied by a productivity factor $p_i$ with $1 < p_i < 2$ for $i \in (1, 2)$. This factor reflects the impact of each player's contribution to the public good. The *group collective contribution* to the public good is the sum of the individual contributions multiplied by their respective productivity factors, represented as $C = p_1c_1 + p_2c_2$. After both players have made their decisions, the generated public good is distributed equally among the two players. As a result, the payoff $\pi_i(c_1, c_2)$ for each player is the sum of what they retain of their initial endowment and their share from the public good:

$$\pi_i(c_1, c_2) = e_i - c_i + \frac{p_1c_1 + p_2c_2}{2}. \qquad [2]$$

We refer to $c_i/e_i$ as the player's *relative contribution* and $(c_1 + c_2)/(e_1 + e_2)$ as the *group relative contribution*. The *group overall surplus*, $(\pi_1 + \pi_2 - e_1 - e_2)/(e_1 + e_2)$, quantifies to which extent the players' total payoffs exceed their initial endowments.

**B. Description of the threshold public goods game.** The other paradigm we use to explore contributions to public goods is the threshold game. The payoff structure of the one-shot threshold game is similar to the linear game. The primary difference is in how the players' returns from the public good are calculated. Here, we adopt the two-player threshold game model described in Wang et al. (1). Here, the players' payoffs depend on how their collective contributions compare to a pre-defined threshold $\theta > 0$. If the group collective contribution matches or exceeds the threshold $\theta$, each player gets a reward $r_i > 0$. Otherwise, the reward is zero. As a result, the players' payoffs are

$$\pi_i(c_1, c_2) = \begin{cases} e_i - c_i + r_i & \text{if} \quad p_1c_1 + p_2c_2 \geq \theta, \\ e_i - c_i & \text{otherwise.} \end{cases} \qquad [3]$$

We assume that the threshold satisfies the condition

$$\theta \leq \min\{e_1, r_1\} \cdot p_1 + \min\{e_2, r_2\} \cdot p_2, \qquad [4]$$

This condition ensures that the threshold can be reached at all, and that both players have some incentive to reach it (1). The definition of *group overall surplus* is the same as in the linear game described in Section 1A. However, the surplus generated can be negative in the threshold game.

**C. Different sources of inequality.** For our experiments, introduce two sources of inequality. The two players can either differ in their endowments, in their productivities, or both. For the linear public goods game paradigm, our experiment is similar to Hauser et al. (2) (but with slightly different endowment values). In the treatment of full equality (FE), both players have the same initial endowments and productivity factors. The endowment inequality (EI) treatment features players with different initial endowments yet the same productivity factors. Conversely, the productivity inequality (PI) treatment involves players with different productivity factors but equal initial endowments. In the aligned inequality (AI) treatment, the player with the higher endowment is more productive. By contrast, in the misaligned inequality (MI) treatment, the player with the lower endowment is more productive. For the threshold public goods game paradigm, we extend the experiment by Wang et al. (1). While that study only investigated inequality in a single dimension, our study now allows for two-dimensional inequality, where players differ simultaneously in their endowments and productivities. The precise parameter values used for each experimental treatment are summarized in **Table S1**.

Although our design centers on economic inequality within the game, it is worth noting that other forms of inequality have also been shown to influence cooperation, such as unequal scaling factors that modulate the mapping from payoffs to fitness and thereby generate status- or resource-based asymmetries among individuals (3–5).

**D. Nash equilibrium analysis.** The one-shot linear game has a unique Nash equilibrium, according to which all players contribute nothing. For the repeated linear game, Hauser et al. (2) have shown that full cooperation is a subgame perfect equilibrium in all our experimental treatments, except for the misaligned inequality treatment.

In the one-shot threshold game there can be two types of equilibria. On the one hand, one possible equilibrium outcome is that all players keep their endowment, to which we refer as the defective equilibrium. On the other hand, there is also a set of cooperative Nash equilibria in which the group collective contribution exactly matches the threshold. For the parameters of the experiment, cooperative Nash equilibria exist in all five treatments. However, the defective Nash equilibrium only exists in the treatments with full equality, endowment inequality and aligned inequality. For the repeated threshold game, we analyze the subgame perfect equilibrium using the Folk theorem (6). In **Fig. 2** of the main text, the colored area represents all possible payoffs that can be achieved in some subgame perfect equilibria.

| | | | Full equality (FE) | Endowment inequality (EI) | Productivity inequality (PI) | Aligned inequality (AI) | Misaligned inequality (MI) |
|---|---|---|---|---|---|---|---|
| **Linear PGG** | Endowments | $e_1$ | 24 | 36 | 24 | 36 | 36 |
| | | $e_2$ | 24 | 12 | 24 | 12 | 12 |
| | Productivities | $p_1$ | 1.6 | 1.6 | 1.9 | 1.9 | 1.3 |
| | | $p_2$ | 1.6 | 1.6 | 1.3 | 1.3 | 1.9 |
| **Threshold PGG** | Endowments | $e_1$ | 24 | 36 | 24 | 36 | 36 |
| | | $e_2$ | 24 | 12 | 24 | 12 | 12 |
| | Productivities | $p_1$ | 1 | 1 | 3 | 3 | 1 |
| | | $p_2$ | 1 | 1 | 1 | 1 | 3 |
| | Rewards | $r_1 = r_2$ | 20 | 20 | 20 | 20 | 20 |
| | Threshold | $\theta$ | 24 | 24 | 48 | 60 | 36 |

**Table S1. Game parameters used for the ten experimental treatments.** In our two experiments, individuals either coincide in all dimensions (full equality), they differ in single dimension (endowment inequality and productivity inequality), or they differ in two dimensions (aligned inequality and misaligned inequality).

## 2. Behavioral experiment of the two-player public goods games

In this section, we present a series of experiments based on the linear game and the threshold game. The experiment for the threshold game is conducted following the setup established by Wang et al. (1). Unlike the original study that examined scenarios where players differ in either endowment or productivity, our work explores situations where players vary in both dimensions simultaneously. We incorporate behavioral data from three specific treatments in Wang et al. (1), namely full equality, strong endowment inequality, and strong productivity inequality. This data serves as a benchmark for comparison against two new treatments: aligned inequality and misaligned inequality. The experimental setup of the linear game is similar to the experiment in Hauser et al. (2), but we change the players' initial endowments to match those in the threshold game.

**A. Description of the experimental design.** In the beginning of the experiment, participants are randomly allocated to one of the five treatments in the linear game or one of the two remaining treatments in the threshold game (see **Table S1**). Each treatment consists of two sessions. At the beginning of the first session, participants are randomly matched in pairs, with roles as player 1 or player 2 assigned randomly within each pair. In the second session, participants are re-matched and play the same game as in the first session, but with roles swapped. Those who were player 1 in the first session will now be player 2, and vice versa. These roles are maintained throughout the whole session of interaction with the same co-player. The treatment conditions are common knowledge within each treatment.

Each round of the game unfolds as outlined in Section 1 (participants receive their endowment, make their contribution decision, and receive the respective payoff). After each round, both participants are informed about the outcome. They learn each participant's absolute contribution, the group collective contribution, the return from the public good, and the resulting payoffs. The total number of rounds depends on the public goods game. For the linear game, participants are informed that they will engage in at least 20 rounds. Afterwards, there is a 50% continuation probability after each subsequent round. This experimental setup is designed to mitigate end-game effects, following the approach by Hauser et al. (2). The specific durations were sampled beforehand and applied to all groups. Specifically, the experiment for the linear game comprises 22 rounds in the first session and 21 in the second. In the experiment for the threshold game, both sessions last for 20 rounds, to be compatible with the study by Wang et al. (1).

Following the two sessions, participants are asked to complete a survey. For the linear game, the first question asks participants about their preferred contribution profile $(c_1, c_2)$. The second question requires participants to specify their contributions in response to all possible contributions of their co-player. To ensure thoughtful responses, participants are informed that two answers from the second question would be randomly selected to compute their payoffs from this part. For the threshold game, the first question asks participants' views on a fair contribution profile $(c_1, c_2)$. The second question requires participants to specify the minimum contribution they expect from their co-player to reach the threshold. Instructions and survey details for the two games are translated and included in Section 5B and 5C, respectively. The total payoff for each participant from the experiment includes their cumulative payoffs from both sessions and the survey. Note that only participants in the linear game receive earnings from the survey.

For the statistical analysis, we only use data from the first 20 rounds of each session. In the following, we refer to the first 20 rounds of the initial session as *Session 1* and the first 20 rounds of the subsequent session as *Session 2*.

**B. Description of the experimental procedures.** The threshold game experiment (AI and MI treatments) was conducted on September 28th and October 3rd, 2022, in the computer lab at Beijing Normal University. A total of 214 undergraduate students participated (104 in AI and 110 in MI). Participants who had previously taken part in the experiment by Wang et al. (1) were excluded. In addition, the number of participants in the FE, EI, and PI treatments (in Wang et al. (1)) are 110, 118, and 112, respectively. The linear game experiment was conducted between May and June 2023 in the same computer lab. There were 550 participants from Beijing Normal University, divided into five treatments: FE (114), EI (110), PI (110), AI (106), and MI (110). Participants from previous threshold game experiments, including those by Wang et al. (1) and the new treatments mentioned earlier, were not eligible for this experiment.

Before the experiment, participants in the same treatment receive the same instructions (in Chinese). An experimenter then explains the rules through a detailed presentation, followed by a series of comprehension questions. This procedure aims to ensure that all participants understand the game rules (details are provided in the Section 5B and 5C). Participants then engage in the game through an online platform that is created using o-Tree (version 5.3). Interactions are anonymous and conducted via computers, preventing any communication between participants. Schematic diagrams of the participants' screens are shown in the Section 5B and 5C.

The total payoff earned from the experiment is converted to Chinese Yuan at a ratio of 100:3. This amount plus a show-up fee constitute the final income of each participant. The show-up fee is 25 Chinese Yuan in the threshold game and 20 Chinese Yuan in the linear game. On average, participants in the linear game earned 64.07 Yuan (∼8.94 EUR), and those in the threshold game earned 61.01 Yuan (∼8.52 EUR).

**C. Data analysis.** For our statistical analysis, we use two-sided Mann-Whitney-Wilcoxon tests for comparisons between sessions or treatments and two-sided Wilcoxon signed-rank tests for within-group comparisons between players. These statistical tests are performed with Stata SE 13. We report the original $p$-values but adjust the significance level for multiple testing. The adjusted significance level is the original significance level divided by the number of conducted tests.

In the main text, we report the aggregated outcomes, by interpreting each session of the game as an independent observation. Further below, we provide a comparison of results between sessions and report comparisons between treatments per session.

***Linear public goods game.*** In **Table S2**, the sample sizes are 114, 110, 110, 106 and 110 (i.e., number of participants acting as player 1 (or 2) in the two sessions) in the five treatments. Significance levels are adjusted for 10 simultaneous tests (5 treatments with 2 tests each). In **Table S3**, the sample sizes are 114, 110, 110, 106 and 110 (i.e., number of groups in the two sessions) in the five treatments. Again, significance levels are adjusted for 10 simultaneous tests (5 treatments with 2 tests each). When we compare the players' contributions during the first five rounds versus with their contributions during the last five rounds, we use the Wilcoxon signed-rank test with the same sample sizes, adjusting significance levels for 5 simultaneous tests. In **Table S4**, the sample sizes are 114, 110, 110, 106 and 110 (i.e., number of groups in the two sessions) in the five treatments, and significance levels are adjusted for 20 tests (pairwise comparisons across 5 treatments with 2 tests per comparison). In **Table S5**, the samples are equally split into Session 1 and Session 2, but significance levels are also adjusted for 20 tests. In both **Table S6** and **Table S7**, the sample sizes are 228, 220, 220, 212, 220 (i.e., number of participants in the two sessions) for each of the five treatments, and the significance levels are adjusted for 10 simultaneous tests. Finally, for the payoff comparison in **Table S8**, the sample sizes are 228, 220, 220, 212, 220 (i.e., number of participants in the two sessions) for each of the five treatments, with significance levels adjusted for 5 simultaneous tests. For the Gini coefficient comparison in **Table S8**, the sample size are the sample sizes are 114, 110, 110, 106 and 110 (i.e., number of groups in the two sessions) in the five treatments.

***Threshold public goods game.*** In **Table S9**, the sample sizes are 110, 118, 112, 104 and 110 (i.e., number of participants acting as player 1 (or 2) in the two sessions) for each of the five treatments. Significance levels are adjusted for 10 simultaneous tests (5 treatments with 2 tests each). In **Table S10**, the sample sizes are 110, 118, 112, 104 and 110 (i.e., number of groups in the two sessions) in the five treatments. Again, significance levels are adjusted for 10 simultaneous tests (5 treatments with 2 tests each). In **Table S11**, the sample sizes are 110, 118, 112, 104 and 110 (i.e., number of groups in the two sessions) in the five treatments. Significance levels are adjusted for 20 tests (pairwise comparisons across 5 treatments with 2 tests per comparison). In **Table S12**, samples are equally split into Session 1 and Session 2, but significance levels are also adjusted for 20 tests. Finally, in **Table S13**, the sample sizes are 220, 236, 224, 208 and 220 (i.e., number of participants in the two sessions) in the five treatments, and significance levels are adjusted for 10 simultaneous tests. Finally, for the payoff comparison in **Table S14**, the sample sizes are 220, 236, 224, 208, 220 (i.e., number of participants in the two sessions) for each of the five treatments, with significance levels adjusted for 5 simultaneous tests. For the Gini coefficient comparison in **Table S14**, the sample size are the sample sizes are 110, 118, 112, 104 and 110 (i.e., number of groups in the two sessions) in the five treatments.

**D. Experimental results of the linear game.** In this section, we first analyze the players' contributions and their generated surplus. After that, we provide an analysis of the individual contribution patterns, conditional contributions, reciprocal behaviors, and payoff comparisons.

***Differences between sessions.*** In **Fig. S1**, we present experimental results across both sessions, highlighting qualitative similarities. In terms of players' absolute contributions, there is no significant difference between Session 1 and Session 2, except for a significant increase in average contributions during Session 2 in the productivity inequality treatment, see **Table S2**. As a consequence, both the group relative contributions and overall surplus in Session 2 are higher than those in Session 1 under productivity inequality, see **Table S3**.

| Linear PGG | Session 1 | | Session 2 | | Session 1 + Session 2 | | Session 1 vs. Session 2 $p$-values | |
|---|---|---|---|---|---|---|---|---|
| | Player 1 | Player 2 | Player 1 | Player 2 | Player 1 | Player 2 | Player 1 | Player 2 |
| FE | 16.31 | 16.51 | 18.44 | 18.40 | 17.38 | 17.45 | 0.0697 | 0.0334 |
| EI | 19.45 | 8.75 | 20.88 | 9.45 | 20.16 | 9.10 | 0.4351 | 0.1768 |
| PI | 14.35 | 14.29 | 18.35 | 18.13 | 16.35 | 16.21 | 0.0044* | 0.0054 |
| AI | 23.16 | 8.78 | 25.76 | 9.62 | 24.46 | 9.20 | 0.0829 | 0.2998 |
| MI | 16.09 | 8.02 | 19.39 | 8.79 | 17.74 | 8.40 | 0.1462 | 0.3336 |

**Table S2. Linear PGG: Contributions in Session 1 and Session 2.** Values in columns 2-7 are the average absolute contributions of the participants in the roles of player 1 and player 2, respectively. Values in columns 8-9 are the original $p$-values of two-sided Mann-Whitney-Wilcoxon tests. This analysis is based on each individual's average absolute contribution. To account for 10 simultaneous comparisons, the Bonferroni-corrected significance level becomes $0.05/10 = 0.005$ (1 asterisk).

| Linear PGG | Group relative contributions | | | Group overall surplus | | |
|---|---|---|---|---|---|---|
| | Session 1 | Session 2 | Session 1 vs. Session 2 $p$-values | Session 1 | Session 2 | Session 1 vs. Session 2 $p$-values |
| FE | 68.4% | 76.8% | 0.0314 | 41.0% | 46.1% | 0.0314 |
| EI | 58.7% | 63.2% | 0.3992 | 35.2% | 37.9% | 0.3992 |
| PI | 59.7% | 76.0% | 0.0048* | 35.8% | 45.7% | 0.0037* |
| AI | 66.5% | 73.7% | 0.1010 | 48.9% | 54.3% | 0.0964 |
| MI | 50.2% | 58.7% | 0.1766 | 25.1% | 28.6% | 0.1834 |

**Table S3. Linear PGG: Group relative contributions and overall surplus in Session 1 and Session 2.** Values in columns 2-3 and 5-6 are the average group relative contributions and the surplus in Session 1 and Session 2. Values in columns 4 and 7 are the original $p$-values of two-sided Mann-Whitney-Wilcoxon tests. The analysis is based on the relative contribution and the surplus of each group, averaged over all 20 rounds. To account for 10 simultaneous comparisons, the Bonferroni-corrected significance level becomes $0.05/10 = 0.005$ (1 asterisk).

***Group relative contributions and overall surplus.*** In the main text, we reported the aggregated outcomes, by interpreting each session of the game as an independent observation. Here, we first examine changes in group relative contributions over rounds (**Fig. 3B**). To this end, we compare each group's average relative contribution in the first five rounds with that in the last five rounds. Contributions are generally stable across treatments, with a significant increase observed under productivity inequality ($p = 0.0018$**, after Bonferroni correction) and aligned inequality ($p < 0.0001$***). The $p$-values for the other treatments are: full equality ($p = 0.4441$), endowment inequality ($p = 0.1234$), and misaligned inequality ($p = 0.4196$).

We then assess differences across the five treatments in both group relative contributions and overall surplus (**Table S4**). The full equality treatment exhibits the highest level of group relative contributions among all treatments, closely followed by the aligned inequality treatment. In contrast, the misaligned inequality treatment shows the lowest level of group relative contributions. With respect to the surplus, the aligned inequality treatment generates a significantly higher surplus than the other four treatments. Conversely, the misaligned inequality treatment yields a significantly lower surplus than all other treatments. Thus, the effects of double-dimensional inequality on cooperation depends on whether or not the larger endowment is allocated to the player with larger productivity. On average, aligned inequality increases a group's surplus, whereas misaligned inequality reduces it.

The results of these pairwise comparisons between treatments are robust in Session 1 and Session 2, where the misaligned inequality treatment has the lowest group relative contributions and overall surplus in both sessions (see **Fig. S1A,C,G,I** and **Table S5**).

***Comparing the contributions of the two players.*** In **Fig. 3** of the main text, we also show the absolute contributions and relative contributions for each treatment, separately for the two players. In **Table S6**, we compare absolute and relative contributions for all five treatments. When players have the same initial endowment, there are no significant differences in their contributions, either absolute or relative. However, when initial endowments differ, players with higher endowments make significantly larger contributions in absolute terms but contribute less in relative terms, compared to the low-endowment players.

***Contribution patterns.*** For the previous analysis, we averaged contributions across all groups that participated in a treatment. To provide a more detailed picture on the level of each individual group, **Fig. S2** illustrates the distribution of players' contributions $(c_1, c_2)$. This figure presents snapshots for four different time points: rounds 1, 5, 10, and 20 (**Fig. S2A-D**). In addition, it shows the aggregate distribution across all rounds (**Fig. S2E**). In both cases, panels aggregate the data from Session 1 and 2.

In the first round, the contribution patterns in all treatments are scattered (**Fig. S2A**). Full cooperation $(e_1, e_2)$ becomes more concentrated as the game progresses, indicating increasing cooperation among players. Over the 20 rounds, the most abundant contribution pattern across all treatments is full cooperation. In the aligned inequality treatment, nearly 50% of groups achieve full cooperation at a pace comparable to those in the treatments with full equality and productivity inequality.

| Linear PGG | Group relative contributions | FE | EI | PI | AI | MI | Group overall surplus |
|---|---|---|---|---|---|---|---|
| FE | 72.6% | | 0.0021* | 0.3548 | 0.0001** | <0.0001** | 43.5% |
| EI | 61.0% | 0.0021* | | 0.0518 | <0.0001** | 0.0001** | 36.6% |
| PI | 67.8% | 0.3145 | 0.0603 | | <0.0001** | <0.0001** | 40.8% |
| AI | 70.1% | 0.5492 | 0.0111 | 0.6591 | | <0.0001** | 51.6% |
| MI | 54.5% | <0.0001** | 0.1140 | 0.0013* | 0.0002** | | 26.8% |

**Table S4. Linear PGG: Group relative contributions and overall surplus across the five treatments.** Values in columns 2 and 8 are the average group relative contributions and the generated surplus, respectively. Values in columns 3-7 are the original $p$-values of two-sided Mann-Whitney-Wilcoxon tests. Here, orange cells contain the original $p$-values for comparisons with respect to differences in the group relative contributions between two treatments. Blue cells contain $p$-values for the respective comparisons with respect to the generated surplus. The analysis is based on the average relative contributions and the generated surplus at the group level. To account for 20 simultaneous comparisons, the Bonferroni-corrected significance levels become $0.05/20 = 0.0025$ (1 asterisk) and $0.01/20 = 0.0005$ (2 asterisks).

| | Session 1 | | | | | | Session 2 | | | | |
|---|---|---|---|---|---|---|---|---|---|---|---|
| | FE | EI | PI | AI | MI | | FE | EI | PI | AI | MI |
| FE | | 0.0887 | 0.2033 | 0.0144 | <0.0001** | FE | | 0.0096 | 0.8659 | 0.0018* | <0.0001** |
| EI | 0.0887 | | 0.7994 | 0.0008* | 0.0028 | EI | 0.0096 | | 0.0133 | <0.0001** | 0.0073 |
| PI | 0.2023 | 0.7902 | | 0.0011* | 0.0036 | PI | 0.7007 | 0.0180 | | 0.0016* | <0.0001** |
| AI | 0.7040 | 0.1539 | 0.3376 | | <0.0001** | AI | 0.6363 | 0.0316 | 0.9216 | | <0.0001** |
| MI | 0.0033 | 0.1296 | 0.1208 | 0.0099 | | MI | 0.0008* | 0.4530 | 0.0020* | 0.0073 | |

**Table S5. Linear PGG: Group relative contributions and overall surplus in Session 1 and Session 2.** The table presents the same kind of data as **Table S4**, but separately for Session 1 and Session 2. To account for 20 simultaneous comparisons, the Bonferroni-corrected significance levels become $0.05/20 = 0.0025$ (1 asterisk) and $0.01/20 = 0.0005$ (2 asterisks).

However, the misaligned inequality treatment exhibits the lowest proportion of full cooperation at all observed time points. According to the post-game survey, the pattern with full cooperation is preferred by participants, see **Fig. S3**.

***Conditional behaviors.*** **Fig. S4** shows the reactions of the two players to their co-player's previous contributions. **Fig. S4A** illustrates player 1's conditional behavior $(c_2(t-1), c_1(t))$, while **Fig. S4B** illustrates player 2's conditional behavior $(c_1(t-1), c_2(t))$, where $t$ refers to the round number, ranging from 2 to 20. Under full equality and productivity inequality, conditional contribution profiles tend to cluster around the line representing equal absolute or relative contributions. In the other three treatments with unequal endowments, most conditional contributions profiles are between the lines representing equal absolute contributions and equal relative contributions. The gray lines in **Fig. S4** are regression lines for conditional behaviors, excluding the defective reaction $(0, 0)$. The regression results are detailed in **Table S25**. Regression results including all conditional behaviors (including the defective outcome) are reported in **Table S26**. The results are robust. We interpret the positive slopes as an indication of direct reciprocity among players.

***Reciprocal behaviors across the five treatments.*** **Fig. S5** compares reciprocal behaviors between player 1 and player 2. To assess the players' reciprocal behavior, we calculate how often they match or exceed their co-player's previous contribution. For example, calculating player 1's frequency of reciprocal behavior based on the players' absolute contributions involves determining how often $c_1(t) \geq c_2(t-1)$ holds across the last 19 rounds. According to **Fig. S5A,C**, reciprocal behaviors are similarly abundant across the two players under full equality and productivity inequality. In the other three treatments, approximately 90% of high-endowment players act reciprocally with respect to absolute contributions, while around 90% of low-endowment players exhibit reciprocal behaviors when considering relative contributions (see **Table S7**).

Additionally, we illustrate the fraction of rounds where participants exactly match their co-player's previous contribution (see **Fig. S5B,D** and **Table S7**). Both players show similar results because this reciprocal behavior occurs more often when they consistently either fully contribute or contribute nothing at all.

***Payoffs and Gini coefficients.*** In **Fig. 3** of the main text, we show the Gini coefficients for each treatment. In the treatments with full equality and productivity inequality, the before-game Gini coefficient is 0. For the other three treatments with unequal endowments, the before-game Gini coefficient is 0.5. In **Table S8**, we compare the after-game Gini coefficients, calculated as $|\pi_1 - \pi_2|/(\pi_1 + \pi_2)$. Aligned inequality significantly reduces the Gini coefficient compared to the endowment inequality treatment and the misaligned inequality treatment. We also provide the Gini coefficient comparisons for each session separately (see **Fig. S1D,J**). In addition, we compare the payoffs of the two players in **Table S8**. When players have the same initial endowment, there are no significant differences in their payoffs. However, when initial endowments differ, high-endowment players obtain significantly higher payoffs after the game, see also **Fig. S6A**.

| Linear PGG | Absolute contributions | | | Relative contributions | | |
|---|---|---|---|---|---|---|
| | Player 1 | Player 2 | Player 1 vs. Player 2 $p$-values | Player 1 | Player 2 | Player 1 vs. Player 2 $p$-values |
| FE | 17.38 | 17.45 | 0.8473 | 72.4% | 72.7% | 0.8451 |
| EI | 20.16 | 9.10 | <0.0001*** | 56.0% | 75.8% | <0.0001*** |
| PI | 16.35 | 16.21 | 0.2307 | 68.1% | 67.6% | 0.2221 |
| AI | 24.46 | 9.20 | <0.0001*** | 67.9% | 76.6% | 0.0001** |
| MI | 17.74 | 8.40 | <0.0001*** | 49.3% | 70.0% | <0.0001*** |

**Table S6. Comparison of absolute and relative contributions of the two players in the linear PGG.** Values in columns 2-3 contain the average absolute contributions of the two players, whereas columns 5-6 contain their relative contributions (with results for Session 1 and Session 2 being aggregated). Values in columns 4 and 7 are the original $p$-values of paired Wilcoxon signed-rank tests. The analysis is based on the contributions at the individual level (averaged over 20 rounds during which the given two players interact). To account for 10 simultaneous comparisons, the Bonferroni-corrected significance levels become $0.01/10 = 0.001$ (2 asterisks) and $0.001/10 = 0.0001$ (3 asterisks).

| Linear PGG | | Matched or Exceed | | | Matched | | |
|---|---|---|---|---|---|---|---|
| | | Player 1 | Player 2 | Player 1 vs. Player 2 $p$-values | Player 1 | Player 2 | Player 1 vs. Player 2 $p$-values |
| Computed based on absolute contribution | FE | 84.4% | 86.4% | 0.5364 | 68.3% | 69.0% | 0.3789 |
| | EI | 89.6% | 29.2% | <0.0001*** | 19.5% | 18.1% | 0.4631 |
| | PI | 82.3% | 81.9% | 0.5964 | 60.0% | 59.6% | 0.1537 |
| | AI | 92.3% | 17.8% | <0.0001*** | 9.7% | 8.9% | 0.5358 |
| | MI | 86.0% | 33.0% | <0.0001*** | 19.0% | 17.6% | 0.1840 |
| Computed based on relative contribution | FE | 84.4% | 86.4% | 0.5364 | 68.3% | 69.0% | 0.3789 |
| | EI | 44.6% | 89.6% | <0.0001*** | 33.8% | 33.9% | 0.6978 |
| | PI | 82.3% | 81.9% | 0.5964 | 60.0% | 59.6% | 0.1537 |
| | AI | 66.9% | 85.1% | 0.0006** | 50.6% | 49.4% | 0.1118 |
| | MI | 38.9% | 88.5% | <0.0001*** | 29.1% | 28.7% | 0.3540 |

**Table S7. Comparison of reciprocal behaviors of the two players in the linear PGG.** Values in columns 3-4 represent the fractions of rounds in which participants match or exceed their co-player's contribution from the previous round, while values in columns 6-7 are fractions of rounds in which participants exactly match their co-player's contribution from the previous round (with results for Session 1 and Session 2 being aggregated). Values in columns 5 and 8 are the original $p$-values of paired Wilcoxon signed-rank tests. The analysis is based on the frequency of reciprocity behaviors at the individual level. To account for 10 simultaneous comparisons, the Bonferroni-corrected significance levels are $0.01/10 = 0.001$ (2 asterisks) and $0.001/10 = 0.0001$ (3 asterisks).

**E. Experimental results of the threshold game.** In the following, we first analyze the group success rate and overall surplus. After that, we provide an analysis of the players' contribution patterns and conditional behaviors. Additionally, we compare the players' payoff and we analyze why some groups failed to reach the threshold. For comparisons between treatments, we summarize the statistical outcomes of the full equality, strong endowment inequality, and strong productivity inequality treatments from Wang et al. (1) in tables. The statistical results for the aligned inequality treatment and misaligned inequality treatment are derived by analyzing the data of our current experiment.

***Differences between sessions.*** Again, in the main text we reported aggregated statistical results, for which we combine the data from the two sessions. In **Fig.** S7, we show results for each session separately. With regard to the players' average absolute contributions, no significant differences are observed between Session 1 and Session 2, see **Table** S9. Similarly, there are no significant differences when we compare the two sessions in terms of their success rates (how often group collective contributions match or exceed the threshold) and their surplus (by how much the total payoffs of the players exceed their initial endowments), see **Table** S10.

***Group success rates and overall surplus.*** In the main text, we aggregate Session 1 and Session 2 for analysis. In **Table** S11, we compare the effects of the five treatments on group success rates and overall surplus. We find that success rates and overall surplus are significantly higher in those treatments without endowment asymmetry. Furthermore, when productivity asymmetry is introduced alongside endowment inequality in the treatments, it does not lead to a significant change in success rates and overall surplus. That is, as opposed to the linear game, there is no advantage to aligned inequality in the threshold game. The results of these pairwise comparisons between treatments are robust in Session 1 and Session 2, see **Table** S12.

| Linear PGG | Payoffs | | | Gini coefficients | |
|---|---|---|---|---|---|
| | Player 1 | Player 2 | Player 1 vs. Player 2 $p$-values | | |
| FE | 34.49 | 34.41 | 0.8462 | 0.04 | Treatment, p-values: |
| EI | 39.25 | 26.31 | <0.0001*** | 0.23 | FE vs. PI, 0.9211 |
| PI | 33.72 | 33.86 | 0.2366 | 0.05 | EI vs. AI, 0.0029* |
| AI | 40.76 | 32.02 | <0.0001*** | 0.17 | EI vs. MI, 0.1069 |
| MI | 37.77 | 23.11 | <0.0001*** | 0.27 | AI vs. MI, <0.0001*** |

**Table S8. Payoffs and after-game Gini coefficients for different treatments in the linear PGG.** Values in columns 2 and 3 contain the average payoffs of the two players, respectively. Values in column 4 are the original $p$-values of paired Wilcoxon signed-rank tests. The analysis is based on the payoffs at the individual level (averaged over 20 rounds during which the given two players interact). To account for 5 simultaneous comparisons, the Bonferroni-corrected significance level becomes $0.001/5 = 0.0002$ (3 asterisks). Values in column 5 are the average after-game Gini coefficients of groups, averaged over 20 rounds. Column 6 contains the original $p$-values for comparisons with respect to the after-game Gini coefficients between two treatments. To account for 3 simultaneous comparisons across treatments with unequal endowments, the Bonferroni-corrected significance levels become $0.05/3 \approx 0.0167$ (1 asterisk) and $0.001/3 \approx 0.0003$ (3 asterisks).

***Comparing the contributions of the two players.*** In **Table S13** and **Fig. S7E,F,K,L**, we compare both absolute and relative contributions between players. In the aligned inequality treatment, the high-endowment players contribute more than the low-endowment players in both absolute and relative terms. In the misaligned treatment, while high-endowment players still contribute more in absolute terms, their relative contributions are lower than the contributions of the low-endowment players (see also **Fig. 3K,L** in the main text).

***Contribution patterns.*** **Fig. S8** illustrates the distribution of group contributions $(c_1, c_2)$ in the threshold game. All panels aggregate the data from Session 1 and 2. In the first round, the contribution patterns in both the aligned inequality and misaligned inequality treatments are scattered, resembling the pattern observed in the endowment inequality treatment. Conversely, in the full equality and productivity inequality treatments, players tend to quickly coordinate on contributing half of their endowments. Moreover, throughout the game, the outcome $(0, 0)$ is more observable in treatments with unequal endowments, highlighting increased coordination challenges and a higher likelihood of mutual defection among players with different endowments.

The most abundant contribution pattern gradually becomes more concentrated as the game progresses, indicating increasing coordination among players (see **Fig. S8A-D**). Among treatments with unequal endowments, the most abundant overall contribution pattern is $(18, 6)$, both players contributing half of their endowments (see **Fig. S8E**).

According to the post-game survey, approximately 40% of participants consider the outcome with equal relative contributions (ERC) to be the most fair in the aligned inequality treatment and misaligned inequality treatment (see **Fig. S9**). The proportion of participants who consider ERC to be fair is the highest across all five treatments. Players' views on the fair contribution pattern show some similarity to the realized outcomes observed in the experiment.

***Conditional behaviors.*** Similar to **Fig. S4** of the linear game, **Fig. S10** presents conditional behaviors observed in the threshold game. In the three treatments with unequal endowments, contributions of players are negatively correlated to the co-player's contributions in the previous round. Specifically, most of the response profiles are on the threshold line $p_i c_i(t) + p_j c_j(t-1) = \theta$, where $c_i(t)$ is the contribution of the focal player at round $t$ and $c_j(t-1)$ is the contribution of the co-player at round $t-1$. The gray lines are regression lines for conditional behavior without defective reaction $(0, 0)$. The regression results are detailed in **Table S25**. The negative slope in the three treatments with unequal endowments indicates coordination between players. In the full equality and productivity inequality treatments, the majority of contribution behaviors were concentrated at $(12, 12)$, resulting in a poor fit of the regression model. Additionally, we report the regression results including all conditional behaviors in **Table S26**. Due to the defective reaction at $(0, 0)$, the regression equations for all treatments except the misaligned inequality treatment exhibit positive slopes, and the fit of the regression models becomes worse.

***Payoffs and Gini coefficients.*** The before-game Gini coefficients are the same as in the linear game (0 under full equality and productivity inequality, and 0.5 in the other three treatments). In **Table S14**, we compare the after-game Gini coefficients across treatments. We find that aligned inequality leads to the largest reduction in the Gini coefficient, as in the linear game. We also provide the Gini coefficient comparison for each session separately (see **Fig. S7D,J**). In addition, we compare the payoffs of the two players in **Table S14**. Similar to the linear game, when players have the same initial endowment, there are no significant differences in their payoffs. However, when initial endowments differ, high-endowment players obtain significantly higher payoffs after the game (see also **Fig. S6B**).

***Failure groups.*** The previous results in **Fig. S9** suggest that most individuals consider the outcome with equal relative contributions as a natural baseline. In the following, we use this baseline to analyze why some groups failed to reach the threshold. Following the method in Wang et al. (1), we categorize failing groups into three classes: 1) those where only player 1 contributes less than half their endowment; 2) those where only player 2 contributes less than half their endowment; and 3) those where both players contribute less than half their endowments.

| Threshold PGG | Session 1 | | Session 2 | | Session 1 + Session 2 | | Session 1 vs. Session 2 $p$-values | |
|---|---|---|---|---|---|---|---|---|
| | Player 1 | Player 2 | Player 1 | Player 2 | Player 1 | Player 2 | Player 1 | Player 2 |
| FE | 11.76 | 11.72 | 11.63 | 11.66 | 11.70 | 11.69 | 0.5146 | 0.4310 |
| EI | 13.75 | 7.18 | 14.24 | 7.38 | 14.00 | 7.28 | 0.4721 | 0.9828 |
| PI | 11.72 | 11.50 | 11.94 | 11.64 | 11.83 | 11.57 | 0.8360 | 0.5368 |
| AI | 16.87 | 4.98 | 16.86 | 4.50 | 16.87 | 4.74 | 0.5513 | 0.5781 |
| MI | 14.86 | 6.09 | 14.98 | 6.24 | 14.92 | 6.17 | 0.7854 | 0.9070 |

**Table S9. Threshold PGG: Contributions in Session 1 and Session 2.** Values in columns 2-7 are the average absolute contributions of the participants in the roles of player 1 and player 2, respectively. Values in columns 8-9 are the original $p$-values of two-sided Mann-Whitney-Wilcoxon tests. There are no significant differences even after Bonferroni correction. This analysis is based on each individual's average absolute contribution.

| Threshold PGG | Group success rates | | | Group overall surplus | | |
|---|---|---|---|---|---|---|
| | Session 1 | Session 2 | Session 1 vs. Session 2 $p$-values | Session 1 | Session 2 | Session 1 vs. Session 2 $p$-values |
| FE | 94.5% | 95.3% | 0.3580 | 29.9% | 30.9% | 0.4547 |
| EI | 72.4% | 82.3% | 0.0258 | 16.7% | 23.5% | 0.0015* |
| PI | 91.2% | 97.3% | 0.0005** | 27.6% | 32.0% | 0.0002** |
| AI | 82.3% | 83.5% | 0.5136 | 23.1% | 25.1% | 0.1794 |
| MI | 78.2% | 82.3% | 0.4083 | 21.5% | 24.4% | 0.2831 |

**Table S10. Threshold PGG: Group success rates and overall surplus in Session 1 and Session 2.** Values in columns 2-3 and 5-6 are the average success rates and the surplus in Session 1 and Session 2. Values in columns 4 and 5 are the original $p$-values of two-sided Mann-Whitney-Wilcoxon tests. The analysis is based on the success rates and the surplus of each group, averaged over all 20 rounds. To account for 10 simultaneous comparisons, the Bonferroni-corrected significance levels become $0.05/10 = 0.005$ (1 asterisk) and $0.01/10 = 0.001$ (2 asterisks).

**Fig. S11** depicts the absolute numbers and proportions of these three classes across the five treatments. In the treatments with double-dimensional inequality, approximately 80% of failure groups involve player 1 contributing less than half of their endowment (theses failures either belong to the first or third class). Furthermore, failure groups in the aligned inequality treatment show a smaller percentage of failures due to player 1's under-contribution compared to the endowment inequality treatment. This supports the finding that players with higher endowment and productivity are more likely to contribute a larger share in coordination, unlike the scenario where endowment is the only difference between players.

## 3. Individual-based simulations

In the following, we explain the setup of our individual-based simulations in more detail.

**A. Reactive strategies.** In this study, we explore the dynamics among players with reactive strategies. According to these strategies, a player's action only depends on their co-player's contribution in the previous round (7–11). Formally, a reactive strategy for player 1 is defined as a vector $R^1 = (c^1; c^1_0, c^1_1, \dots, c^1_i, \dots, c^1_{e_2})$, where $c^1$ is player 1's initial contribution and $c^1_i$ denotes player 1's contribution in response to co-player's contribution $i$ in the previous round. Player 2's reactive strategy is similarly defined as $R^2 = (c^2; c^2_0, c^2_1, \dots, c^2_i, \dots, c^2_{e_1})$. A random reactive strategy is one in which each entry of $R^i$ is independently drawn from the set of possible contributions $S_i$. Since strategies are deterministic, the vectors $R^1$ and $R^2$ uniquely determine the players' contributions in all rounds.

**B. Utility function.** We consider utility functions that allow for social preferences. According to our experimental observations, the most common contribution patterns reflect a preference for equal relative contributions (ERC) or equal absolute contributions (EAC). In contrast, players rarely act to equalize final payoffs, unless such outcomes are aligned with EAC or ERC. Motivated by these findings, we incorporate preferences for EAC and ERC into the utility function, rather than using the well-known Fehr & Schmidt representation (12). Let $c_1 \in S_1$ and $c_2 \in S_2$ denote the contributions of player 1 and player 2, respectively. The utility of player $i$ is defined as:

$$u_i(c_1, c_2) = \pi_i(c_1, c_2) - \beta \frac{|c_1 - c_2|}{\max\{e_i\}} - \gamma \left| \frac{c_1}{e_1} - \frac{c_2}{e_2} \right| . \qquad [5]$$

The first term on the right hand side represents the material payoff to player $i$. The second and third terms reflect disutility from deviations from EAC and ERC, respectively, with parameters $\beta$ and $\gamma$ indicating the strength of each preference. Players choose their strategies to maximize their own utility. Setting $\beta = \gamma = 0$ recovers the usual default model, in which players seek to maximize their material payoffs.

| Threshold PGG | Group success rates | FE | EI | PI | AI | MI | Group overall surplus |
|---|---|---|---|---|---|---|---|
| FE | 94.9% | | <0.0001** | 0.6807 | <0.0001** | <0.0001** | 30.4% |
| EI | 77.3% | <0.0001** | | <0.0001** | 0.0506 | 0.3397 | 20.1% |
| PI | 94.2% | 0.3807 | <0.0001** | | <0.0001** | <0.0001** | 29.8% |
| AI | 82.9% | <0.0001** | 0.2311 | 0.0006* | | 0.3355 | 24.1% |
| MI | 80.2% | <0.0001** | 0.9348 | <0.0001** | 0.2483 | | 22.9% |

**Table S11. Threshold PGG: Group success rates and overall surplus across the five treatments.** Values in culumns 2 and 8 are the success rates and the surplus, respectively. Values in columns 3-7 are the original $p$-values of two-sided Mann-Whitney-Wilcoxon tests. Here, green cells contain the original $p$-values for comparisons with respect to differences in the success rates between two treatments. Blue cells contain $p$-values for the respective comparisons with respect to the surplus. The analysis is based on the average success and the surplus at the group level. To account for 20 simultaneous comparisons, the Bonferroni-corrected significance levels become $0.05/20 = 0.0025$ (1 asterisk) and $0.01/20 = 0.0005$ (2 asterisks).

| | Session 1 | | | | | Session 2 | | | | | |
|---|---|---|---|---|---|---|---|---|---|---|---|
| | FE | EI | PI | AI | MI | | FE | EI | PI | AI | MI |
| FE | | <0.0001** | 0.0786 | <0.0001** | <0.0001** | FE | | <0.0001** | 0.1553 | 0.0017* | 0.0001** |
| EI | <0.0001** | | <0.0001** | 0.0356 | 0.1104 | EI | <0.0001** | | <0.0001** | 0.3861 | 0.8469 |
| PI | 0.0530 | <0.0001** | | 0.0074 | 0.0007* | PI | 0.2862 | <0.0001** | | 0.0002** | <0.0001** |
| AI | <0.0001** | 0.1195 | 0.0062 | | 0.6180 | AI | <0.0001** | 0.8714 | <0.0001** | | 0.4068 |
| MI | <0.0001** | 0.5133 | 0.0002** | 0.3715 | | MI | <0.0001** | 0.4555 | <0.0001** | 0.4874 | |

**Table S12. Threshold PGG: Group success rates and overall surplus in Session 1 and Session 2.** The table presents the same kind of data as **Table S11**, but separately for Session 1 and Session 2. To account for 20 simultaneous comparisons, the Bonferroni-corrected significance levels become $0.05/20 = 0.0025$ (1 asterisk) and $0.01/20 = 0.0005$ (2 asterisks).

**C. Introspection dynamics.** To model how players adapt their behavior over time, we adopt an introspection dynamics framework (2, 13, 14). In this setting, players 1 and 2 interact repeatedly over many time steps. Initially, both players are assigned random reactive strategies, as defined earlier. Then, in each time step of the learning process, the pair of players engage in 20 rounds of the game (as in our experiment). Afterward, one of the two players is randomly selected to potentially update their strategy. The selected player $i$ randomly draws an alternative reactive strategy $\tilde{R}^i$, and compares their realized average utility $u_i$ (over 20 rounds) to the hypothetical utility $\tilde{u}_i$ they would have obtained by using $\tilde{R}^i$ (assuming the co-player's reactive strategy remains unchanged). The player then switches to the alternative strategy $\tilde{R}^i$ with probability

$$\varphi(\tilde{u}_i, u_i) := \frac{1}{1+\exp(-s(\tilde{u}_i - u_i))}, \qquad [6]$$

where $s \geq 0$ is the selection strength, which governs how strongly utility differences influence strategy update. Larger values of $s$ increase the likelihood of switching to a strategy that yields higher utility.

When the update process is iterated many times, it induces a Markov process on the space of all strategy profiles $(R^1, R^2)$. For any finite selection strength $s$, this process has a unique stationary distribution, which is independent of the players' initial reactive strategies. However, the space of reactive strategies is too large to compute this stationary distribution exactly. Instead, we run agent-based simulations to explore the behavioral dynamics numerically. The corresponding code is available in our online repository (15).

**D. Simulation methods.** In each time step, the two players interact for 20 rounds using their reactive strategies. Each round, players compute their utilities as described in Eq. (5) each round, as well as their average utility across all 20 rounds. Then one of the two players is randomly selected to potentially update their strategy based on introspection dynamics, as described in Section 3C. Throughout, we assume both players use the same utility function. That is, they share identical values of $\beta$ and $\gamma$.

For each parameter combination, we run simulations of introspection dynamics for $10^7$ time steps. To capture the resulting dynamics, we record the players' contribution profiles $(c_1, c_2)$ over the 20 rounds of every time step. These contribution profiles are subsequently analyzed.Furthermore, to explore the role of repeated-game effects, we additionally run simulations for the one-shot version of the game. In this setting, players interact for only a single round per time step, while all other aspects of the model remain unchanged. As a result, only the initial contribution of a reactive strategy affects its utility. In this cases, reactive strategies become equivalent to unconditional strategies.

**E. Parameters estimation.** To investigate for which model parameters the simulated dynamics provides the best match to the observed experimental results, we systematically vary parameters and ran separate simulations for each game. We vary the

| Threshold PGG | Absolute contributions | | | Relative contributions | | |
|---|---|---|---|---|---|---|
| | Player 1 | Player 2 | Player 1 vs. Player 2 $p$-values | Player 1 | Player 2 | Player 1 vs. Player 2 $p$-values |
| FE | 11.70 | 11.69 | 0.9657 | 48.7% | 48.7% | 0.9753 |
| EI | 14.00 | 7.28 | <0.0001*** | 38.9% | 60.7% | <0.0001*** |
| PI | 11.83 | 11.57 | 0.0674 | 49.3% | 48.2% | 0.0676 |
| AI | 16.87 | 4.74 | <0.0001*** | 46.9% | 39.5% | 0.0033* |
| MI | 14.92 | 6.17 | <0.0001*** | 41.4% | 51.4% | 0.0009** |

**Table S13. Comparison of absolute and relative contributions of the two players in the threshold PGG.** Values in columns 2-3 contain the average absolute contributions of the two players, whereas columns 5-6 contain their relative contributions (with results for Session 1 and Session 2 being aggregated). Values in columns 4 and 7 are the original $p$-values of paired Wilcoxon signed-rank tests. The analysis is based on the average contributions at the individual level (averaged over all 20 rounds during which the given two players interact). To account for 10 simultaneous comparisons, the Bonferroni-corrected significance levels become $0.05/10 = 0.005$ (1 asterisk), $0.01/10 = 0.001$ (2 asterisks) and $0.001/10 = 0.0001$ (3 asterisks).

| Threshold PGG | Payoffs | | | Gini coefficient | |
|---|---|---|---|---|---|
| | Player 1 | Player 2 | Player 1 vs. Player $p$-values | | |
| FE | 31.28 | 31.29 | 0.9767 | 0.01 | Treatment, p-values: |
| EI | 37.47 | 20.18 | <0.0001*** | 0.32 | FE vs. PI, 0.4619 |
| PI | 31.02 | 31.28 | 0.0660 | 0.01 | EI vs. AI, <0.0001*** |
| AI | 35.71 | 23.84 | <0.0001*** | 0.22 | EI vs. MI, 0.0023** |
| MI | 37.13 | 21.88 | <0.0001*** | 0.27 | AI vs. MI, <0.0001*** |

**Table S14. Payoffs and after-game Gini coefficients for different treatments in the threshold PGG.** Values in columns 2 and 3 contain the average payoffs of the two players, respectively. Values in column 4 are the original $p$-values of paired Wilcoxon signed-rank tests. The analysis is based on the payoffs at the individual level (averaged over 20 rounds during which the given two players interact). To account for 5 simultaneous comparisons, the Bonferroni-corrected significance level becomes $0.001/5 = 0.0002$ (3 asterisks). Values in column 5 are the average after-game Gini coefficients of groups, averaged over 20 rounds. Column 6 contains the original $p$-values for comparisons with respect to the after-game Gini coefficients between two treatments. To account for 3 simultaneous comparisons across treatments with unequal endowments, the Bonferroni-corrected significance levels become $0.01/3 \approx 0.0033$ (2 asterisks) and $0.001/3 \approx 0.0003$ (3 asterisks).

preference strengths $\beta$ and $\gamma$ over $\beta \in \{0, 1, 2, \ldots, 30\}, \gamma \in \{0, 1, 2, \ldots, 100\}$. In addition, we consider three possible selection strengths, $s \in \{1, 10, 100\}$.

To measure the fit between our different simulations and the experimental data, we use an objective function based on the group overall surplus (GOS). First, we estimate optimal parameter values for each type of public goods game separately (i.e., we obtain one set of parameters to account for behavior in the linear games and another set of parameters to account for behavior in the threshold games). By doing so, we take into account that players may approach the two games with different sets of norms, or that the setups might trigger different sets of heuristics. The respective objective function $\Delta_{GOS}$ is given by

$$\Delta_{GOS} = \sqrt{\begin{array}{l} \left(GOS^{\text{FE}}_{exp} - GOS^{\text{FE}}_{sim}\right)^2 + \left(GOS^{\text{EI}}_{exp} - GOS^{\text{EI}}_{sim}\right)^2 + \left(GOS^{\text{PI}}_{exp} - GOS^{\text{PI}}_{sim}\right)^2 + \\ \left(GOS^{\text{AI}}_{exp} - GOS^{\text{AI}}_{sim}\right)^2 + \left(GOS^{\text{MI}}_{exp} - GOS^{\text{MI}}_{sim}\right)^2 \end{array}}. \quad [7]$$

Here, $GOS^k_{exp}$ refers to the average group overall surplus in treatment $k$ from the experiment, with $k \in \{\text{FE}, \text{EI}, \text{PI}, \text{AI}, \text{MI}\}$. It is computed by averaging the surplus across groups, where each group's overall surplus is the average over 20 rounds. By contrast, $GOS^k_{sim}$ denotes the average group overall surplus obtained from the simulation for treatment $k$. Since the simulation involves only one group repeatedly interacting over many time steps, $GOS^k_{sim}$ is computed by averaging the group overall surplus across all simulated rounds.

Beyond evaluating the fit within each type of public goods game separately, we further examine whether a single parameter combination can jointly capture behavioral outcomes across all ten treatments. To this end, we define a merged objective function as

$$\Delta_{MGOS} = \sqrt{\sum_{k \in L} \left(GOS^k_{exp} - GOS^k_{sim}\right)^2 + \sum_{k \in T} \left(GOS^k_{exp} - GOS^k_{sim}\right)^2}. \quad [8]$$

Here, $L$ and $T$ denote the sets of treatments $\{\text{FE}, \text{EI}, \text{PI}, \text{AI}, \text{MI}\}$ in the linear game and the threshold game, respectively.

**F. Numerical results.**

***Impact of individual preferences.*** Before reporting the optimal parameter values that best match the experimental results, we first present illustrative simulation results to provide an intuitive understanding of how individual preferences for equal absolute contribution (EAC) or equal relative contribution (ERC) influence behavior in the two games. **Fig. S12** and **Fig. S13** show simulation outcomes under selected values of $\beta$ (EAC preference) or $\gamma$ (ERC preference), compared to the baseline with no preference. While these figures reflect only a specific setting, they capture the typical qualitative effects observed across a broader range of parameters.

For the linear game, **Fig. S12** illustrates the distribution of action pairs throughout the simulation. In the full equality and productivity inequality treatments, as either $\beta$ or $\gamma$ increases, the distribution puts more weight on full cooperation. In the three treatments with unequal endowments, a strong ERC preference (large $\gamma$) clearly promotes full cooperation, while a strong EAC preference (large $\beta$) leads to outcomes that deviate from full cooperation.

In the threshold game (see **Fig. S13**), both preference types seem to facilitate successful coordination towards one of the cooperative one-shot equilibria (where total group contributions exactly match the threshold). Specifically, increasing $\beta$ leads to a clustering of actions near the intersection of the cooperative equilibria and the line associated with the EAC pattern. Similarly, increasing $\gamma$ results in actions near the intersection of cooperative equilibria and the line associated with the ERC pattern.

***Optimal parameters for predicting experimental results.*** **Fig. S14** shows the value of the objective function when the selection strength is fixed to $s = 1$. The best fit to the experimental data is achieved at the $(\gamma, \beta)$ marked by the white box.

Let us first discuss the results when we seek an optimal agreement between simulations and experimental data for each public goods game type separately. For the linear game, the best fit to the experimental results is achieved with $s = 1$ and $(\gamma, \beta) = (14, 0)$ (see **Fig. S14A**). Although the simulated action distributions show some minor differences from the experimental ones (**Fig. S19A** vs. **Fig. S2E**), the simulated group surplus closely matches the experimental results across all treatments (see **Fig. 3C**). This parameter set also successfully reproduces other behavioral outcomes observed in the experiment, including the group's relative contributions (**Fig. 3A**), after-game Gini coefficients (**Fig. 3D**), and average individual contributions (**Fig. 3E,F**). In addition, the simulation captures the conditional contribution patterns reported in the questionnaire, especially when the co-player's relative contribution exceeds 50% (**Fig. S15**).

For the threshold game, the best fit to the experimental results is obtained with $s = 1$ and $(\gamma, \beta) = (94, 18)$. The simulation results closely match the group overall surplus (**Fig. 3I**), and successfully reproduce other behavioral outcomes, including the group's relative contributions (**Fig. 3G**), after-game Gini coefficients (**Fig. 3J**), and average individual contributions (**Fig. 3K,L**). Across treatments, although the frequencies vary, the most abundant contribution pattern is accurately reproduced in the simulation (**Fig. S20A** vs. **Fig. S8E**). Moreover, the simulations capture the conditional contribution patterns most frequently observed in the experiment (**Fig. S16**).

These results highlight an interesting difference between the two games. In the linear game, a pure ERC preference (i.e., $\gamma > 0$, $\beta = 0$) is sufficient to reproduce the experimental outcomes across all treatments. By contrast, in the threshold game, incorporating both ERC and EAC preferences result in a strictly better fit (note that **Fig. S14**, there is a whole range of parameter values that perform almost equally well as the optimal parameter combination; however, all of those parameter value pairs satisfy $\beta > 0$). When only one type of preference is included in the simulation for the threshold game, the results diverge substantially from the experimental data, as indicated by markedly higher values of $\Delta_{GOS}$ (see **Fig. S17C,D**).

The differences between the two games also highlight the challenge of identifying a single parameter combination that fits both public goods game types simultaneously. When we optimize the objective function Eq. (8) across all ten treatments, the optimal shared parameter set is found at $(\gamma, \beta) = (20, 4)$ (see **Fig. S14C**). However, this parameter combination fails to reproduce some of the behavioral patterns in either game type, as shown by the substantial deviations from experimental benchmarks (**Fig. S18**).

***Repeated-game effects.*** Based on the optimal parameters obtained above, we further examine whether repeated interactions are essential for sustaining cooperation among players with social preferences. To this end, we simulate behavior in a one-shot setting, where only a single round is played per time step (as opposed to 20 rounds in the repeated setting). For the linear game, we find that the behavior of both players evolves towards mutual defection in all treatments (see **Fig. S19B**) This provides evidence that reciprocity is important to account for cooperation in these games. In the threshold game, some simulation runs converge to mutual defection, while others reach cooperative Nash equilibria (see **Fig. S20B**). Averaged over 1,000 iterations, the success rates for the five treatments are (58.95, 58.17, 63.50, 59.69, 59.62)%, which is lower than in the repeated game case. This indicates the inherent risk of coordination failure in one-shot settings and highlights the importance of repeated interaction in promoting cooperation.

## 4. Four-player public goods games

To assess the robustness of our previous findings with respect to changes in group size, we implemented an additional experiment on public goods games in groups of four. To gain some intuition, we first discuss such games formally.

### A. Model and Nash equilibrium analysis.

| | | | Full equality (FE) | Aligned inequality (AI) | Misaligned inequality (MI) |
|---|---|---|---|---|---|
| **Four-player linear PGG** | Endowments | $e_1 = e_2$ | 24 | 36 | 36 |
| | | $e_3 = e_4$ | 24 | 12 | 12 |
| | Productivities | $p_1 = p_2$ | 3.2 | 3.8 | 2.6 |
| | | $p_3 = p_4$ | 3.2 | 2.6 | 3.8 |
| **Four-player threshold PGG** | Endowments | $e_1 = e_2$ | 24 | 36 | 36 |
| | | $e_3 = e_4$ | 24 | 12 | 12 |
| | Productivities | $p_1 = p_2$ | 1 | 3 | 1 |
| | | $p_3 = p_4$ | 1 | 1 | 3 |
| | Rewards | $r_1 = r_2 = r_3 = r_4$ | 20 | 20 | 20 |
| | Threshold | $\theta$ | 48 | 120 | 72 |

**Table S15. Game parameters used for the four-player experiment.** In both public goods games, individuals either coincide in all dimensions (full equality), or they differ in two dimensions (aligned inequality and misaligned inequality).

***Description of four-player games.*** The structure of the four-player game follows the same rules described in Section 1. For the linear public goods game, the payoff for player $i$ ($i = 1, 2, 3, 4$) is:

$$\pi_i(\boldsymbol{c}) = e_i - c_i + \frac{1}{4}\sum_{k=1}^{4} p_k c_k, \qquad [9]$$

where $\boldsymbol{c} = (c_1, c_2, c_3, c_4)$ refers to the players' contributions, and each player's productivity satisfies $1 < p_i < 4$. For the threshold public goods game, the payoff is given by:

$$\pi_i(\boldsymbol{c}) = \begin{cases} e_i - c_i + r_i & \text{if} \quad \sum_{k=1}^{4} p_k c_k \geq \theta, \\ e_i - c_i & \text{otherwise.} \end{cases} . \qquad [10]$$

In our implementation of these games, we assume both players 1 and 2 choose their contributions $c_1$ and $c_2$ from strategy set $S_1$, while players 3 and 4 choose $c_3$ and $c_4$ from $S_2$. We focus on three treatments: full equality (FE), aligned inequality (AI), and misaligned inequality (MI). We focus on them because the treatments with aligned iand misaligned inequality represent the two inequality configurations of greatest interest to us, while full equality serves as a neutral benchmark. The experimental parameters for the six treatments (three in the linear game and three in the threshold game) are summarized in **Table S15**.

To ensure meaningful comparisons with the two-player setup, treatment parameters are slightly adjusted. In the linear game, we double each player's productivity so that their marginal per capita return remains unchanged. In the threshold game, the threshold is adjusted following the same logic as in the two-player setup: the group exactly meets the threshold if each player contributes half of their endowment.

***Nash equilibrium analysis.*** For the one-shot linear game, the unique Nash equilibrium is again for all players to contribute nothing. For the one-shot threshold game, multiple Nash equilibria exist in all three treatments. Specifically, both the defective equilibrium and several cooperative equilibria exist in all three treatments. A key distinction is that the defective Nash equilibrium exists in the four-player threshold game under misaligned inequality, but not in the two-player version.

For the repeated game, we apply the Folk theorem (6) to analyze the subgame perfect equilibrium (SPE). Let $\mathcal{F}$ denote the feasible payoff set, defined as the convex hull of all payoff profiles $\boldsymbol{\pi} = (\pi_1, \pi_2, \pi_3, \pi_4)$ that can be achieved through combinations of the players' contributions:

$$\mathcal{F} = \text{conv}\left\{ \big(\pi_1(\boldsymbol{c}), \pi_2(\boldsymbol{c}), \pi_3(\boldsymbol{c}), \pi_4(\boldsymbol{c})\big) \;\middle|\; c_1, c_2 \in S_1,\; c_3, c_4 \in S_2 \right\} \qquad [11]$$

The Folk theorem suggests that the payoffs that can be sustained in a SPE coincide with the payoffs that are feasible and provide each player with at least their maximin payoff (the payoff that players can guarantee themselves, even if their co-players act fully adversarially). In the linear game, the maximin payoff for player $i$ is simply $e_i$. Thus, the individual-rationality constraint is:

$$\pi_i \geq e_i \text{ for all } i. \qquad [12]$$

Therefore, the set of SPE payoffs is given by the intersection of the feasible set $\mathcal{F}$ and the individual-rationality constraints:

$$\mathcal{R}^L_{SPE} = \{\boldsymbol{\pi} \in \mathcal{F} \mid \pi_i \geq e_i \text{ for all } i\}. \qquad [13]$$

It is straightforward to verify that full cooperation satisfies the individual rationality constraints; hence, it can constitute a subgame perfect equilibrium in all three treatments. This contrasts with the two-player linear game, where full cooperation under misaligned inequality does not satisfy the conditions for individual rationality and thus cannot be sustained. Thus, we anticipate higher cooperation levels under misaligned inequality in the four-player experiment compared to the two-player setting.

In the threshold game, the maximin payoff depends on whether a player can reach the threshold unilaterally. The maximin payoff is given by:

$$\pi_i \geq \max\{e_i, e_i + r_i - \theta/p_i\} \text{ for all } i. \quad [14]$$

Therefore, the SPE payoff set is:

$$\mathcal{R}_{SPE}^T = \{\boldsymbol{\pi} \in \mathcal{F} \mid \pi_i \geq \max\{e_i, e_i + r_i - \theta/p_i\} \text{ for all } i\}. \quad [15]$$

All pure-strategy Nash equilibria of the one-shot threshold game can be repeated indefinitely to form subgame perfect equilibria of the repeated game. Since in none of our experimental treatments participants are able to reach the threshold all by themselves, the set $\mathcal{R}_{SPE}^T$ can be written as

$$\mathcal{R}_{\text{SPE}}^T = \{\boldsymbol{\pi} \in \mathcal{F} \mid \pi_i \geq e_i \text{ for all } i\}. \quad [16]$$

Within this SPE payoff set, the social optimum is attained when only the two high-productivity players contribute to reach the threshold $\theta$. Then, the group payoff satisfies $\sum_{i=1}^4 \pi_i \leq \sum_{i=1}^4 e_i + 4r - \theta/\max\{p_i\}$.

**B. Behavioral experiment.** We conducted a four-player version of both a linear public goods game and a threshold public goods game, each including three treatments: full equality (FE), aligned inequality (AI), and misaligned inequality (MI). To avoid potential carryover effects, individuals who had participated in the earlier two-player experiment were ineligible for the four-player experiment.

***Experimental design.*** The four-player experiment mirrors the two-player version, with each player type duplicated. Specifically, each group consists of two players of the original player 1 type (players 1 and 2) and two players of the original player 2 type (players 3 and 4).

Similar to before, participants are randomly assigned to one of six treatments (see **Table S15**). Each treatment comprises two game sessions and a post-game survey (see Appendices D and E). Contribution mechanisms are kept constant across sessions; example screenshots are provided in the appendices, and payoffs are computed according to the function in Eq. (9). Roles are fixed within each session, and the group composition remains constant during a session. In Session 2, groups are reshuffled, and roles are systematically swapped: those who played as player 1 or 2 in Session 1 now take on the role of player 3 or 4, and vice versa. The number of rounds per session matches that of the two-player game.

The treatment conditions are common knowledge within each treatment. However, participants only learn the rules of each part as they reach it in the experiment. For example, they are unaware of the rules for Session 2 while playing Session 1. Similarly, they do not know the content of the survey during the game phases.

***Experimental procedure.*** The four-player experiment was conducted in April 2025 at the computer lab of Beijing Normal University, involving 588 undergraduate students (see **Table S16**). As in the two-player experiment, participants were assigned to different treatments, received standardized instructions, and interacted anonymously via computer terminals, with all communication between participants strictly prohibited.

| Game | Treatments | # Participants | # Groups |
|---|---|---|---|
| Four-player linear PGG | Full equality | 100 | 25 |
| | Aligned inequality | 104 | 26 |
| | Misaligned inequality | 108 | 27 |
| Four-player threshold PGG | Full equality | 76 | 19 |
| | Aligned inequality | 100 | 25 |
| | Misaligned inequality | 100 | 25 |

**Table S16.** Number of participants and groups of each treatment in the four-player experiment.

The show-up fee and bonus conversion schemes differed slightly from those in the two-player setting to account for the change in group structure while maintaining incentive comparability. In the linear game, each participant's bonus was based on their cumulative payoffs and the outcomes of two hypothetical decisions from the post-game survey, with the total payoff converted at a rate of 1,000 points = 15 Chinese Yuan. In contrast, in the threshold game, only cumulative payoffs counted toward the bonus, converted at 100 points = 4 Chinese Yuan. Additionally, all participants received a fixed show-up fee of 15 Yuan. On average, participants earned 53.24 Yuan (∼6.52 EUR) in the linear game and 56.96 Yuan (∼6.97 EUR) in the threshold game.

| Four-player linear PGG | Group relative contributions | | | Group overall surplus | | |
|---|---|---|---|---|---|---|
| | Session 1 | Session 2 | Session 1 vs. Session 2 $p$-values | Session 1 | Session 2 | Session 1 vs. Session 2 $p$-values |
| FE | 58.9% | 67.7% | 0.2646 | 129.6% | 148.8% | 0.2646 |
| AI | 63.7% | 70.3% | 0.3321 | 157.4% | 175.2% | 0.3602 |
| MI | 66.5% | 68.1% | 0.9242 | 128.7% | 131.6% | 0.8559 |

**Table S17. Four-player linear PGG: Group relative contributions and overall surplus in Session 1 and Session 2.** Values in columns 2-3 and 5-6 are the average group relative contributions and the surplus in Session 1 and Session 2. Values in columns 4 and 7 are the original $p$-values of two-sided Mann-Whitney-Wilcoxon tests. The analysis is based on the relative contribution and the surplus of each group, averaged over all 20 rounds. To account for 6 simultaneous comparisons, the Bonferroni-corrected significance level becomes $0.05/6 \approx 0.0083$ (1 asterisk).

***Data Analysis.*** For the four-player game experiment, we apply the same statistical methods as in the two-player version. Specifically, we use two-sided Mann-Whitney-Wilcoxon tests for between-group comparisons and two-sided Wilcoxon signed-rank tests for within-group comparisons. We report original $p$-values. Where applicable, significance levels are adjusted for multiple testing using the Bonferroni correction.

**Linear game.** In **Table S17** on the linear game, the sample sizes are 50, 52, and 54, respectively (these correspond to the number of groups in the two sessions). Significance levels are adjusted for 6 simultaneous tests (3 treatments with 2 tests each). When comparing group relative contribution and group overall surplus across treatments, the same sample sizes apply, and significance levels are adjusted for 12 tests (pairwise comparisons across 3 treatments with 2 tests per comparison). Under the Bonferroni correction, the adjusted significance threshold for $p < 0.01$ becomes $0.01/6 \approx 0.0017$ (denoted by two asterisks). For comparisons of the first five rounds versus the last five rounds in group relative contribution, we use the Wilcoxon signed-rank test with the same sample sizes, adjusting significance levels for 3 simultaneous tests. For the Gini coefficient comparison, only the aligned inequality and misaligned inequality treatments are compared, as these two treatments have the same before-game Gini values and are therefore directly comparable. In **Table S18**, the sample sizes are 200, 208, and 216 (i.e., number of participants in the two sessions) for the three treatments, with significance levels adjusted for 6 simultaneous tests. Finally, in **Table S19**, the numbers of players of each type (players 1–4) are 50, 52, and 54 in the three treatments, respectively, and significance levels are adjusted for 10 simultaneous tests.

**Threshold game.** In **Table S21**, the sample sizes are 38, 50, and 50, respectively. Significance levels are adjusted for 6 simultaneous tests (3 treatments with 2 tests each). When comparing group success rate and group overall surplus across treatments, the same sample sizes apply, and significance levels are adjusted for 12 tests (pairwise comparisons across 3 treatments with 2 tests per comparison). For the Gini coefficient comparison, again only the aligned inequality and misaligned inequality treatments are compared. In **Table S22**, the sample sizes are 152, 200, and 200 (i.e., number of participants in the two sessions) for the three treatments, with significance levels adjusted for 6 simultaneous tests. Finally, in **Table S23**, the numbers of players in each type (players 1–4) are 38, 50, and 50 in the three treatments, respectively, and significance levels are adjusted for 10 simultaneous tests.

**C. Experimental results of the linear game.** Again, all results are based on an analysis of the first 20 rounds.

***Sessions comparisons.*** To examine whether behavioral outcomes are similar across experimental sessions, we compare two key measures between sessions. The first is the group relative contribution, calculated as $\sum_{i=1}^{4} c_i / \sum_{i=1}^{4} e_i$. The second is the group overall surplus, calculated as $(\sum_{i=1}^{4} \pi_i - \sum_{i=1}^{4} e_i) / \sum_{i=1}^{4} e_i$. For each group, we compute these quantities across all 20 rounds, and compare the respective averages between the two sessions. As shown in **Table S17**, we find no significant differences between sessions for either measure. Based on these results, we again pool the data from both sessions in the subsequent analysis.

***Treatment comparisons.*** In the main text, **Fig. 4** illustrates the comparison of group relative contribution, overall surplus, as well as payoff inequality across the three treatments. We first examine the players' relative contributions by computing the average relative contribution for each group across 20 rounds. In **Fig. 4A**, we find that group relative contributions are similar across treatments (FE: 63.3%, AI: 67.0%, MI: 67.3%), with no significant pairwise differences (FE vs. AI: $p = 0.5513$; FE vs. MI: $p = 0.6891$; AI vs. MI: $p = 0.8844$). We next analyze how contributions evolve over time (see **Fig. 4B**). The full equality treatment shows a significant decline: average relative contribution drops from 67.2% in the first 5 rounds to 56.2% in the last 5 rounds ($p = 0.0014$**, Bonferroni corrected for three tests). In contrast, both aligned and misaligned inequality treatments show a more stable dynamics (AI: $p = 0.0288$; MI: $p = 0.1375$).

For overall surplus, we compute the average over all 20 rounds for each group, see **Fig. 4C**. The aligned inequality treatment yields the highest average surplus (1.66), significantly exceeding the misaligned inequality treatment (1.30, $p = 0.0010$**). The difference between aligned inequality treatment and full equality treatment (1.39, $p = 0.0362$) is not statistically significant after Bonferroni correction. No significant difference exists between full equality treatment and misaligned inequality treatment ($p = 0.2325$).

| Four-player linear PGG | Absolute contributions | | | Relative contributions | | |
|---|---|---|---|---|---|---|
| | Role 1 (Player 1 & 2) | Role 2 (Player 3 & 4) | Role 1 vs. Role 2 $p$-values | Role 1 (Player 1 & 2) | Role 2 (Player 3 & 4) | Role 1 vs. Role 2 $p$-values |
| FE | 15.09 | 15.28 | 0.2796 | 63% | 64% | 0.2775 |
| AI | 23.63 | 8.54 | <0.0001*** | 66% | 71% | 0.0093* |
| MI | 23.28 | 9.00 | <0.0001*** | 65% | 75% | 0.0002** |

**Table S18. Comparison of absolute and relative contributions of the two roles in the four-player linear PGG.** Values in columns 2-3 contain the average absolute contributions of the two role players, whereas columns 5-6 contain their relative contributions. Values in columns 4 and 7 are the original $p$-values of paired Wilcoxon signed-rank tests. The analysis is based on the contributions at the individual level (averaged over 20 rounds during which the given two players interact). To account for 6 simultaneous comparisons, the Bonferroni-corrected significance levels become $0.05/6 \approx 0.0083$ (1 asterisk), $0.01/6 \approx 0.0017$ (2 asterisks) and $0.001/6 \approx 0.00017$ (3 asterisks).

| Four-player linear PGG | | Player 1 | Player 2 | Player 3 | Player 4 |
|---|---|---|---|---|---|
| FE | Contributions | 15.23 | 14.95 | 14.80 | 15.76 |
| | $p$-values | P1 vs P2: 0.8506; | | P1 vs P3: 0.8393; | |
| | | P1 vs P4: 0.1955; | | P2 vs P3: 0.5115; | |
| | | P2 vs P4: 0.4778; | | P3 vs P4: 0.1358 | |
| AI | Contributions | 23.87 | 23.39 | 8.87 | 8.22 |
| | $p$-values | P1 vs P2: 0.3648; | | P3 vs P4: 0.0793 | |
| MI | Contributions | 23.16 | 23.41 | 8.89 | 9.11 |
| | $p$-values | P1 vs P2: 0.8162; | | P3 vs P4: 0.7208 | |

**Table S19. Four-player linear PGG: Within-role comparison of absolute contributions.** The analysis is based on each player's average contribution over the 20 rounds. All $p$-values reported in the table are original values from two-sided Wilcoxon signed-rank tests. A Bonferroni correction for 10 comparisons is applied ($\alpha = 0.005$), but none of the results are statistically significant.

We measure payoff inequality using each group Gini coefficient in each round,given by $\frac{1}{32\bar{\pi}} \sum_{i=1}^{4} \sum_{j=1}^{4} |\pi_i - \pi_j|$, where $\pi_i$ is player $i$'s payoff and $\bar{\pi}$ is the group average payoff. We average each group Gini coefficients over all 20 rounds for statistical comparison between treatments. As shown in **Fig. 4D**, the full equality treatment shows a slight increase in inequality from 0 (equal initial endowments) to 0.039 after the game. Aligned inequality and misaligned inequality treatments start from identical pre-game Gini coefficients based on endowments. Both treatments show similar reductions in inequality, with final values of 0.074 and 0.075 respectively ($p = 0.7568$). This finding differs from the two-player experiment, where aligned inequality leads to a significantly greater inequality reduction than misaligned inequality.

Our main findings remain consistent with the two-player experiment: aligned inequality promotes cooperation, as evidenced by increased group relative contributions and surplus compared to full equality. However, misaligned inequality does not reduce cooperation in the four-player setting. As one possible reason for this difference, we again note that full cooperation constitutes a subgame perfect equilibrium under misaligned inequality in the four-player game.

***Contribution comparisons between players.*** To investigate role-based behavioral differences in the four-player game, we categorize player 1 and player 2 as Role 1, and player 3 and player 4 as Role 2. For each group, we compute each player's 20-round average contribution and then average these values across players in the same role for comparison. **Table S18** shows that in both aligned inequality and misaligned inequality treatments, Role 1 players contribute significantly more in absolute terms but less in relative terms than Role 2 players. This role-based asymmetry is consistent with our findings from the two-player linear game.

Next, we assess whether players assigned the same role behave similarly. We begin by comparing the two same-role players within each group in terms of their 20-round average contributions. As shown in **Table S19** and **Fig. S21A,B**, there are no significant differences between same-role players, regardless of treatment or role. However, aggregate similarity does not imply identical contributions in every round. To investigate within-role consistency at the round level, we compute the absolute difference in contributions between same-role players for each round and then average these differences over all 20 rounds. **Fig. S22A,B** display the distribution of these average round-level differences. On average, the relative difference in contributions between same-role players is approximately 20%, consistent across all treatments and roles (see **Table S20**). This indicates that although same-role players contribute similarly when averaged across rounds, substantial individual variation remains at the round-by-round level.

***Conditional behaviors.*** As in the two-player game, we examine whether participants in the four-player game adjust their contributions based on the behavior of others. Given the larger group size in the four-player game, we examine how player's contribution $c_i$ depends on the collective contribution of the other three group members, calculated as $\sum_{j \neq i} p_j c_j$, where $c_j$ is player $j$'s contribution and $p_j$ is their productivity. We apply the same analysis to the post-game survey, in which participants

| Four-player linear PGG | Absolute difference in absolute contributions | | Absolute difference in relative contributions | |
|---|---|---|---|---|
| | Role 1<br>$\lvert c_1 - c_2 \rvert$ | Role 2<br>$\lvert c_3 - c_4 \rvert$ | Role 1<br>$\lvert c_1/e_1 - c_2/e_2 \rvert$ | Role 2<br>$\lvert c_3/e_3 - c_4/e_4 \rvert$ |
| FE | 5.12 | 5.22 | 21.3% | 21.7% |
| AI | 8.21 | 2.97 | 22.8% | 24.7% |
| MI | 6.97 | 2.67 | 19.4% | 22.3% |

**Table S20. Same-role contribution differences in the four-player linear PGG.** For each group in the four-player linear public goods game, we compute the absolute difference in contributions between the two players with the same role in each round. These values are then averaged over all rounds for each group, and the reported results are the averages across all groups.

indicated how much they would contribute under several hypothetical scenarios specifying the contributions of the other players (see Section 5D). In **Fig. S23**, we observe conditional contribution behaviors in both the actual game and the survey response. Players' contributions increase with the collective contribution of their group members, consistent with a direct reciprocity mechanism.

**D. Experimental results of the threshold game.** We apply the same behavioral analysis to the threshold game as used for the four-player linear game. However, we replace "group relative contribution" with "group success rate" when comparing session differences and treatment differences, as success rate is the more relevant measure for cooperation level in the threshold game.

***Session comparisons.*** We begin by comparing the two sessions of the four-player threshold game. Here, group-level cooperation is measured by the success rate, defined as the proportion of rounds in which the group successfully reached the threshold. In addition, we consider group overall surplus. As shown in **Table S21**, there are no significant differences in either success rate or surplus between the two sessions. We therefore pool the data across sessions for all subsequent analyses.

| Four-player threshold PGG | Group success rates | | | Group overall surplus | | |
|---|---|---|---|---|---|---|
| | Session 1 | Session 2 | Session 1 vs. Session 2<br>$p$-values | Session 1 | Session 2 | Session 1 vs. Session 2<br>$p$-values |
| FE | 82.9% | 97.1% | 0.2076 | 22.6% | 30.8% | 0.1546 |
| AI | 44.8% | 51.2% | 0.6803 | 0.9% | 9.5% | 0.0991 |
| MI | 53.0% | 64.6% | 0.2414 | 8.6% | 16.3% | 0.1744 |

**Table S21. Four-player threshold PGG: Group success rates and overall surplus in Session 1 and Session 2.** Values in columns 2-3 and 5-6 are the average group success rates and the surplus in Session 1 and Session 2. Values in columns 4 and 7 are the original $p$-values of two-sided Mann-Whitney-Wilcoxon tests. The analysis is based on the success rate and the surplus of each group, averaged over all 20 rounds. To account for 6 simultaneous comparisons, the Bonferroni-corrected significance level becomes $0.05/6 \approx 0.0083$ (1 asterisk).

***Treatment comparisons.*** Success rates differ significantly across treatments (**Fig. 4E**). In the full equality treatment, 90.0% groups reach the threshold, significantly outperforming both aligned inequality (48.0%) and misaligned inequality (58.8%) treatments ($p < 0.0001$***). The difference between the two asymmetric treatments is not statistically significant ($p = 0.2296$). Compared with the two-player game, success rates and surplus under aligned or misaligned inequality are lower in the four-player setting, suggesting increased coordination challenges in larger groups.

We find a similar outcome for group overall surplus (**Fig. 4G**). Full equality generates the highest surplus (26.7%), followed by misaligned inequality (12.4%) and aligned inequality (5.2%). All pairwise differences are statistically significant, with full equality outperforming both aligned and misaligned inequality ($p < 0.0001$***) and misaligned inequality exceeding aligned inequality ($p = 0.0441$). In terms of payoff inequality (**Fig. 4H**), both asymmetric treatments lead to comparable levels of inequality (AI: 0.21, MI: 0.20; $p = 0.6516$). This contrasts with the two-player threshold game, where aligned inequality leads to a greater reduction Gini coefficients than misaligned inequality.

Overall, our main conclusions are consistent with the two-player experiment. Both asymmetric treatments result in less cooperation, relative to full equality.

***Contribution comparisons between players.*** As in the four-player linear game, we categorize players 1 and 2 as Role 1, and players 3 and 4 as Role 2. We first compare contributions across roles. In both asymmetric treatments, Role 1 players contribute significantly more in absolute terms but less in relative terms than Role 2 players. The only exception occurs under the aligned inequality treatment, where the relative contributions do not differ significantly (see **Table S22**). This observation contrasts with the two-player threshold game, where the more productive and higher-endowment player contributes more in both absolute and relative terms.

We next compare the two same-role players within each group in terms of their 20-round average contributions. As shown in **Table S19** and **Fig. S22C,D**, there are no significant differences between same-role players across roles or treatments. Furthermore,

| Four-player threshold PGG | Absolute contributions | | | Relative contributions | | |
|---|---|---|---|---|---|---|
| | Role 1 (Player 1 & 2) | Role 2 (Player 3 & 4) | Role 1 vs. Role 2 $p$-values | Role 1 (Player 1 & 2) | Role 2 (Player 3 & 4) | Role 1 vs. Role 2 $p$-values |
| FE | 11.48 | 11.71 | 0.6457 | 47.8% | 48.8% | 0.6351 |
| AI | 12.02 | 4.69 | <0.0001*** | 33.4% | 39.1% | 0.0171 |
| MI | 11.95 | 5.60 | <0.0001*** | 33.2% | 46.6% | 0.0001*** |

**Table S22. Comparison of absolute and relative contributions of the two roles in the four-player threshold PGG.** Values in columns 2-3 contain the average absolute contributions of the two role players, whereas columns 5-6 contain their relative contributions. Values in columns 4 and 7 are the original $p$-values of paired Wilcoxon signed-rank tests. The analysis is based on the contributions at the individual level (averaged over 20 rounds during which the given two players interact). To account for 6 simultaneous comparisons, the Bonferroni-corrected significance levels become $0.001/6 \approx 0.00017$ (3 asterisks).

| Four-player threshold PGG | | Player 1 | Player 2 | Player 3 | Player 4 |
|---|---|---|---|---|---|
| FE | Contributions | 11.50 | 11.46 | 11.96 | 11.47 |
| | $p$-values | P1 vs P2: 0.3504; | | P1 vs P3: 0.9696; | |
| | | P1 vs P4: 0.5833; | | P2 vs P3: 0.9671; | |
| | | P2 vs P4: 0.5843; | | P3 vs P4: 0.8998 | |
| AI | Contributions | 11.94 | 12.10 | 4.71 | 4.67 |
| | $p$-values | P1 vs P2: 0.6780; | | P3 vs P4: 0.4661 | |
| MI | Contributions | 11.83 | 12.08 | 5.67 | 5.52 |
| | $p$-values | P1 vs P2: 0.7758; | | P3 vs P4: 0.5718 | |

**Table S23. Four-player threshold PGG: Within-role comparison of absolute contributions.** The analysis is based on each player's average contribution over the 20 rounds. All $p$-values of two-sided Wilcoxon signed-rank tests are original. A Bonferroni correction for 10 comparisons was applied ($\alpha = 0.005$), but none of the results are statistically significant.

| Four-player threshold PGG | Absolute difference in absolute contributions | | Absolute difference in relative contributions | |
|---|---|---|---|---|
| | Role 1 $\lvert c_1 - c_2 \rvert$ | Role 2 $\lvert c_3 - c_4 \rvert$ | Role 1 $\lvert c_1/e_1 - c_2/e_2 \rvert$ | Role 2 $\lvert c_3/e_3 - c_4/e_4 \rvert$ |
| FE | 0.89 | 1.15 | 3.7% | 4.8% |
| AI | 3.84 | 2.32 | 10.7% | 19.4% |
| MI | 3.97 | 1.74 | 11.0% | 14.5% |

**Table S24. Same-role contribution differences in the four-player threshold PGG.** For each group in the four-player threshold public goods game, we compute the absolute difference in contributions between the two players with the same role in each round. These values are then averaged over all rounds for each group, and the reported results are the averages across all groups.

to assess within-role consistency at the round level, we compute the absolute difference in contributions between same-role players for each round and then average these differences over 20 rounds (see **Table S24**). Notably, these within-role differences appear to be smaller than in the four-player linear game.

***Conditional behaviors.*** To examine the players' conditional behavior, we investigate the extent to which participants in the four-player game adjust their contributions based on the expected behavior of others.

Unlike the four-player linear game, the survey in the threshold public goods game asked players to report the minimum total contribution they expected from the other three players for successful coordination. Accordingly, in **Fig. S24**, we plot these reported expectations on the x-axis and players' own contributions under the associated cooperative equilibria on the y-axis.

When the other three players contribute around 50% of their endowments, players' conditional behavior cluster around the threshold line, $\sum_{j \neq i} p_j c_j(t-1) + p_i c_i(t) = \theta$. This suggests that players adjust their behavior conditionally to help meet the threshold. Overall, the patterns of conditional contributions in the four-player experiment closely resemble those observed in the two-player experiment.

## 5. Further materials

**A. Informed consent for behavioral experiment.** The following document provides participants with information regarding their duties and rights in the behavioral experiment, and records their acknowledgment and consent.

**Participant responsibilities**
1. Participants shall provide accurate personal information (e.g., name, gender, student identification number).
2. Participation is voluntary. Participants may withdraw from the experiment at any time without incurring any penalty.
3. Participants shall complete the experiment diligently, comply with all instructions given by the experimenters, and refrain from communicating with individuals other than authorized staff during the experiment.
4. Participants shall not disclose any details of the experimental procedures or content to third parties following participation.

**Researcher responsibilities**
1. The researchers shall provide monetary compensation to participants upon successful completion of the experiment.
2. The researchers shall keep participants' registration information strictly confidential and shall not disclose it to any third party, except in the following circumstances:

(a) where explicit authorization has been obtained from the participant in advance;

(b) where disclosure is required by applicable laws or regulations.

3. The researchers reserve the right to analyze the experimental data and to use such data for academic purposes, including publications and presentations, provided that no personal information or identifying details of the participants are disclosed.

**B. Instructions of the two-player linear public goods game.** In the following, we present the instructions for the aligned inequality treatment as an example. The following instructions are translated from Chinese.

**General rules**
You will play 2 sessions of decision-making games.
In each session, the system will randomly match another player for you.
There are two roles in the games. You and the co-player are randomly assigned the role of player 1 and player 2.
Neither of you knows each other's true identities.
Your bonus will be determined by the choices made by you and the co-player.
Your total income = 20 Yuan (show-up fee) + 0.03 Yuan × (the cumulative points from two sessions + the survey points).

**Introduction to Session 1**
In the first round, you and a co-player are randomly assigned the role of player 1 and player 2.
You will repeat the game for at least 20 rounds. After the 20th round, there will be a 50% chance that the game will continue for another round. Neither of your roles will be changed during the game.
In each round, player 1 has an initial 36 points and player 2 has an initial 12 points. You each decide how many points you would like to contribute to the common pool and how many to keep for yourself.
For every point that player 1 contributes, the common pool will increase by 1.9 points. For every point that player 2 contributes, the common pool will increase by 1.3 points.
In each round, the points in the common pool will be split evenly between the two players. Your payoff per round is the sum of the points you keep for yourself in that round and the points you receive from the common pool.
At the end of each round, both of you can see the results about your contributions and payoffs in the round, the group total contribution and returns from the common pool.

**Exercise 1**
If you are player 1, you contribute 0 points and the co-player contributes 0 points.
The total collective contribution in the common pool are ( ).
Your payoff is ( ) points.
The co-player's payoff is ( ) points.

**Exercise 2**
If you are player 1, you contribute 36 points and the co-player contributes 12 points.
The total collective contribution in the common pool is ( ) points.
Your payoff is ( ) points.
The co-player's payoff is ( ) points.

**Introduction to Session 2**
Your role in Session 2 is different from that in Session 1.
You will play at least 20 rounds with a player randomly assigned by the system. Both of your roles will remain unchanged during the game.
Session 2 has the same process as Session 1.

**Screenshots of the experimental interface**
Here we provide screenshots of the aligned inequality treatment to illustrate the differences between player 1 (left) and player 2

(right). The experimental interface for the other treatments are analogous.

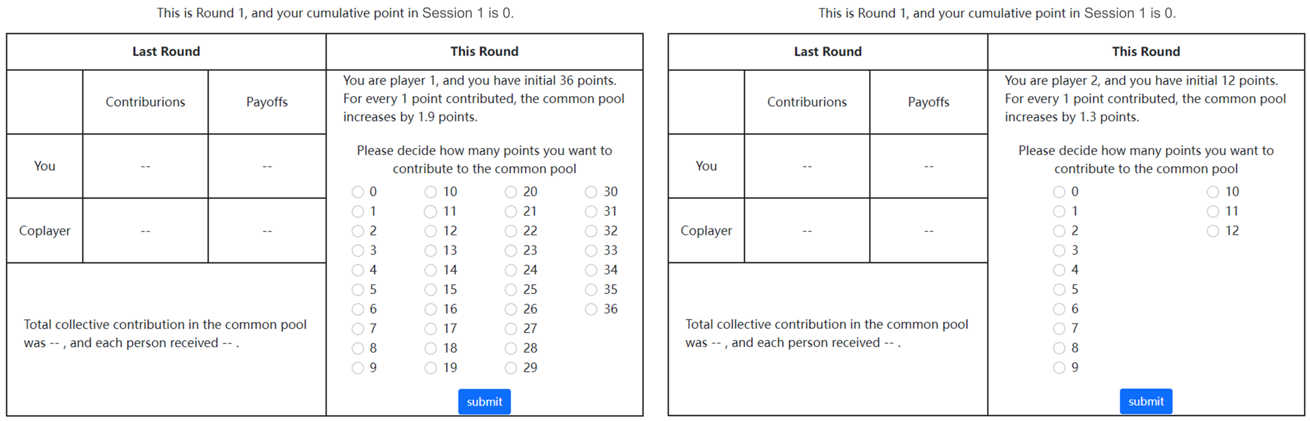


## Survey

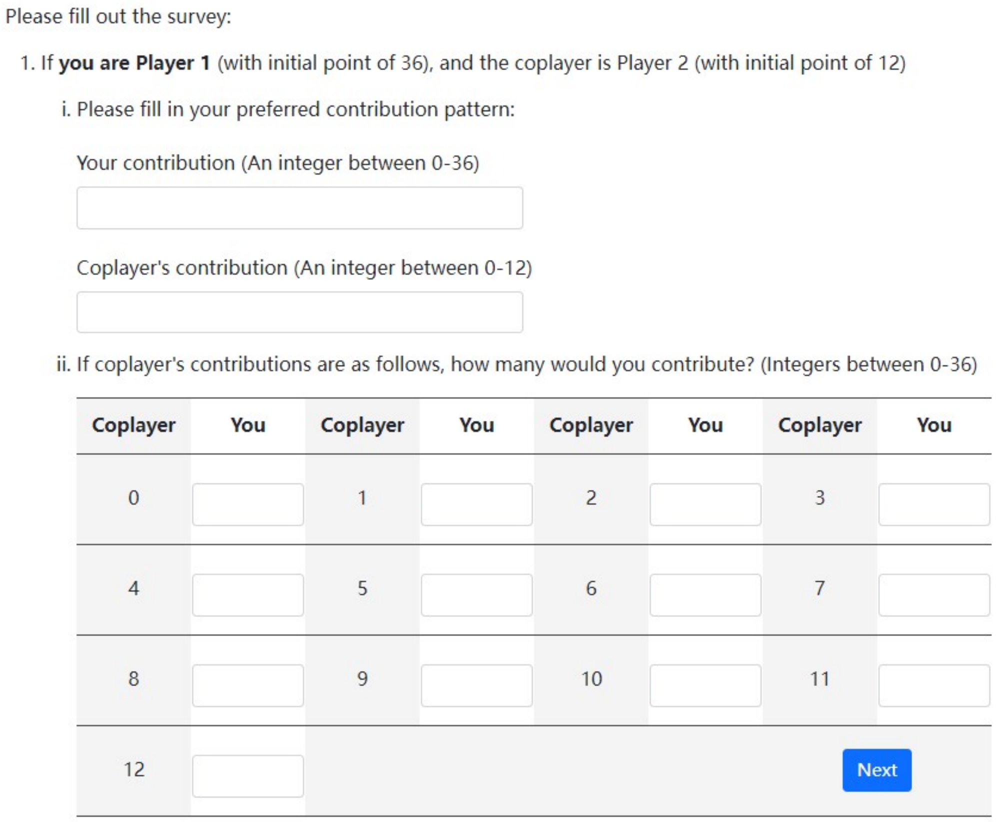

2. If **you are Player 2** (with initial point of 12), and the coplayer is Player 1 (with initial point of 36)

i. Please fill in your preferred contribution pattern:

Your contribution (An integer between 0-12)

Coplayer's contribution (An integer between 0-36)

ii. If coplayer's contributions are as follows, how many would you contribute? (Integers between 0-12)

| Coplayer | You | Coplayer | You | Coplayer | You | Coplayer | You |
|---|---|---|---|---|---|---|---|
| 0 | | 1 | | 2 | | 3 | |
| 4 | | 5 | | 6 | | 7 | |
| 8 | | 9 | | 10 | | 11 | |
| 12 | | 13 | | 14 | | 15 | |
| 16 | | 17 | | 18 | | 19 | |
| 20 | | 21 | | 22 | | 23 | |
| 24 | | 25 | | 26 | | 27 | |
| 28 | | 29 | | 30 | | 31 | |
| 32 | | 33 | | 34 | | 35 | |
| 36 | | | | | | Next | |

**C. Instructions of the two-player threshold public goods game.** In the following, we present the instructions for the aligned inequality treatment as an example. The following instructions are translated from Chinese.

### General rules

You will play 2 sessions of decision-making games.
In each session, the system will randomly match another player for you.
There are two roles in the games. You and the co-player are randomly assigned the role of player 1 and player 2.
Neither of you knows each other's true identities.
Your bonus will be determined by the choices made by you and the co-player.
Your total income = 20 Yuan (show-up fee) + 0.03 Yuan × cumulative points from two sessions.

### Introduction to Session 1

You will play 20 rounds with the co-player. Neither of your roles will be changed during the game.
In each round, player 1 has initial an 36 points and player 2 has an initial 12 points. You each decide how many points you would like to contribute to the common pool and how many to keep for yourself.
For every point that player 1 contributes, the common pool will increase by 3 points. For every point that player 2 contributes, the common pool will increase by 1 point. In each round, if the total collective contribution in the common pool is greater than or equal to 60, both of you will be awarded 20 points in addition to the remaining points. However, if the total collective contribution is less than 60, no additional points will be awarded.
At the end of each round, both of you can see the results about your contributions and payoffs in the round, the total contribution and whether you have reached the threshold.

### Exercise 1

If you are player 1, you contribute 0 points and the co-player contributes 0 points.
The total points in the common pool are (  ).
Does the total collective contribution reach the goal? (  )
Your payoff is (  ) points.
The co-player's payoff is (  ) points.

### Exercise 2

If you are player 1, you contribute 36 points and the co-player contributes 12 points.
The total points in the common pool are (  ).
Does the total collective contribution reach the goal? (  )
Your payoff is (  ) points.
The co-player's payoff is (  ) points.

### Introduction to Session 2

Your role in Session 2 is different from that in Session 1.
You will play 20 rounds with a player randomly assigned by the system. Both of your roles will remain unchanged during the game.
Session 2 has the same process as Session 1.

### Screenshots of the experimental interface

Here we provide screenshots of the aligned inequality treatment to illustrate the differences between player 1 (left) and player 2 (right). The experimental interface for the other treatments are analogous.

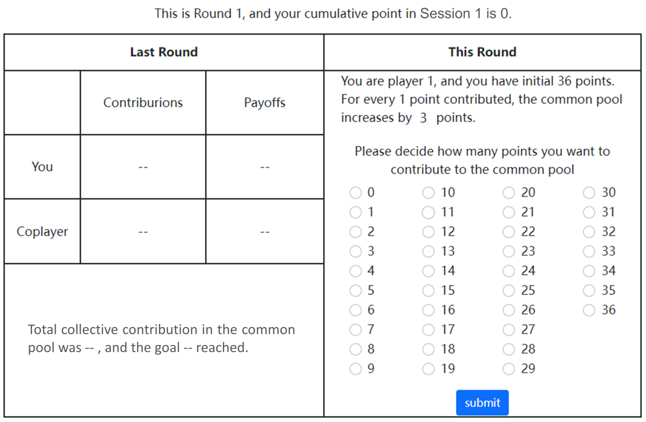


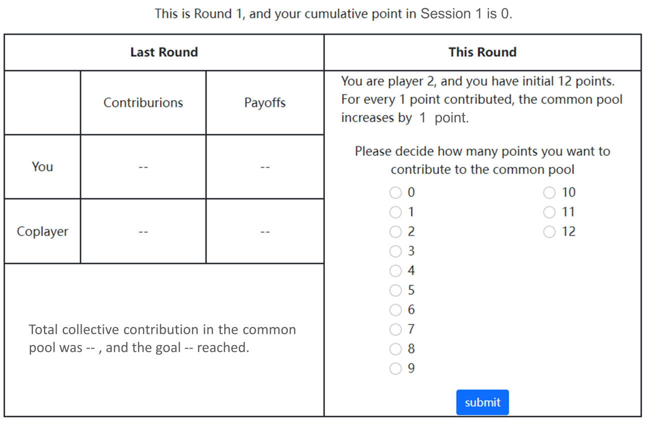

**Survey**
Please fill out the questionnaire:
1. If you are Player 1 (with initial point of 36), the coplayer is Player 2 (with initial point of 12).
i. At a fair contribution pattern:
Your contribution should be _____.
The contribution of the coplayer should be _____.
ii. What is the minimum contribution of the coplayer such that you are willing to contribute the other part to make the total collective contribution of the common pool reach the goal? _____
2. If you are Player 2 (with initial point of 12), the coplayer is Player 1 (with initial point of 36).
i. At a fair contribution pattern:
Your contribution should be _____.
The contribution of the coplayer should be _____.
ii. What is the minimum contribution of the coplayer such that you are willing to contribute the other part to make the total collective contribution of the common pool reach the goal? _____

**D. Instructions for the four-player linear public goods game.** In the following, we present the instructions for the aligned inequality treatment as an example. The following instructions are translated from Chinese.

**General rules**
You will play 2 sessions of decision-making games. In each session, the system will randomly match you with three other players and assign the roles of Player 1, Player 2, Player 3, and Player 4.
None of you will know each other's true identities.
Your bonus will be determined by the choices made by you and the other three players.
Your total income = 15 Yuan (show-up fee) + 0.015 Yuan × (the cumulative points from two sessions + the survey points).

**Introduction to Session 1**
You and the other three players will repeat the game for at least 20 rounds. After the 20th round, there will be a 50% chance that the game will continue for another round. Neither of your roles will be changed during the game.
In each round, Player 1 and Player 2 each have an initial 36 points, and Player 3 and Player 4 each have an initial 12 points.
Each player decides how many points to contribute to the common pool and how many to keep for themselves.
For every point that Player 1 or Player 2 contributes, the common pool will increase by 3.8 points. For every point that Player 3 or Player 4 contributes, the common pool will increase by 2.6 points.
In each round, the points in the common pool will be split evenly among all four players. Your payoff per round is the sum of the points you keep for yourself in that round and the points you receive from the common pool.
At the end of each round, all four players can see each other's contributions to the common pool and the payoffs in that round.

**Exercise 1**
If you are Player 1 and you contribute 0 points, and the other three players also contribute 0 points.
The total points in the common pool are ( ), and each player receives ( ) points from the common pool.
Your payoff is ( ) points. The payoffs of Player 2, Player 3, and Player 4 are ( ), ( ), and ( ), respectively.

**Exercise 2**
If you are Player 1 and you contribute 36 points, and Player 2, Player 3, and Player 4 contribute 36, 12, and 12 points, respectively.
The total points in the common pool are ( ), and each player receives ( ) points from the common pool.
Your payoff is ( ) points. The payoffs of Player 2, Player 3, and Player 4 are ( ), ( ), and ( ), respectively.

**Introduction to Session 2**
The system will reassign participants to new groups based on their roles in Session 1.
You will again play with three other players for at least 20 rounds. After the 20th round, there will be a 50% chance that the game will continue for another round.
Participants who were assigned as Player 1 or Player 2 in Session 1 will become Player 3 or Player 4 in Session 2.
Participants who were assigned as Player 3 or Player 4 in Session 1 will become Player 1 or Player 2 in Session 2.
Session 2 follows exactly the same rules and procedures as Session 1.

**Screenshots of the experimental interface**
Here we provide screenshots of the aligned inequality treatment to illustrate the differences between players 1 & 2 (left) and players 3 & 4 (right). The experimental interfaces for the other treatments are analogous.

**Survey**
Please fill out the questionnaire:

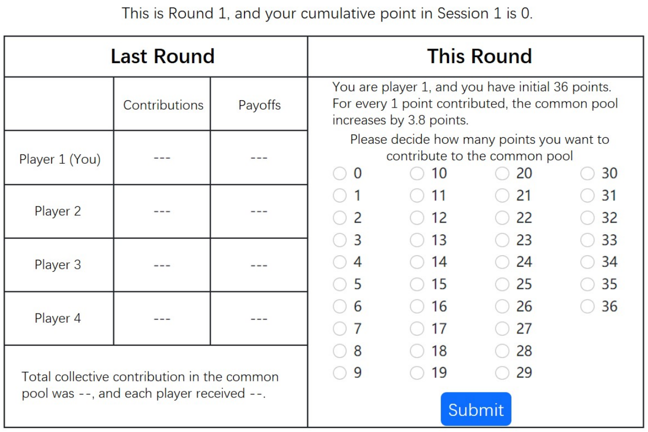


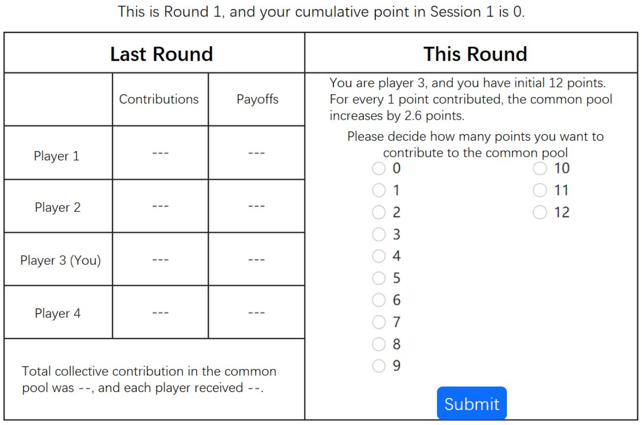


1. If you are Player 1 or 2

(i) Please fill in your preferred contribution pattern:

Player 1's contribution (An integer between 0 and 36): ____________

Player 2's contribution (An integer between 0 and 36): ____________

Player 3's contribution (An integer between 0 and 12): ____________

Player 4's contribution (An integer between 0 and 12): ____________

(ii) Suppose you are Player 1. Player 2 contributes the value in the left column (percentage = contribution / endowment), and the average contribution of Players 3 and 4 is the value in the top row (percentage = contribution / endowment). In each case, how many points would you contribute? (Enter an integer between 0 and 36 in each cell)

| Player 3&4 Average Contribution / Player 2 Contribution | 0 (0%) | 3 (25%) | 6 (50%) | 9 (75%) | 12 (100%) |
|---|---|---|---|---|---|
| 0 (0%) | | | | | |
| 9 (25%) | | | | | |
| 18 (50%) | | | | | |
| 27 (75%) | | | | | |
| 36 (100%) | | | | | |

2. If you are Player 3 or 4

(i) Please fill in your preferred contribution pattern:

Player 1's contribution (An integer between 0 and 36): ____________

Player 2's contribution (An integer between 0 and 36): ____________

Player 3's contribution (An integer between 0 and 12): ____________

Player 4's contribution (An integer between 0 and 12): ____________

(ii) Suppose you are Player 3. The average contribution of Players 1 and 2 is the value in the left column (percentage = contribution / endowment), and Player 4's contribution is the value in the top row (percentage = contribution / endowment). In each case, how many points would you contribute? (Enter an integer between 0 and 12 in each cell)

| Player 1&2 Average contribution \ Player 4 Contribution | 0 (0%) | 3 (25%) | 6 (50%) | 9 (75%) | 12 (100%) |
|---|---|---|---|---|---|
| 0 (0%) | | | | | |
| 9 (25%) | | | | | |
| 18 (50%) | | | | | |
| 27 (75%) | | | | | |
| 36 (100%) | | | | | |

Note: For the full equality treatment, question (ii) is similar to the two-player experiment: (ii) When the average contribution of the other three players is the following amount, how many points would you contribute? (Enter an integer between 0 and 24 in each cell)

| Others | You | Others | You | Others | You | Others | You |
|---|---|---|---|---|---|---|---|
| 0 | | 1 | | 2 | | 3 | |
| 4 | | 5 | | 6 | | 7 | |
| 8 | | 9 | | 10 | | 11 | |
| 12 | | 13 | | 14 | | 15 | |
| 16 | | 17 | | 18 | | 19 | |
| 20 | | 21 | | 22 | | 23 | |
| 24 | | | | | | Submit | |

**E. Instructions for the four-player threshold public goods game.** In the following, we present the instructions for the aligned inequality treatment as an example. The following instructions are translated from Chinese.

**General rules**
You will play 2 sessions of decision-making games. In each session, the system will randomly match you with three other players and assign the roles of Player 1, Player 2, Player 3, and Player 4.
None of you will know each other's true identities.
Your bonus will be determined by the choices made by you and the other three players.
Your total income = 15 Yuan (show-up fee) + 0.04 Yuan × the cumulative points you earn from both sessions.

**Introduction to Session 1**
You and the other three players will repeat the game for 20 rounds. Neither of your roles will be changed during the game.
In each round, Player 1 and Player 2 each have an initial 36 points, and Player 3 and Player 4 each have an initial 12 points.
Each player decides how many points to contribute to the common pool and how many to keep for themselves.
For every point that Player 1 or Player 2 contributes, the common pool will increase by 3 points. For every point that Player 3 or Player 4 contributes, the common pool will increase by 1 point.
If the total points in the common pool in a round are greater than or equal to 120, then in addition to the points they keep for themselves, each player will receive an extra 20 points. If the total points in the common pool are less than 120, no extra points will be awarded.
At the end of each round, all four players can see each other's contributions to the common pool and the payoffs in that round.

**Exercise 1**
If you are Player 1 and you contribute 0 points, and the other three players also contribute 0 points.
The total points in the common pool are ( ), and whether the target value is reached: ( ).

Your payoff is ( ) points. The payoffs of Player 2, Player 3, and Player 4 are ( ), ( ), and ( ), respectively.

**Exercise 2**
If you are Player 1 and you contribute 36 points, and Player 2, Player 3, and Player 4 contribute 36, 12, and 12 points, respectively.
The total points in the common pool are ( ), and whether the target value is reached: ( ).
Your payoff is ( ) points. The payoffs of Player 2, Player 3, and Player 4 are ( ), ( ), and ( ), respectively.

**Introduction to Session 2**
The system will reassign participants to new groups based on their roles in Session 1.
You will again play with three other players for 20 rounds.
Participants who were assigned as Player 1 or Player 2 in Session 1 will become Player 3 or Player 4 in Session 2.
Participants who were assigned as Player 3 or Player 4 in Session 1 will become Player 1 or Player 2 in Session 2.
Session 2 follows exactly the same rules and procedures as Session 1.

**Screenshots of the experimental interface**
Here we provide screenshots of the aligned inequality treatment to illustrate the differences between players 1 & 2 (left) and players 3 & 4 (right). The experimental interfaces for the other treatments are analogous.

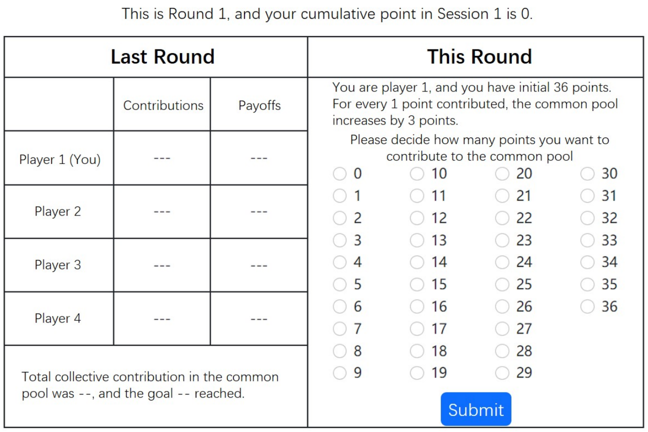


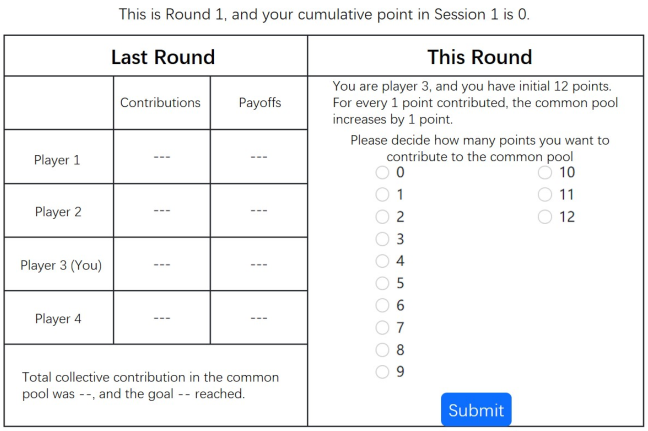


**Survey**
Please fill out the questionnaire:
1. If you are Player 1 or 2
(i) Please fill in what you consider to be a fair contribution pattern:
Player 1's contribution (An integer between 0 and 36): ____________
Player 2's contribution (An integer between 0 and 36): ____________
Player 3's contribution (An integer between 0 and 12): ____________
Player 4's contribution (An integer between 0 and 12): ____________
(ii) What is the minimum contribution from the other 3 players that would make you willing to contribute the remainder to reach a total of 120 points?
Minimum collective contribution from the other 3 players (An integer from 0-120) ____________
Your contribution should be (An integer from 0–36) ____________

2. If you are Player 3 or 4
(i) Please fill in what you consider to be a fair contribution pattern:
Player 1's contribution (An integer between 0 and 36): ____________
Player 2's contribution (An integer between 0 and 36): ____________
Player 3's contribution (An integer between 0 and 12): ____________
Player 4's contribution (An integer between 0 and 12): ____________
(ii) What is the minimum contribution from the other 3 players that would make you willing to contribute the remainder to reach a total of 120 points?
Minimum collective contribution from the other 3 players (An integer from 0-120) ____________
Your contribution should be (An integer from 0–12) ____________

## Supplementary figures

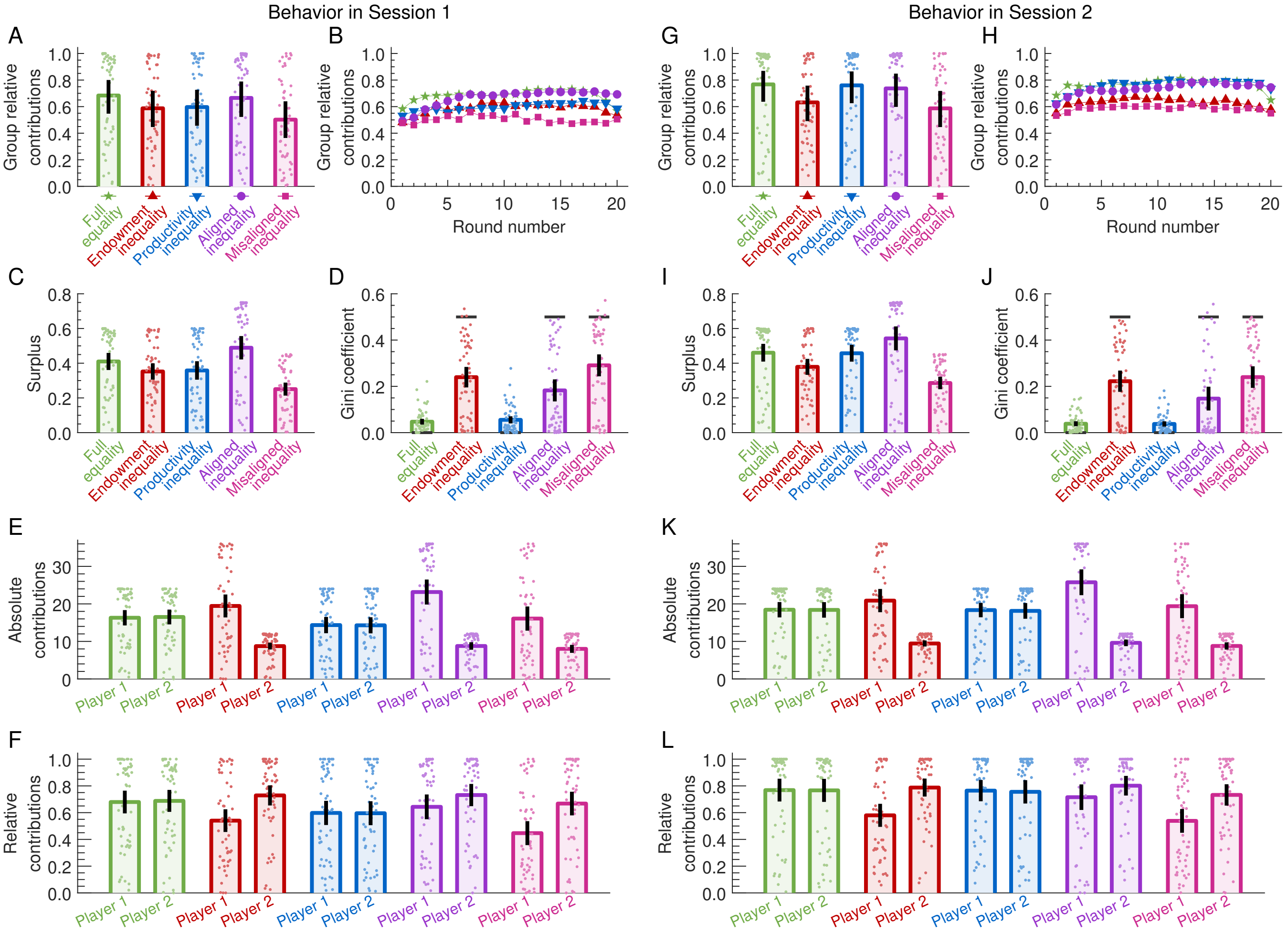


**Fig. S1. Experimental results from Session 1 and Session 2 in the linear PGG.** The experiment featured two separate sessions, each spanning 20 rounds with different partners for the participants. We find that the results from both sessions were qualitatively similar. However, in the productivity inequality treatment, players contribute more in Session 2 (panel **E** and panel **K**), leading to a significantly higher group relative contribution (panels **A** and **G**) and overall surplus (panels **C** and **I**) in Session 2. This is because players contribute more from the start of Session 2 (panels **B** and **H**). Error bars represent the respective 95% confidence intervals.

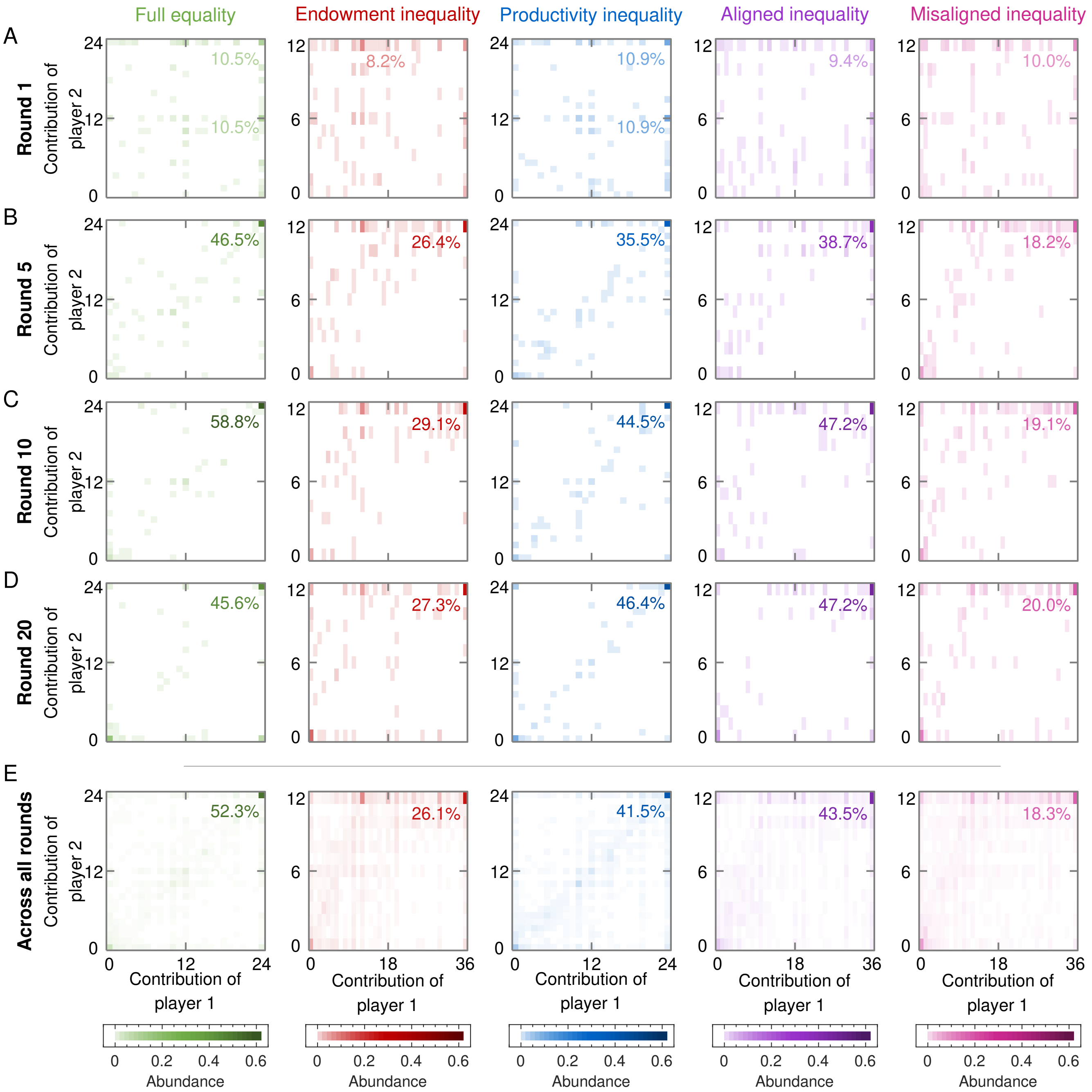


**Fig. S2. Cooperation dynamics over time in the linear PGG.** In this figure, we present snapshots for 4 different time points (or round numbers, see rows 1-4), and the overall contribution patterns across all rounds (see the bottom row). These snapshots allow us to explore in more detail how players change their strategies over time. The intensity of shading within a rectangle reflects the frequency of the corresponding outcome observed across all groups, while the numbers indicate the most abundant outcome. In the linear public goods game experiment, full cooperation emerges as the most abundant across all five treatments. In aligned inequality treatment, nearly 50% of groups achieve full cooperation at a pace comparable to those in the treatments with full equality and productivity inequality. However, the misaligned inequality treatment exhibits the lowest proportion of full cooperation at all observed time points.

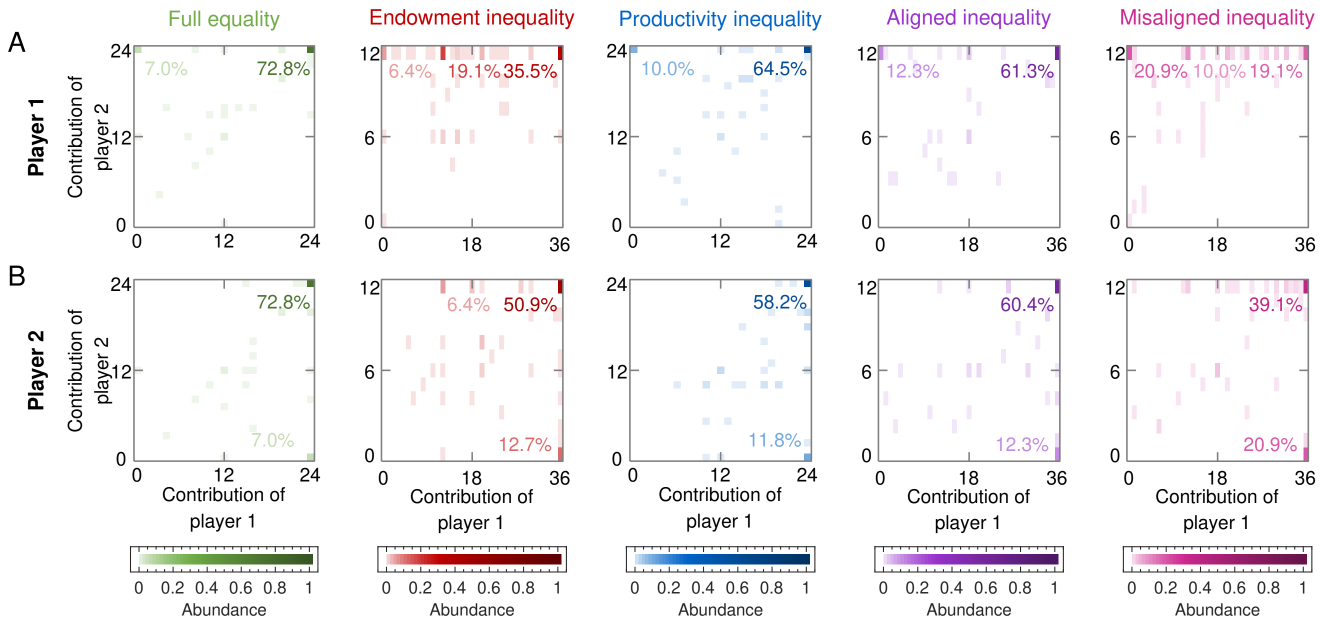


**Fig. S3. The preferred contribution pattern in the survey of the linear PGG.** We ask players regarding what contribution pattern they preferred. The figure illustrates the distribution of answers from players acting as player 1 (panel **A**) and player 2 (panel **B**), respectively. The numbers in the figure highlight the most preferred contribution patterns. In general, participants exhibit the strongest preference for full cooperation across all treatments. Their second preference is for patterns where their co-players fully contribute while they themselves make no contributions, maximizing their own payoff in that situation. Additionally, in the endowment inequality treatment and the misaligned inequality treatment, some high-endowment players also prefer that both of them make the same absolute contribution.

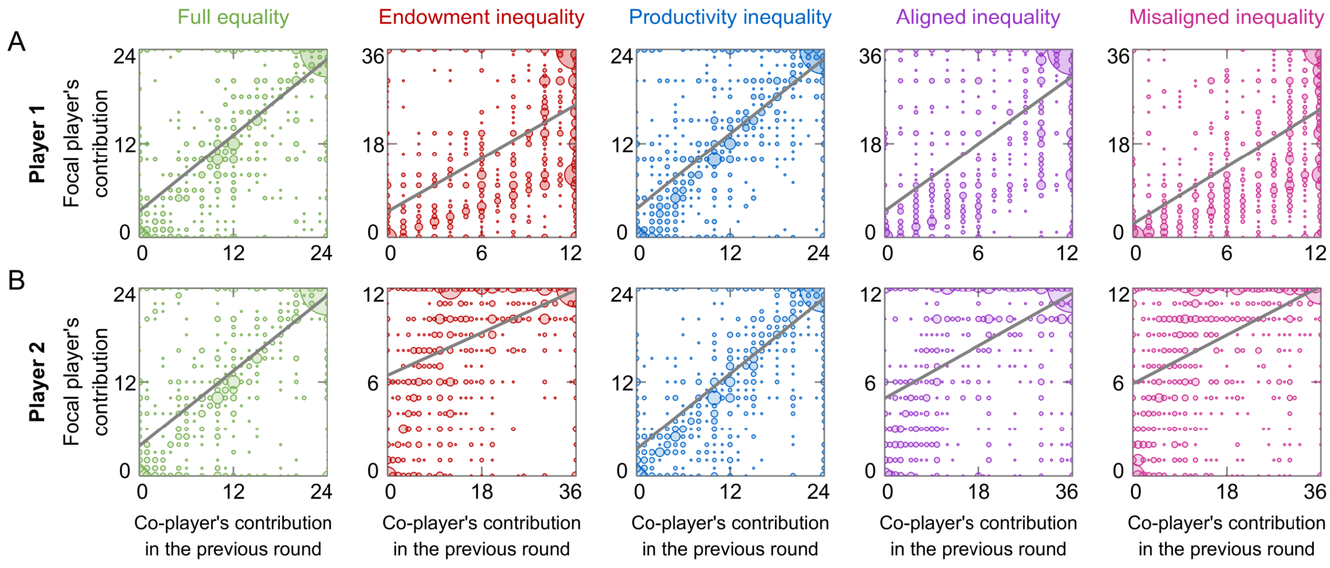


**Fig. S4. Conditional behaviors in the linear PGG experiment.** In this figure, we illustrate the contributions of a given player (on the $y$-axis), depending on their co-player's contribution in the previous round (on the $x$-axis). The bubble size measures how often we observe the respective reaction, across all players, groups, and rounds. The gray lines show the OLS regression results regarding conditional behaviors in the 20-round games, excluding the defective reaction $(0, 0)$. The positive slope indicates direct reciprocity among players.

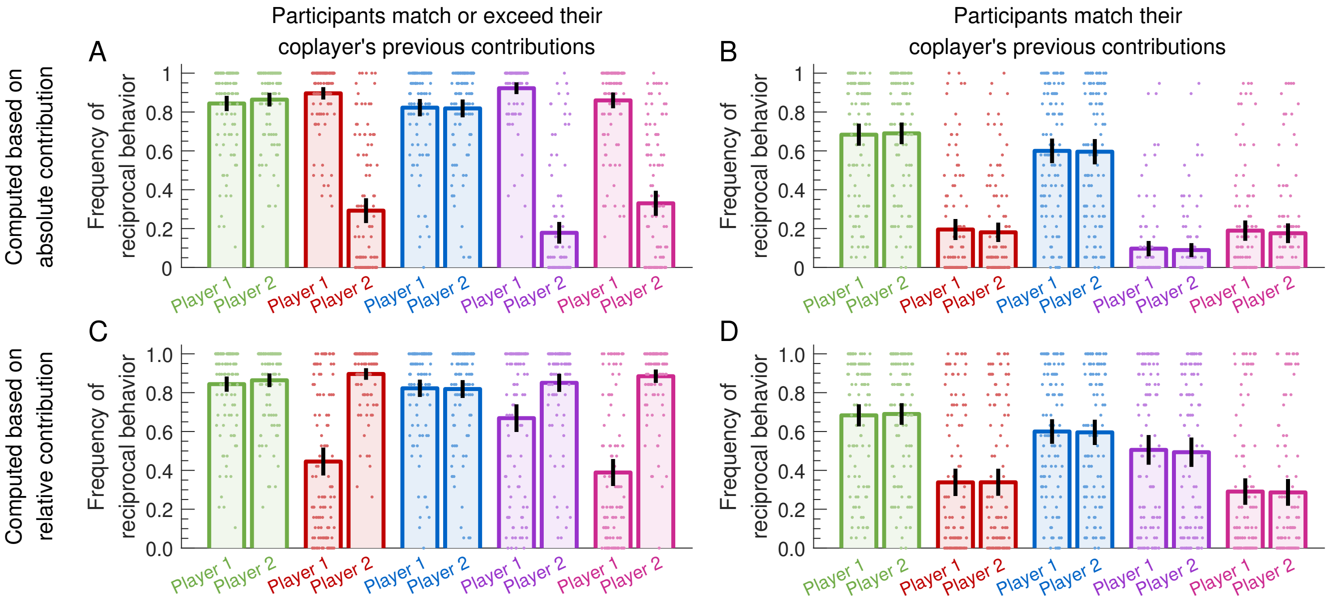


**Fig. S5. Abundance of reciprocal behaviors across the five treatments in the linear PGG.** In panels **A** and **C**, we present the fraction of rounds in which participants match or exceed their co-player's contribution from the previous round. In panels **B** and **D**, we show the fraction of rounds in which participants exactly match their co-player's contribution from the previous round. Error bars represent the respective 95% confidence intervals.

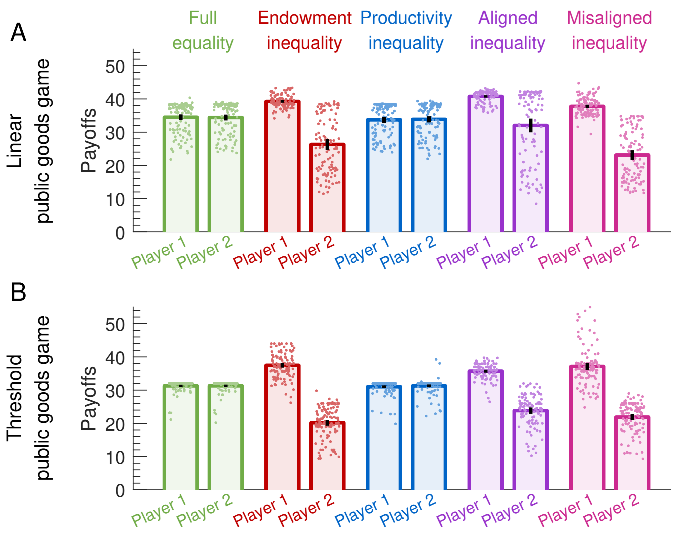


**Fig. S6. Payoffs of the two players in both games.** When players have the same initial endowment, there are no significant differences in their payoffs. However, when initial endowments differ, high-endowment players obtain significantly higher payoffs after the game.

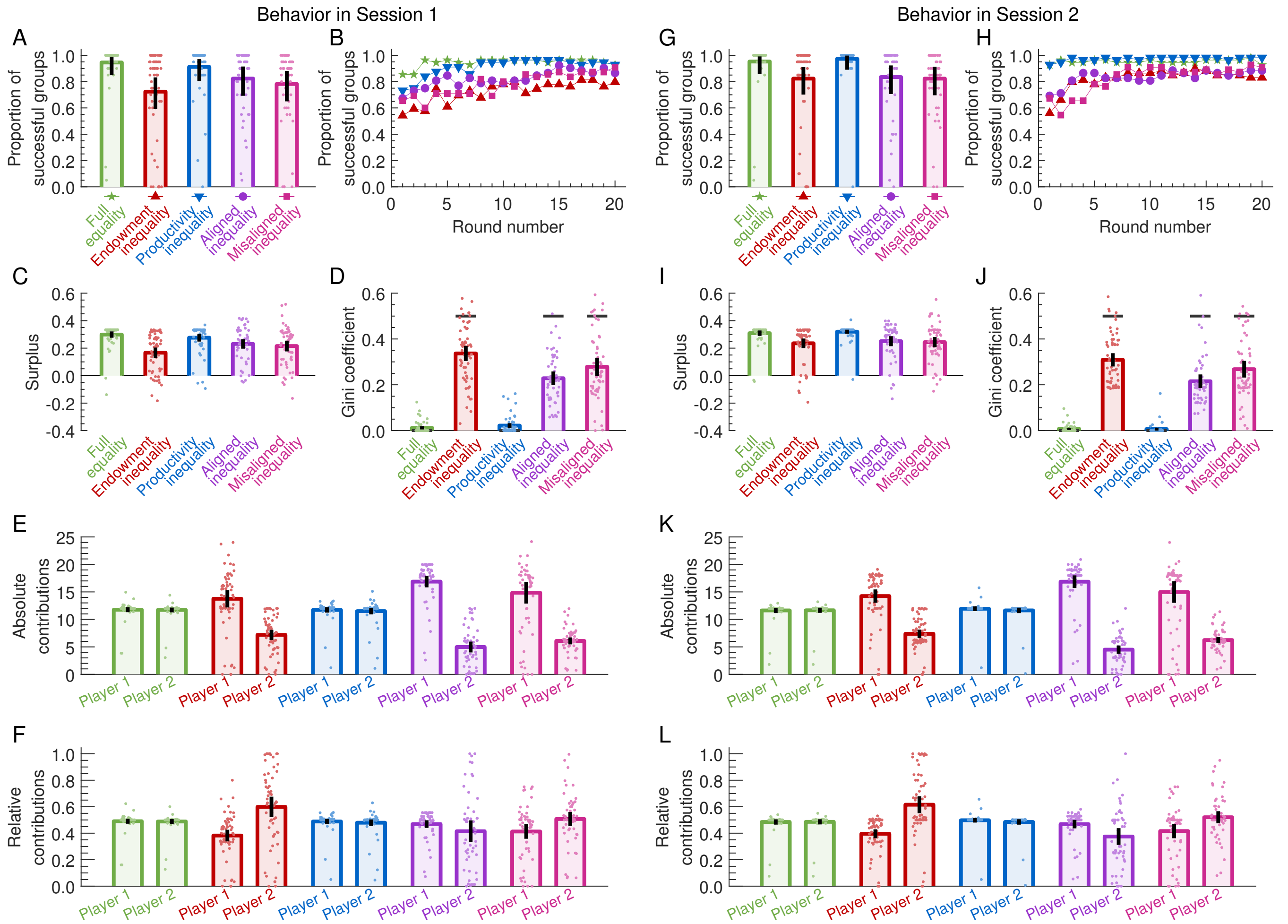


**Fig. S7. Experimental results from Session 1 and Session 2 in the threshold PGG.** The experiment featured two separate sessions, each consisting of 20 rounds with different partners for the participants. Here, the data from the first three treatments are sourced from Wang et al., 2023 (1), while the aligned inequality and misaligned inequality treatments are newly conducted in this work. We observe qualitative similarities in the results from both sessions. However, in the productivity inequality treatment, groups are significantly more successful in Session 2 (panels **A** and **G**), resulting in a higher generated surplus (panels **C** and **I**). This is because players almost immediately achieve successful coordination from the beginning of Session 2 (panels **B** and **H**). Error bars represent the respective 95% confidence intervals.

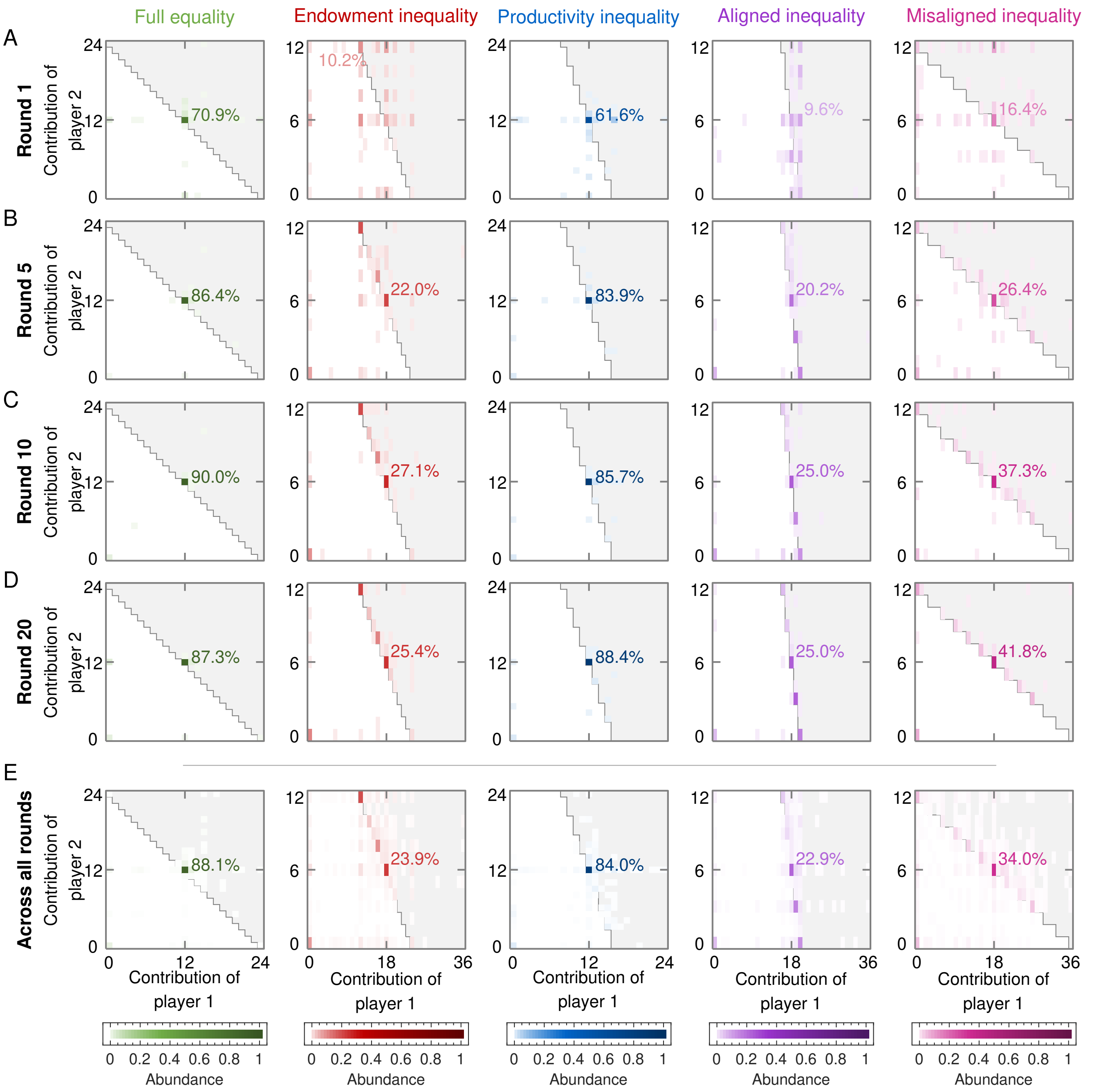


**Fig. S8. Cooperation dynamics over time in the threshold PGG.** In this figure, we present snapshots for 4 different time points (or round numbers, see rows 1-4), and the overall contribution patterns across all rounds (see the bottom row). These snapshots allow us to explore in more detail how players change their strategies over time. The intensity of shading within a rectangle reflects the frequency of the corresponding outcome observed across all groups, while the numbers indicate the most abundant outcome. In the threshold public goods game experiment, the most abundant outcome is that players make the same relative contribution (in proportion to their endowment). Different from the linear public goods game, the aligned inequality treatment does not exhibit a dominate contribution profile in the threshold public goods game.

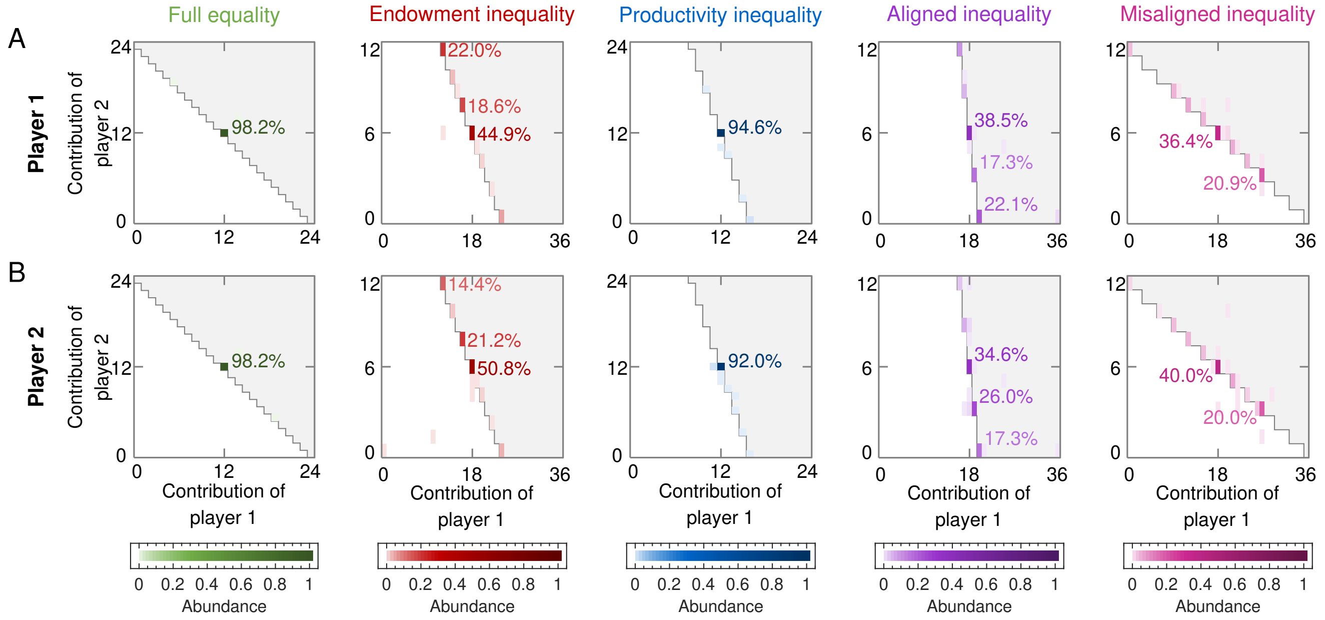


**Fig. S9. The most fair contribution pattern in the survey of the threshold PGG.** We ask players regarding what contribution pattern they consider as fair. The figure illustrates the distribution of answers from players acting as player 1 (panel **A**) and player 2 (panel **B**), respectively. In the aligned inequality and misaligned inequality treatments, most participants consider the outcome with equal relative contributions to be most fair, which is similar to the first three treatments.

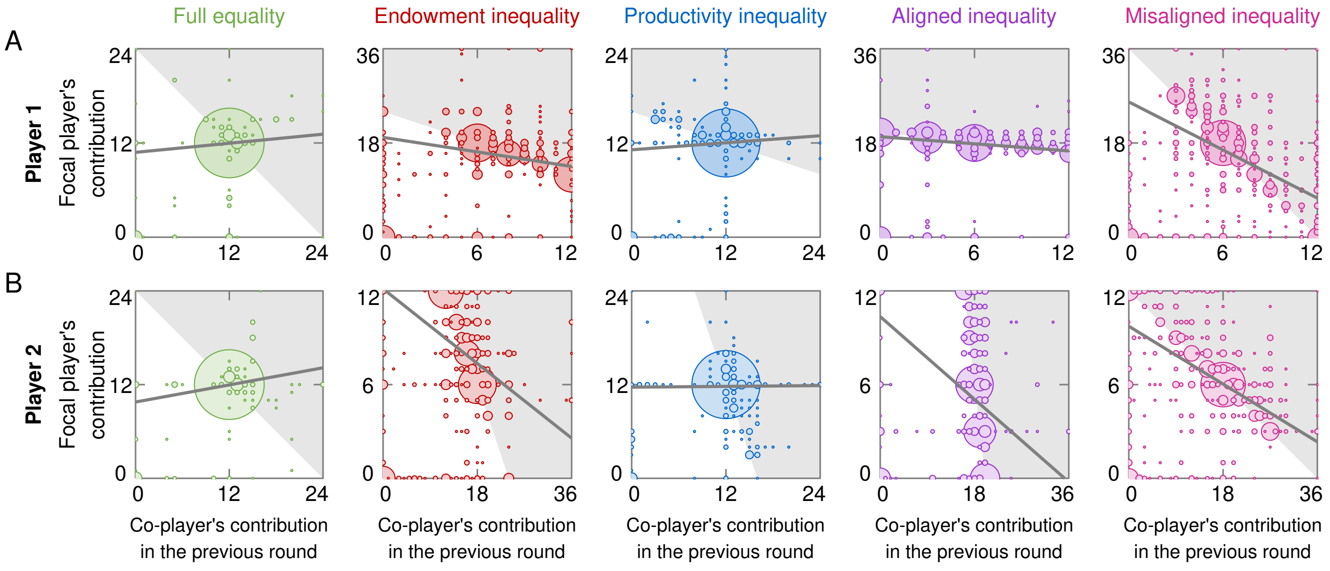


**Fig. S10. Conditional behaviors in the threshold PGG experiment.** In this figure, we illustrate the contributions of a given player (on the $y$-axis), depending on their co-player's contribution in the previous round (on the $x$-axis). The bubble size measures how often we observe the respective reaction, across all players, groups, and rounds. The gray lines show the OLS regression results regarding conditional behaviors in the 20-round games, excluding the defective reaction $(0, 0)$. The negative slope in the three treatments with unequal endowments indicates coordination among players.

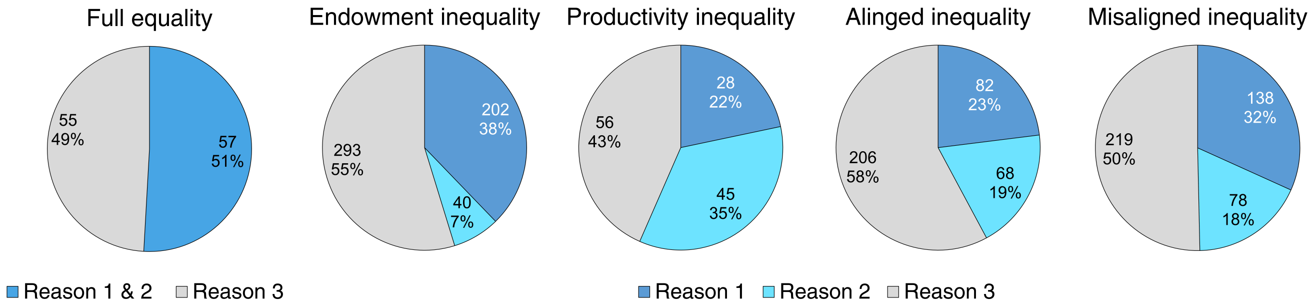


**Fig. S11. Reasons for failure in the five treatments.** There are three reasons for failure. Reason 1 is that the relative contribution of player 1 is less than one half, but the contribution of player 2 is no less than one half. Reason 2 is that the relative contribution of player 2 is less than one half, but the contribution of player 1 is no less than one half. Reason 3 is that the relative contributions of both players are less than one half. For each of these reasons, the absolute numbers represent the quantities of the corresponding contribution profiles observed in the experiment.

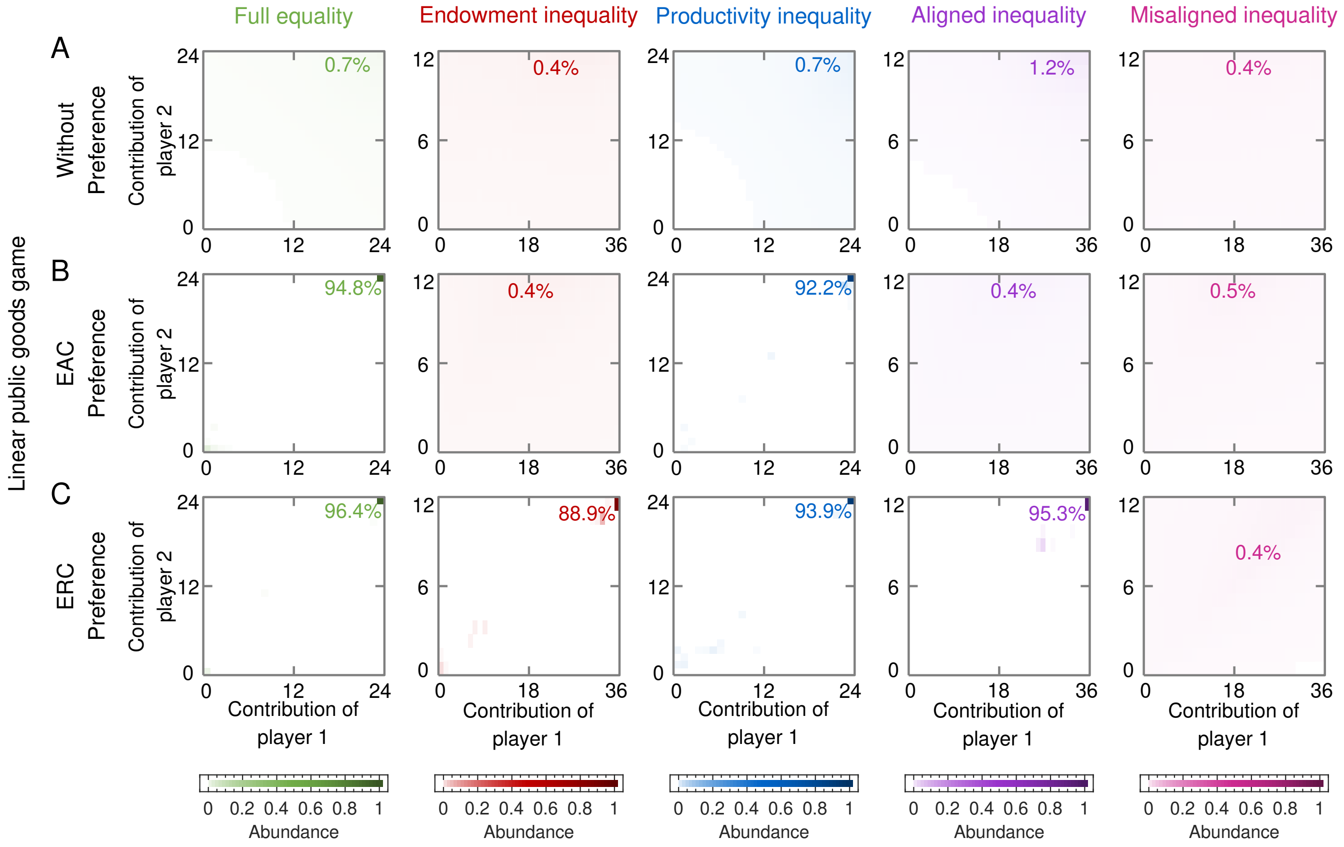


**Fig. S12. Comparing the effects of different preferences in the linear public goods game.** This figure illustrates the effects of a single preference on the dynamics of reactive strategies in the linear game. Each panel displays the distribution of contribution profiles for a pair of players throughout the entire simulation process. Simulations are run for $10^7$ time steps with a selection strength of $s = 100$ across all panels. Preference strengths: **(A)** $\beta = \gamma = 0$; **(B)** $\beta = 10$ and $\gamma = 0$; **(C)** $\beta = 0$ and $\gamma = 10$.

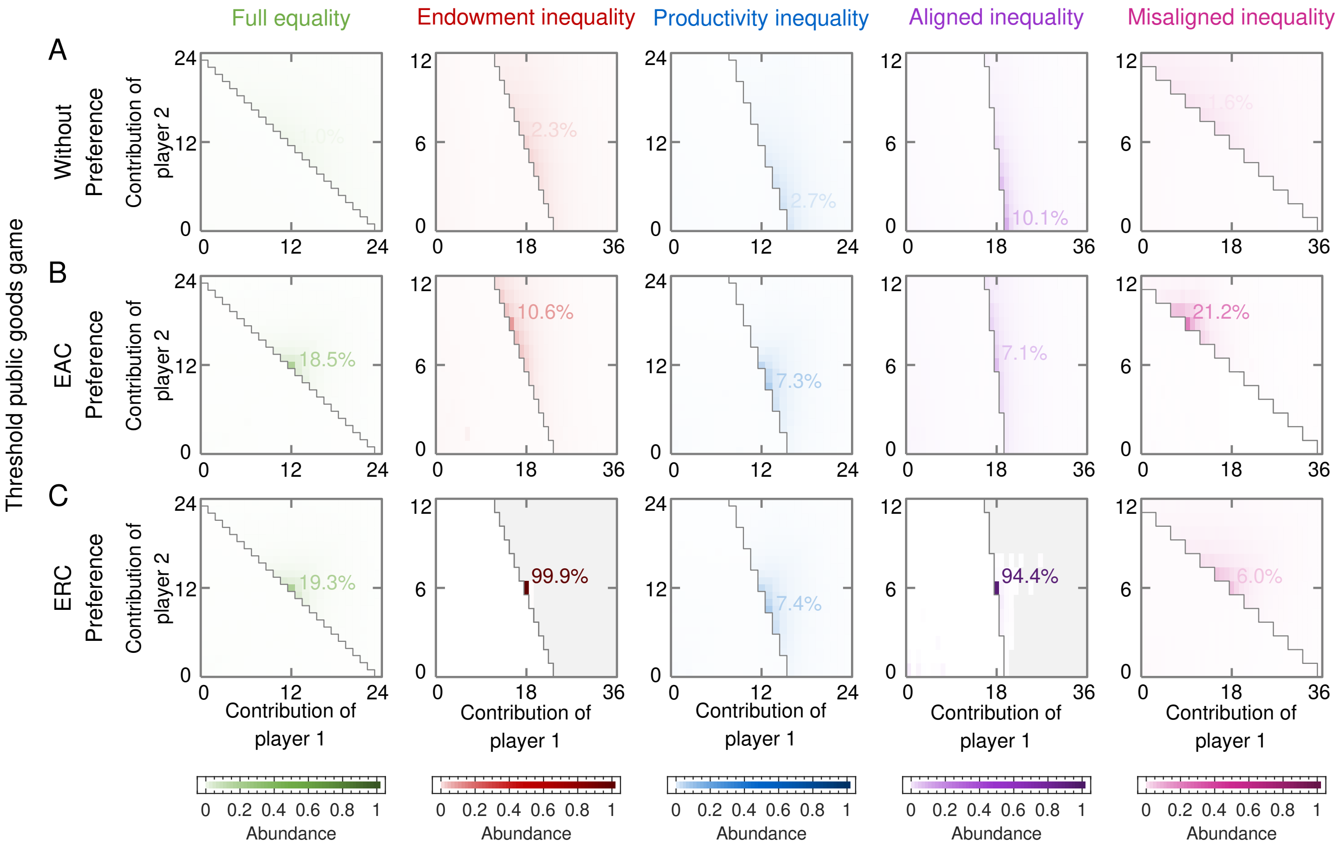


**Fig. S13. Comparing the effects of different preferences in the threshold public goods game.** This figure illustrates the effects of a single preference on the dynamics of reactive strategies in the threshold game. Each panel displays the distribution of contribution profiles for a pair of players throughout the entire simulation process. Simulations are run for $10^7$ time steps with a selection strength of $s = 100$ across all panels. Preference strengths: **(A)** $\beta = \gamma = 0$; **(B)** $\beta = 10$ and $\gamma = 0$; **(C)** $\beta = 0$ and $\gamma = 10$.

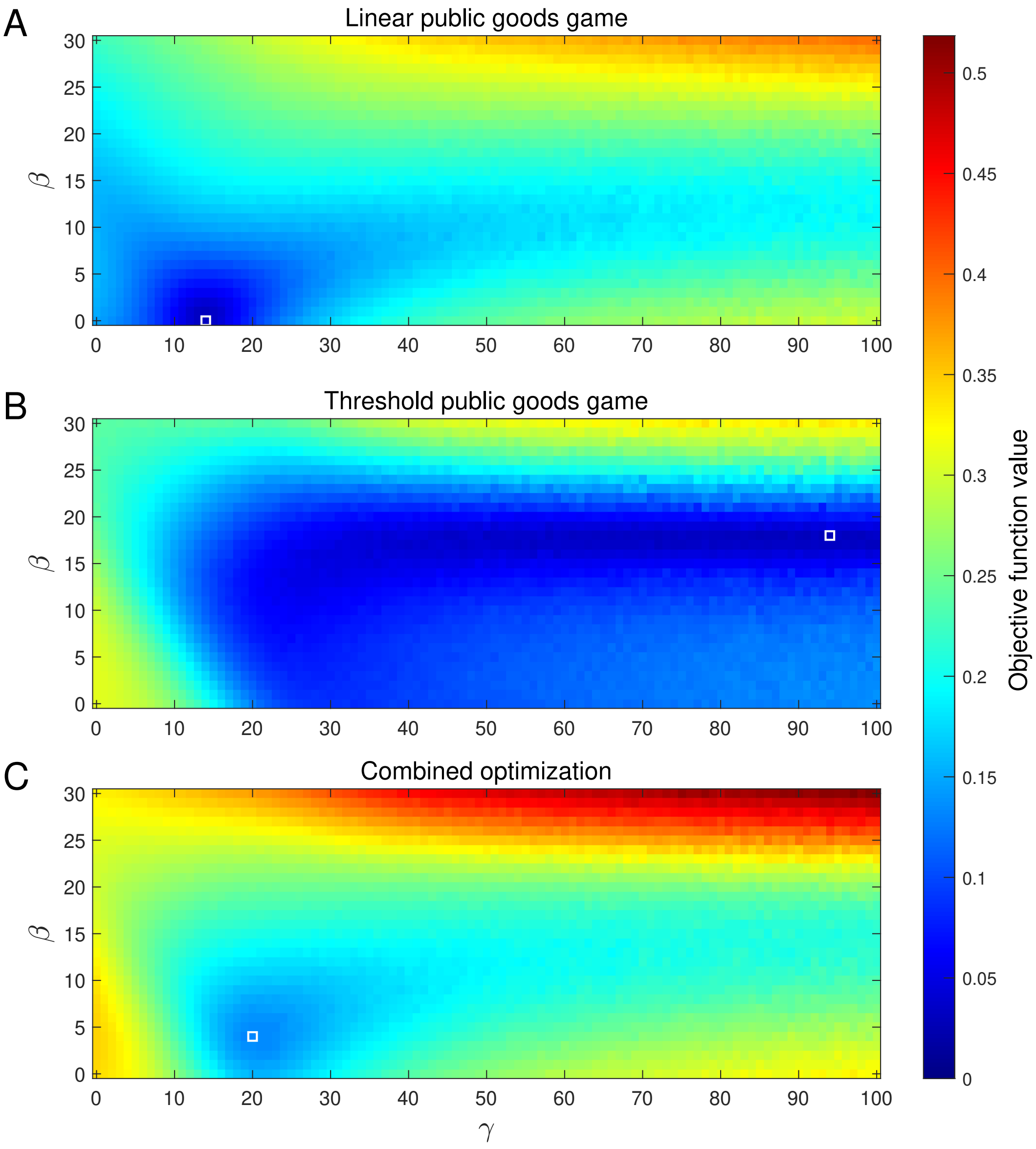


**Fig. S14. Objective function landscapes for parameter estimation.** This figure shows the simulated values of the objective functions under selection strength $s = 1$, where the best-fitting parameters are obtained. **(A)** For the linear public goods game, the objective function $\Delta_{GOS}$ is defined in $\mathrm{Eq.}$ (7). **(B)** For the threshold public goods game, the same objective function $\Delta_{GOS}$ is used. **(C)** For the combined optimization of both games, the objective function $\Delta_{MGOS}$ is defined in $\mathrm{Eq.}$ (8). In each panel, the white box marks the $(\gamma, \beta)$ that best fit the experimental data: (A) $(14, 0)$, (B) $(94, 18)$, and (C) $(20, 4)$.

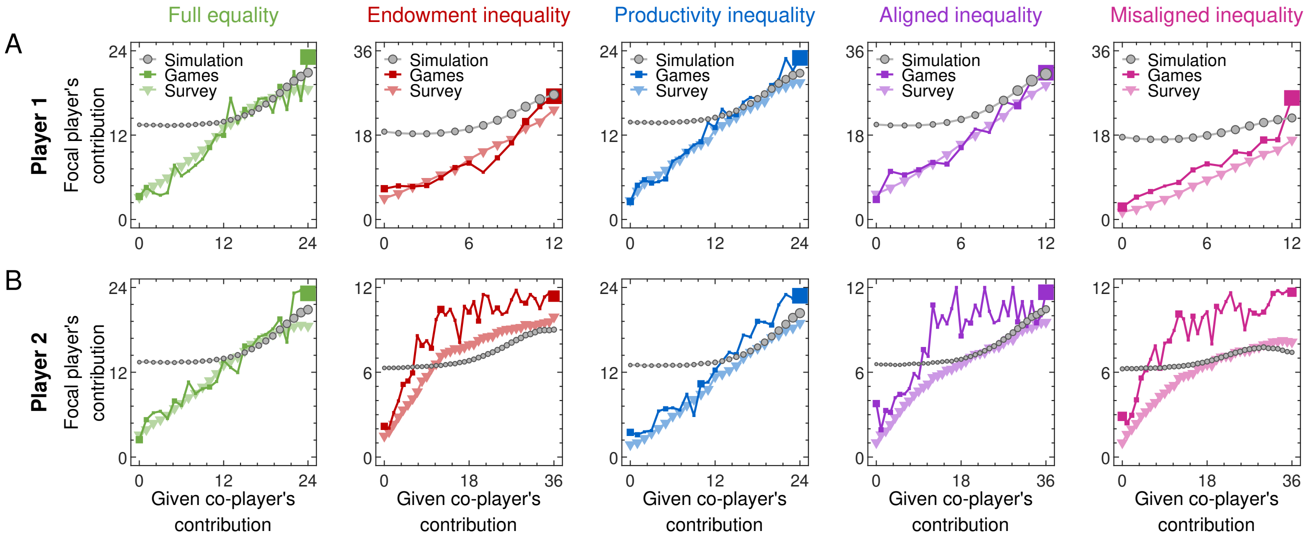


**Fig. S15. Conditional behaviors in the linear PGG.** In this figure, we illustrate the conditional behaviors observed in the 20-round games during the experiment (represented by square symbols), the responses to given co-player contributions in the survey (represented by inverted triangle symbols), and the conditional behaviors across $10^7$ time steps in the simulation (represented by gray bubbles). The simulation utilize the optimal parameters selected for predicting experimental results, including a selection strength $s = 1$ and preference strengths $(\gamma, \beta) = (14, 0)$. The larger the size of each symbol, the higher the frequency of the respective behavior. When the co-player's relative contribution exceeds half, the conditional behavior observed in the simulation and in the survey align well.

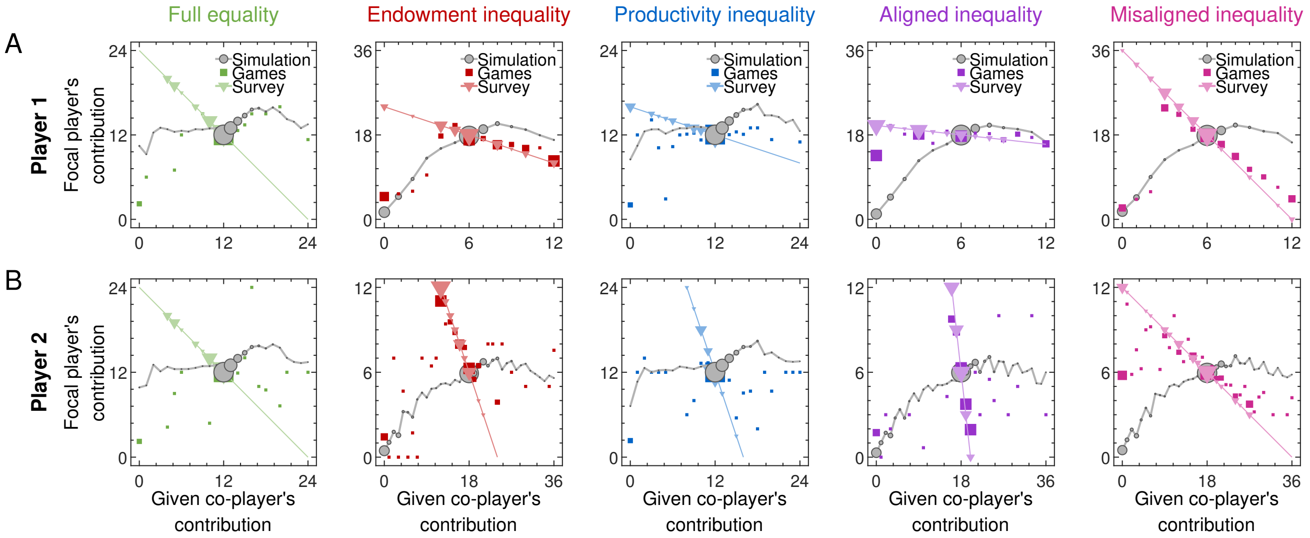


**Fig. S16. Conditional behaviors in the threshold PGG.** In this figure, we illustrate the conditional behaviors observed in the 20-round games during the experiment (represented by square symbols), the expected co-player's minimum contributions for successful coordination in the survey (represented by inverted triangle symbols), and the conditional behaviors across $10^7$ time steps in the simulation (represented by gray bubbles). The simulation utilize the optimal parameters selected for predicting experimental results, including a selection strength $s = 1$ and preference strengths $(\gamma, \beta) = (94, 18)$. The larger the size of each symbol, the higher the frequency of the respective behavior. The simulation gives a good approximation of the frequently occurring conditional contributions observed in the experiment.

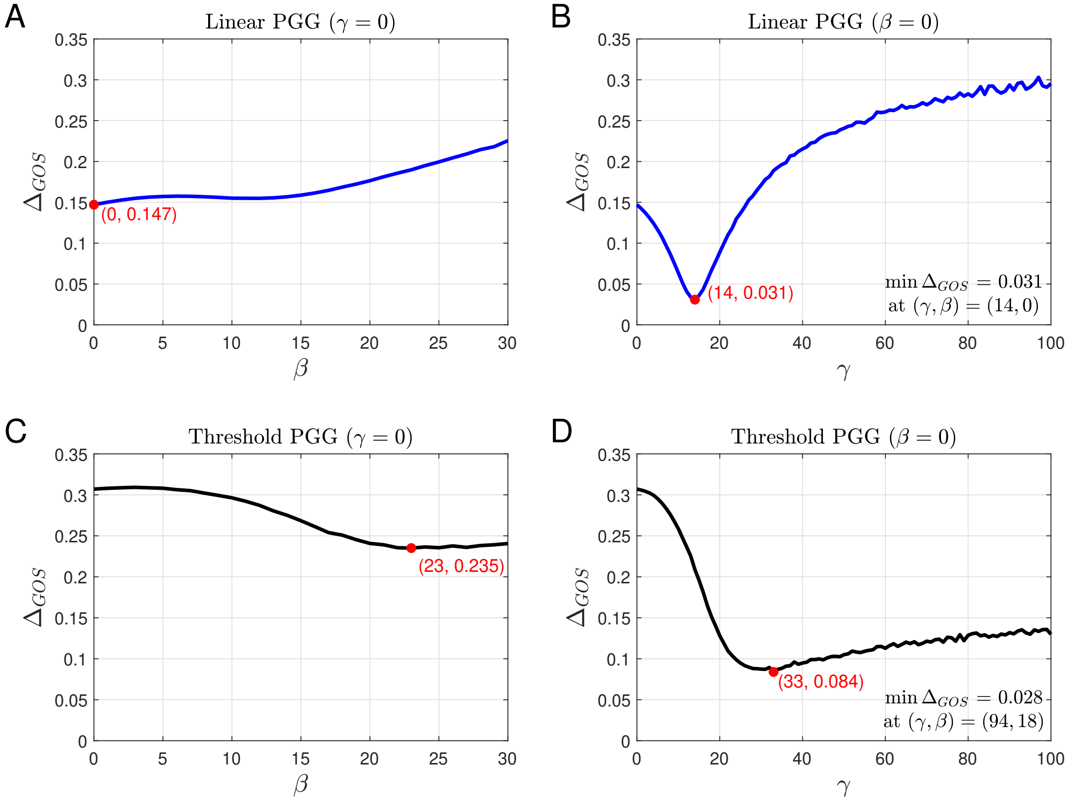


**Fig. S17. Effects of single-parameter variation on the objective function $\Delta_{GOS}$.** Each panel shows the simulated values of the objective function defined in Eq. (7) under selection strength $s = 1$. The top row corresponds to the linear public goods game, and the bottom row to the threshold public goods game. The left column plots $\Delta_{GOS}$ against $\beta$ when $\gamma = 0$, corresponding to the preference for equal absolute contributions (EAC). The right column plots $\Delta_{GOS}$ against $\gamma$ when $\beta = 0$, corresponding to the preference for equal relative contributions (ERC). In each panel, the red dot marks the parameter value yielding the minimum $\Delta_{GOS}$, with the corresponding coordinate displayed next to the point. For the two panels in the right column, an additional annotation in the bottom-right corner reports $\min \Delta_{GOS}$ achievable with the full parameter set, for comparison with the single-parameter case.

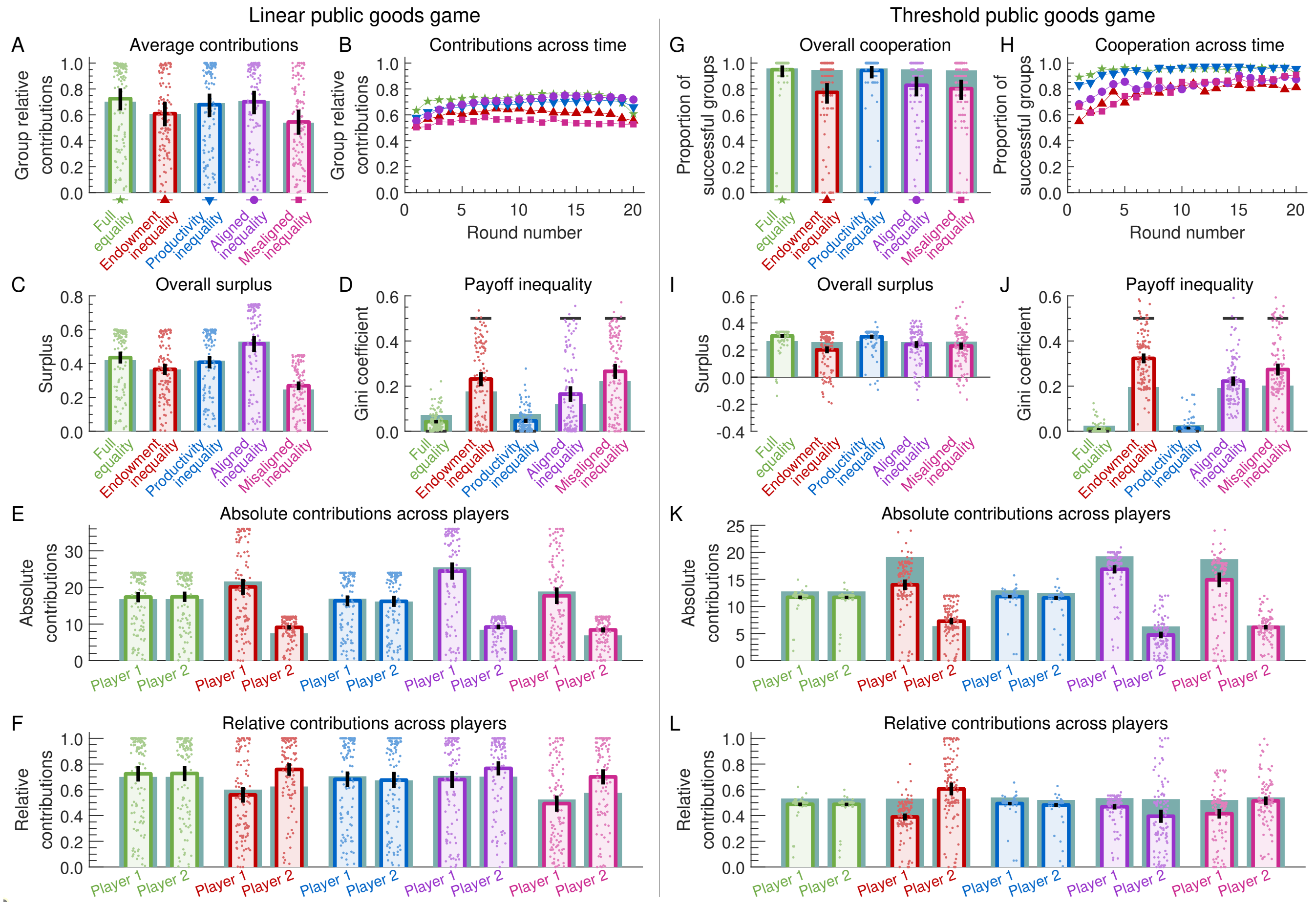


**Fig. S18. Comparison between experimental results and simulations using the combined-optimal parameters.** Colored bars are the same as in **Fig. 3** in the main text, while the light teal bars show the simulated outcomes obtained under selection strength $s = 1$ and preference strengths $(\gamma, \beta) = (20, 4)$. This parameter combination represents the combined optimum that minimizes $\Delta_{MGOS}$ (Eq. (8)), with the same set of parameters applied to all ten treatments. However, this parameter combination fails to reproduce some of the behavioral patterns in either game type, as shown by substantial deviations from experimental benchmarks. In the linear game, the simulations overestimate contribution levels in the full equality treatment and the productivity inequality treatment. In the threshold game, the simulations overestimate contributions in all three treatments with unequal endowments.

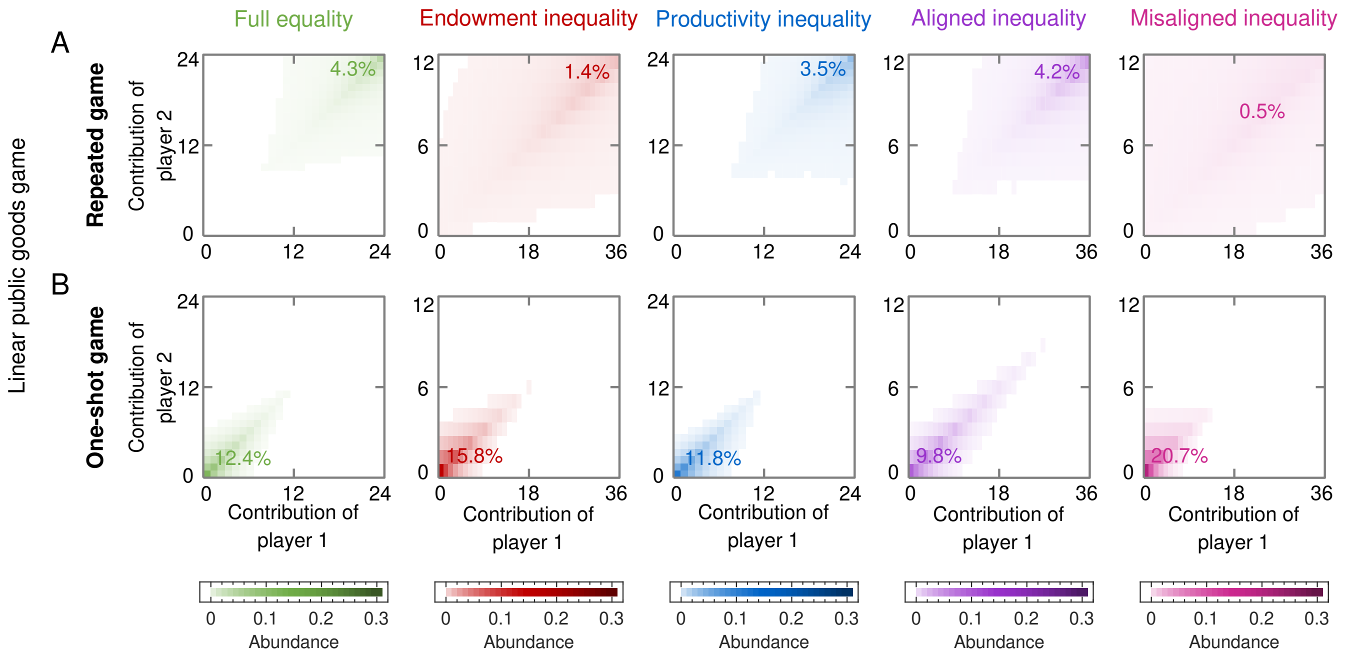


**Fig. S19. Distribution of actions in the linear game under repeated and one-shot games.** (**A**) In the repeated game, player use reactive strategies. The simulation results closely match experimental observations in terms of the average group collective contributions and surplus. (**B**) In the one-shot game, players adopt unconditional strategies. Repeated interactions promote cooperation among players. Both simulations are run for $10^7$ time steps with a selection strength $s = 1$ and preference strengths $(\gamma, \beta) = (14, 0)$.

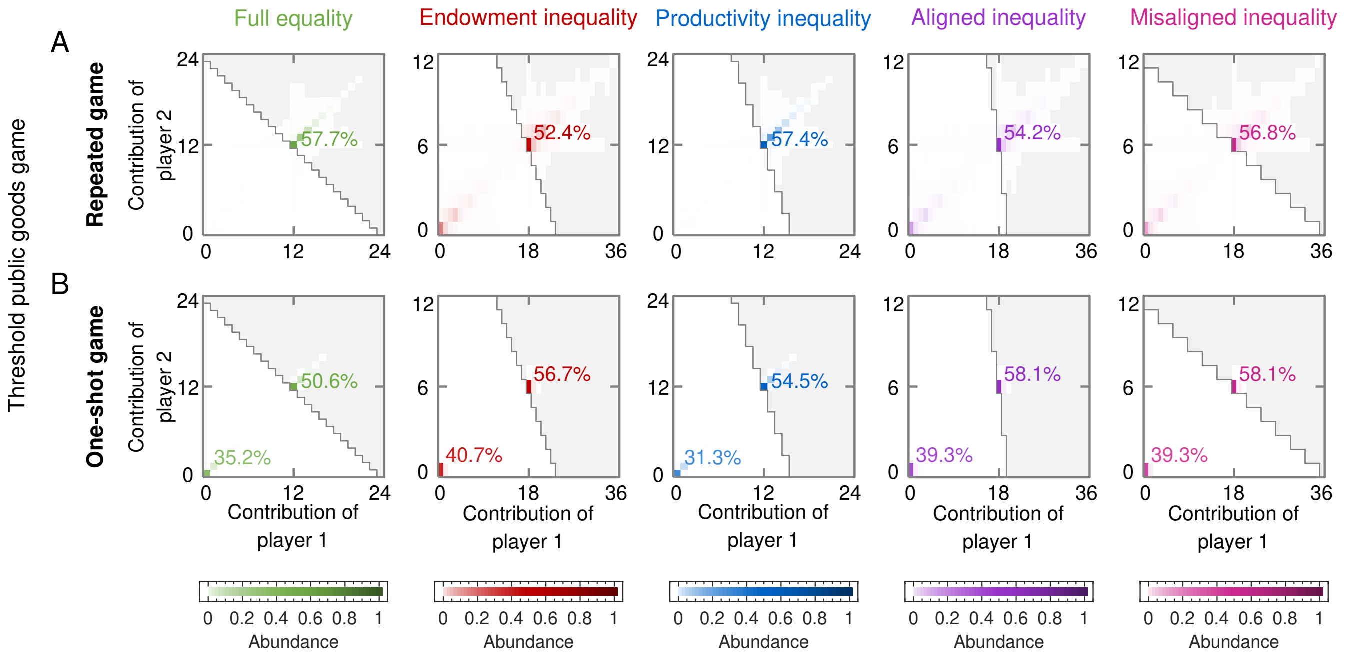


**Fig. S20. Distribution of actions in the threshold game under repeated and one-shot games.** (**A**) In the repeated game, player use reactive strategies. The simulation results closely match experimental observations in terms of the average group success rate and surplus. (**B**) In the one-shot game, players adopt unconditional strategies. The abundance of full defection increases significantly in the absence of repeated interactions. The simulations are run for $10^7$ time steps with a selection strength of $s = 1$ and preference strengths $(\gamma, \beta) = (94, 18)$, averaged over 1000 iterations.

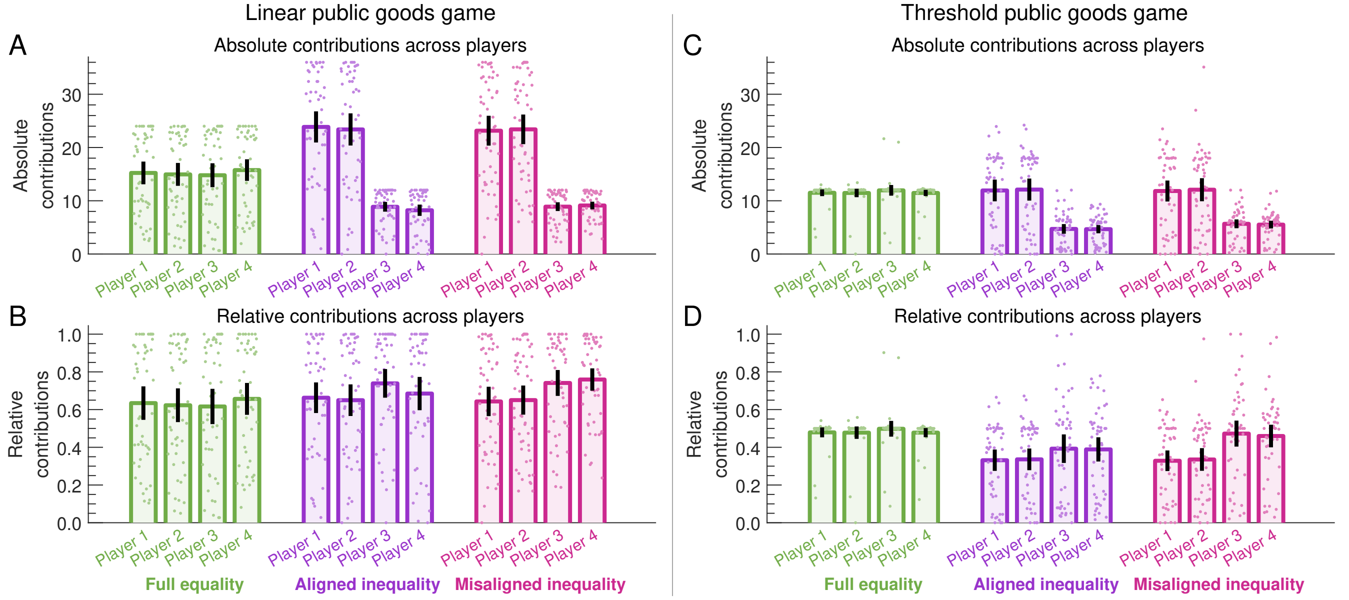


**Fig. S21. Contributions of the four players in both games.** This figure illustrates the contributions of four players in each treatment. Each dot represents a player. Panels **A** and **B** show results from the linear game, while panels **C** and **D** show results from the threshold game. In both games, under full equality, there is no significant difference in contributions among group members. By contrast, in other treatments, players with the same role show no significant differences in contributions, while players with different roles exhibit significant differences in contributions, except under aligned inequality in panel **D** where no significant differences are observed between different-role players.

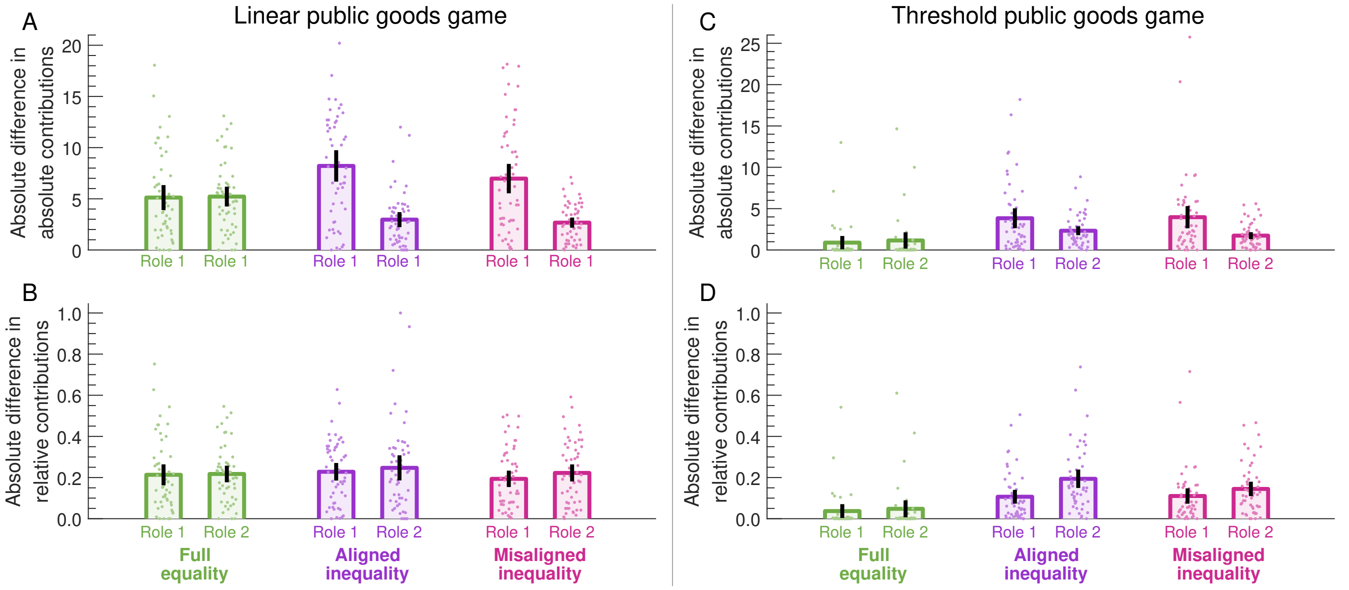


**Fig. S22. Contribution differences between same-role players.** Each point represents one group. For each group, we compute the absolute difference in contributions between the two players sharing the same role in each round, and then take the average over 20 rounds. Panels **A** and **C** use absolute contributions, while panels **B** and **D** use relative contributions. Panels **A** and **B** correspond to the linear game; panels **C** and **D** correspond to the threshold game.

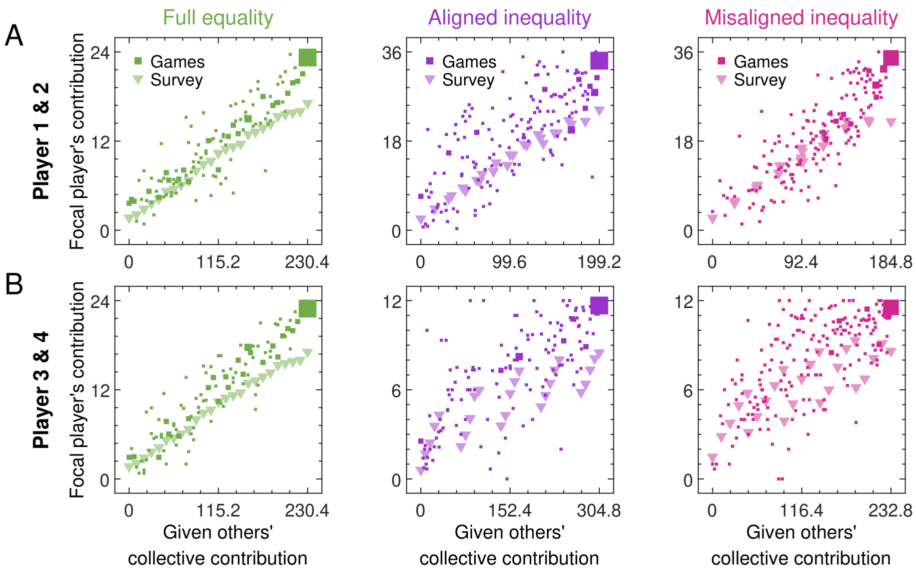


**Fig. S23. Conditional behaviors in the four-player linear PGG.** For both game and post-game survey, we examine each focal player's absolute contribution $c_i$ as a function of the collective contributions of the other three players, calculated as $\sum_{j\neq i} p_j c_j$, where $c_j$ is the absolute contribution and $p_j$ is the productivity of player $j$. The larger the size of each symbol, the higher the frequency of the respective behavior. We observe players' reciprocity behaviors.

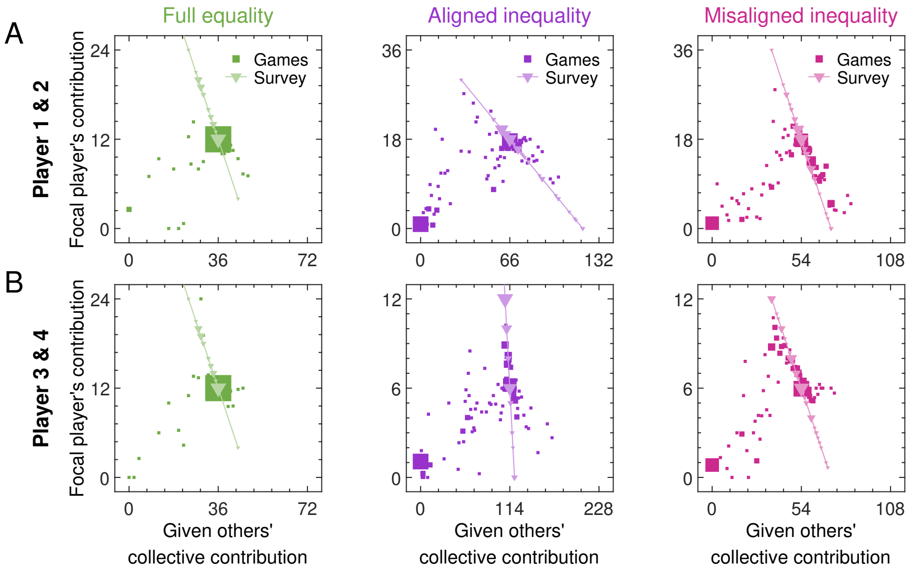


**Fig. S24. Conditional behaviors in the four-player threshold PGG.** For both game and post-game survey, we examine each focal player's absolute contribution $c_i$ as a function of the collective contributions of the other three players, calculated as $\sum_{j\neq i} p_j c_j$, where $c_j$ is the absolute contribution and $p_j$ is the productivity of player $j$. The larger the size of each symbol, the higher the frequency of the respective behavior.

| **Without** $(0, 0)$ | | Full equality | | Endowment inequality | | Productivity inequality | | Aligned inequality | | Misaligned inequality | |
|---|---|---|---|---|---|---|---|---|---|---|---|
| | | Player 1 | Player 2 | Player 1 | Player 2 | Player 1 | Player 2 | Player 1 | Player 2 | Player 1 | Player 2 |
| Linear PGG | Coplayer's previous contribution | 0.8100*** | 0.7990*** | 1.7060*** | 0.1507*** | 0.7990*** | 0.8023*** | 2.1511*** | 0.1856*** | 1.8349*** | 0.1721*** |
| | | (0.0143) | (0.0138) | (0.0673) | (0.0057) | (0.0138) | (0.0139) | (0.0591) | (0.0051) | (0.0618) | (0.0060) |
| | Constant | 3.3576*** | 3.8377*** | 4.9637*** | 6.4264*** | 3.6720*** | 3.4301*** | 5.0645*** | 4.9687*** | 2.5759*** | 5.8957*** |
| | | (0.2900) | (0.2770) | (0.6920) | (0.1407) | (0.2620) | (0.2662) | (0.6092) | (0.1462) | (0.6231) | (0.1395) |
| | Observations | 2048 | 2047 | 1989 | 1989 | 1974 | 1972 | 1942 | 1947 | 1901 | 1907 |
| | R-squared | 0.609 | 0.623 | 0.244 | 0.260 | 0.631 | 0.630 | 0.406 | 0.402 | 0.317 | 0.303 |
| Threshold PGG | Coplayer's previous contribution | 0.0964*** | 0.1827*** | -0.4629*** | -0.2656*** | 0.0746** | 0.0085 | -0.2259*** | -0.2941*** | -1.5312*** | -0.2056*** |
| | | (0.0253) | (0.0185) | (0.0304) | (0.0145) | (0.0210) | (0.0216) | (0.0216) | (0.0236) | (0.0549) | (0.0062) |
| | Constant | 10.8060*** | 9.7711*** | 19.0670*** | 12.1310*** | 11.1300*** | 11.6700*** | 19.1940*** | 10.3540*** | 25.8420*** | 9.7374*** |
| | | (0.3052) | (0.2231) | (0.2615) | (0.2331) | (0.2491) | (0.2621) | (0.1342) | (0.4339) | (0.3854) | (0.1085) |
| | Observations | 2041 | 2042 | 2039 | 2032 | 2094 | 2094 | 1839 | 1844 | 1970 | 1967 |
| | R-squared | 0.007 | 0.046 | 0.102 | 0.142 | 0.006 | $<$0.001 | 0.056 | 0.078 | 0.283 | 0.359 |

Results from the OLS regression. Standard errors in parentheses. *** $p < 0.0001$; ** $p < 0.001$.

**Table S25. Linear regression results of players' conditional behavior in promoting cooperation.** This table presents the results of linear regressions exploring how players' absolute contributions are affected by their co-players' absolute contributions from the previous round. In each type of the game, the regressions are conducted across five different treatments, with each treatment analyzed separately for the two players. To analyze players' behavior in promoting cooperation, we exclude instances of defection equilibrium $(0, 0)$ before running the regressions.

| **With** $(0, 0)$ | | Full equality | | Endowment inequality | | Productivity inequality | | Aligned inequality | | Misaligned inequality | |
|---|---|---|---|---|---|---|---|---|---|---|---|
| | | Player 1 | Player 2 | Player 1 | Player 2 | Player 1 | Player 2 | Player 1 | Player 2 | Player 1 | Player 2 |
| Linear PGG | Coplayer's previous contribution | 0.8525*** | 0.8465*** | 1.8349*** | 0.1878*** | 0.8404*** | 0.8423*** | 2.2460*** | 0.2072*** | 1.9270*** | 0.2227*** |
| | | (0.0123) | (0.0119) | (0.0571) | (0.0056) | (0.0121) | (0.0121) | (0.0528) | (0.0050) | (0.0483) | (0.0058) |
| | Constant | 2.4261*** | 2.7959*** | 3.5449*** | 5.3663*** | 2.7981*** | 2.5800*** | 4.0087*** | 4.2782*** | 1.5672*** | 4.4807*** |
| | | (0.2419) | (0.2328) | (0.5726) | (0.1358) | (0.2249) | (0.2266) | (0.5342) | (0.1388) | (0.4643) | (0.1286) |
| | Observations | 2166 | 2166 | 2090 | 2090 | 2090 | 2090 | 2014 | 2014 | 2090 | 2090 |
| | R-squared | 0.690 | 0.701 | 0.331 | 0.347 | 0.696 | 0.698 | 0.474 | 0.464 | 0.432 | 0.416 |
| Threshold PGG | Coplayer's previous contribution | 0.7281*** | 0.7001*** | 0.4280*** | 0.1555*** | 0.4887*** | 0.4495*** | 0.2513*** | 0.0837*** | -0.4561*** | -0.0739*** |
| | | (0.0164) | (0.0140) | (0.0334) | (0.0125) | (0.0186) | (0.0190) | (0.0342) | (0.0146) | (0.0596) | (0.0077) |
| | Constant | 3.1625*** | 3.4855*** | 10.8400*** | 5.0938*** | 6.1599*** | 6.2622*** | 15.5950*** | 3.2889*** | 17.7400*** | 7.1991*** |
| | | (0.1954) | (0.1669) | (0.2742) | (0.1915) | (0.2195) | (0.2285) | (0.2051) | (0.2600) | (0.4061) | (0.1302) |
| | Observations | 2090 | 2090 | 2242 | 2242 | 2128 | 2128 | 1976 | 1976 | 2090 | 2090 |
| | R-squared | 0.485 | 0.545 | 0.068 | 0.065 | 0.245 | 0.209 | 0.027 | 0.016 | 0.027 | 0.0426 |

Results from the OLS regression. Standard errors in parentheses. *** $p < 0.0001$; ** $p < 0.001$.

**Table S26. Linear regression results of players' conditional behavior.** Same as Table S25 but no data point is excluded.

## References


1. X Wang, et al., Cooperation and coordination in heterogeneous populations. *Philos. Trans. R. Soc. B Biol. Sci.* **378**, 20210504 (2023).
2. O Hauser, C Hilbe, K Chatterjee, M Nowak, Social dilemmas among unequals. *Nature* **572**, 524—527 (2019).
3. M Perc, A Szolnoki, G Szabó, Restricted connections among distinguished players support cooperation. *Phys. Rev. E* **78**, 066101 (2008).
4. M Perc, Does strong heterogeneity promote cooperation by group interactions? *New J. Phys.* **13**, 123027 (2011).
5. J Qin, Y Chen, Y Kang, M Perc, Social diversity promotes cooperation in spatial multigames. *Europhys. Lett.* **118**, 18002 (2017).
6. D Fudenberg, J Tirole, *Game Theory.* (MIT Press, Cambridge), 6th edition, (1998).
7. MA Nowak, K Sigmund, The evolution of stochastic strategies in the prisoner's dilemma. *Acta Appl. Math.* **20**, 247–265 (1990).
8. LA Imhof, MA Nowak, Stochastic evolutionary dynamics of direct reciprocity. *Proc. Royal Soc. B: Biol. Sci.* **277**, 463–468 (2010).
9. SK Baek, HC Jeong, C Hilbe, MA Nowak, Comparing reactive and memory-one strategies of direct reciprocity. *Sci. Reports* **6**, 25676 (2016).
10. G Molnar, C Hammond, F Fu, Reactive means in the iterated prisoner's dilemma. *Appl. Math. Comput.* **458**, 128201 (2023).
11. NE Glynatsi, A McAvoy, C Hilbe, Evolution of reciprocity with limited payoff memory. *Proc. Royal Soc. B: Biol. Sci.* **291**, 20232493 (2024).
12. E Fehr, K Schmidt, A theory of fairness, competition, and cooperation. *Q. J. Econ.* **114**, 817–868 (1999).
13. MC Couto, S Giaimo, C Hilbe, Introspection dynamics: A simple model of counterfactual learning in asymmetric games. *New J. Phys.* **24**, 063010 (2022).
14. MC Couto, S Pal, Introspection dynamics in asymmetric multiplayer games. *Dyn. Games Appl.* **13**, 1256–1285 (2023).
15. X Wang, C Hilbe, B Zhang, Dataset and computer code for "the dynamics of cooperation in asymmetric public goods games" (https://doi.org/10.5281/zenodo.16918146) (2025).
